# Intake of sugar sweetened beverages among children and adolescents in 185 countries between 1990 and 2018: population based study

**DOI:** 10.1136/bmj-2024-079234

**Published:** 2024-08-07

**Authors:** Laura Lara-Castor, Renata Micha, Frederick Cudhea, Victoria Miller, Peilin Shi, Jianyi Zhang, Julia R Sharib, Josh Erndt-Marino, Sean B Cash, Simon Barquera, Dariush Mozaffarian, Global Dietary Database, Antonia Trichopoulou, Murat Bas, Jemal Haidar Ali, Tatyana El-Kour, Anand Krishnan, Puneet Misra, Nahla Hwalla, Chandrashekar Janakiram, Nur Indrawaty Lipoeto, Abdulrahman Musaiger, Farhad Pourfarzi, Iftikhar Alam, Celine Termote, Anjum Memon, Marieke Vossenaar, Paramita Mazumdar, Ingrid Rached, Alicia Rovirosa, María Elisa Zapata, Roya Kelishadi, Tamene Taye Asayehu, Francis Oduor, Julia Boedecker, Lilian Aluso, Emanuele Marconi, Laura D’Addezio, Raffaela Piccinelli, Stefania Sette, Johana Ortiz-Ulloa, J V Meenakshi, Giuseppe Grosso, Anna Waskiewicz, Umber S Khan, Kenneth Brown, Lene Frost Andersen, Anastasia Thanopoulou, Reza Malekzadeh, Neville Calleja, Anca Ioana Nicolau, Cornelia Tudorie, Marga Ocke, Zohreh Etemad, Mohannad Al Nsour, Lydiah M Waswa, Maryam Hashemian, Eha Nurk, Joanne Arsenault, Patricio Lopez-Jaramillo, Abla Mehio Sibai, Albertino Damasceno, Pulani Lanerolle, Carukshi Arambepola, Carla Lopes, Milton Severo, Nuno Lunet, Duarte Torres, Heli Tapanainen, Jaana Lindstrom, Suvi Virtanen, Cristina Palacios, Noel Barengo, Eva Roos, Irmgard Jordan, Charmaine Duante, Corazon Cerdena, Imelda Angeles-Agdeppa, Josie Desnacido, Mario Capanzana, Anoop Misra, Ilse Khouw, Swee Ai Ng, Edna Gamboa Delgado, Mauricio T Caballero, Johanna Otero, Hae-Jeung Lee, Eda Koksal, Idris Guessous, Carl Lachat, Stefaan De Henauw, Ali Reza Rahbar, Alison Tedstone, Annie Ling, Beth Hopping, Catherine Leclercq, Christian Haerpfer, Christine Hotz, Christos Pitsavos, Coline van Oosterhout, Debbie Bradshaw, Dimitrios Trichopoulos, Dorothy Gauci, Dulitha Fernando, Elzbieta Sygnowska, Erkki Vartiainen, Farshad Farzadfar, Gabor Zajkas, Gillian Swan, Guansheng Ma, Hajah Masni Ibrahim, Harri Sinkko, Isabelle Sioen, Jean-Michel Gaspoz, Jillian Odenkirk, Kanitta Bundhamcharoen, Keiu Nelis, Khairul Zarina, Lajos Biro, Lars Johansson, Leanne Riley, Mabel Yap, Manami Inoue, Maria Szabo, Marja-Leena Ovaskainen, Meei-Shyuan Lee, Mei Fen Chan, Melanie Cowan, Mirnalini Kandiah, Ola Kally, Olof Jonsdottir, Pam Palmer, Philippos Orfanos, Renzo Asciak, Robert Templeton, Rokiah Don, Roseyati Yaakub, Rusidah Selamat, Safiah Yusof, Sameer Al-Zenki, Shu-Yi Hung, Sigrid Beer-Borst, Suh Wu, Widjaja Lukito, Wilbur Hadden, Xia Cao, Yi Ma, Yuen Lai, Zaiton Hjdaud, Jennifer Ali, Ron Gravel, Tina Tao, Jacob Lennert Veerman, Mustafa Arici, Demosthenes Panagiotakos, Yanping Li, Gülden Pekcan, Karim Anzid, Anuradha Khadilkar, Veena Ekbote, Irina Kovalskys, Arlappa Nimmathota, Avula Laxmaiah, Balakrishna Nagalla, Brahmam Ginnela, Hemalatha Rajkumar, Indrapal Meshram, Kalpagam Polasa, Licia Iacoviello, Marialaura Bonaccio, Simona Costanzo, Yves Martin-Prevel, Nattinee Jitnarin, Wen-Harn Pan, Yao-Te Hsieh, Sonia Olivares, Gabriela Tejeda, Aida Hadziomeragic, Le Tran Ngoan, Amanda de Moura Souza, Daniel Illescas-Zarate, Inge Huybrechts, Alan de Brauw, Mourad Moursi, Augustin Nawidimbasba Zeba, Maryam Maghroun, Nizal Sarrafzadegan, Noushin Mohammadifard, Lital Keinan-Boker, Rebecca Goldsmith, Tal Shimony, Gudrun B Keding, Shivanand C Mastiholi, Moses Mwangi, Yeri Kombe, Zipporah Bukania, Eman Alissa, Nasser Al-Daghri, Shaun Sabico, Rajesh Jeewon, Martin Gulliford, Tshilenge S Diba, Kyungwon Oh, Sihyun Park, Sungha Yun, Yoonsu Cho, Suad Al-Hooti, Chanthaly Luangphaxay, Daovieng Douangvichit, Latsamy Siengsounthone, Pedro Marques-Vidal, Peter Vollenweider, Constance Rybak, Amy Luke, Nipa Rojroongwasinkul, Noppawan Piaseu, Kalyana Sundram, Jeremy Koster, Donka Baykova, Parvin Abedi, Sandjaja Sandjaja, Fariza Fadzil, Noriklil Bukhary Ismail Bukhary, Pascal Bovet, Yu Chen, Norie Sawada, Shoichiro Tsugane, Lalka Rangelova, Stefka Petrova, Vesselka Duleva, Ward Siamusantu, Lucjan Szponar, Hsing-Yi Chang, Makiko Sekiyama, Khanh Le Nguyen Bao, Sesikeran Boindala, Jalila El Ati, Ivonne Ramirez Silva, Juan Rivera Dommarco, Luz Maria Sanchez-Romero, Simon Barquera, Sonia Rodríguez-Ramírez, Nayu Ikeda, Sahar Zaghloul, Anahita Houshiar-rad, Fatemeh Mohammadi-Nasrabadi, Morteza Abdollahi, Khun-Aik Chuah, Zaleha Abdullah Mahdy, Alison Eldridge, Eric L Ding, Herculina Kruger, Sigrun Henjum, Milton Fabian Suarez-Ortegon, Nawal Al-Hamad, Veronika Janská, Reema Tayyem, Bemnet Tedla, Parvin Mirmiran, Almut Richter, Gert Mensink, Lothar Wieler, Daniel Hoffman, Benoit Salanave, Shashi Chiplonkar, Anne Fernandez, Androniki Naska, Karin De Ridder, Cho-il Kim, Rebecca Kuriyan, Sumathi Swaminathan, Didier Garriguet, Anna Karin Lindroos, Eva Warensjo Lemming, Jessica Petrelius Sipinen, Lotta Moraeus, Saeed Dastgiri, Sirje Vaask, Tilakavati Karupaiah, Fatemeh Vida Zohoori, Alireza Esteghamati, Sina Noshad, Suhad Abumweis, Elizabeth Mwaniki, Simon G Anderson, Justin Chileshe, Sydney Mwanza, Lydia Lera Marques, Samuel Duran Aguero, Mariana Oleas, Luz Posada, Angelica Ochoa, Alan Martin Preston, Khadijah Shamsuddin, Zalilah Mohd Shariff, Hamid Jan Bin Jan Mohamed, Wan Manan, Bee Koon Poh, Pamela Abbott, Mohammadreza Pakseresht, Sangita Sharma, Tor Strand, Ute Alexy, Ute Nöthlings, Indu Waidyatilaka, Ranil Jayawardena, Julie M Long, K Michael Hambidge, Nancy F Krebs, Aminul Haque, Liisa Korkalo, Maijaliisa Erkkola, Riitta Freese, Laila Eleraky, Wolfgang Stuetz, Laufey Steingrimsdottir, Inga Thorsdottir, Ingibjorg Gunnarsdottir, Lluis Serra-Majem, Foong Ming Moy, Corina Aurelia Zugravu, Elizabeth Yakes Jimenez, Linda Adair, Shu Wen Ng, Sheila Skeaff, Regina Fisberg, Carol Henry, Getahun Ersino, Gordon Zello, Alexa Meyer, Ibrahim Elmadfa, Claudette Mitchell, David Balfour, Johanna M Geleijnse, Mark Manary, Laetitia Nikiema, Masoud Mirzaei, Rubina Hakeem

**Affiliations:** 1 Food is Medicine Institute, Friedman School of Nutrition Science and Policy, Tufts University, Boston, MA, USA; 2 Institute of Health Metrics and Evaluation, University of Washington, Seattle, WA, USA; 3 University of Thessaly, Volos, Greece; 4 Department of Medicine, McMaster University, Hamilton, ON, Canada; 5 Population Health Research Institute, Hamilton, ON, Canada; 6 Center for Surgery and Public Health, Brigham and Women’s Hospital Boston, MA, USA; 7 Friedman School of Nutrition Science and Policy, Tufts University, Boston, MA, USA; 8 Research Center on Nutrition and Health, National Institute of Public Health, Cuernavaca, Morelos, Mexico; 9 Tufts University School of Medicine, Boston, MA, USA; 10 Division of Cardiology, Tufts Medical Center, Boston, MA, USA

## Abstract

**Objective:**

To quantify global intakes of sugar sweetened beverages (SSBs) and trends over time among children and adolescents.

**Design:**

Population based study.

**Setting:**

Global Dietary Database.

**Population:**

Children and adolescents aged 3-19 years in 185 countries between 1990 and 2018, jointly stratified at subnational level by age, sex, parental education, and rural or urban residence.

**Results:**

In 2018, mean global SSB intake was 3.6 (standardized serving=248 g (8 oz)) servings/week (1.3 (95% uncertainly interval 1.0 to 1.9) in south Asia to 9.1 (8.3 to 10.1) in Latin America and the Caribbean). SSB intakes were higher in older versus younger children and adolescents, those resident in urban versus rural areas, and those of parents with higher versus lower education. Between 1990 and 2018, mean global SSB intakes increased by 0.68 servings/week (22.9%), with the largest increases in sub-Saharan Africa (2.17 servings/week; 106%). Of 185 countries included in the analysis, 56 (30.3%) had a mean SSB intake of ≥7 servings/week, representing 238 million children and adolescents, or 10.4% of the global population of young people.

**Conclusion:**

This study found that intakes of SSBs among children and adolescents aged 3-19 years in 185 countries increased by 23% from 1990 to 2018, parallel to the rise in prevalence of obesity among this population globally. SSB intakes showed large heterogeneity among children and adolescents worldwide and by age, parental level of education, and urbanicity. This research should help to inform policies to reduce SSB intake among young people, particularly those with larger intakes across all education levels in urban and rural areas in Latin America and the Caribbean, and the growing problem of SSBs for public health in sub-Saharan Africa.

**Figure:**
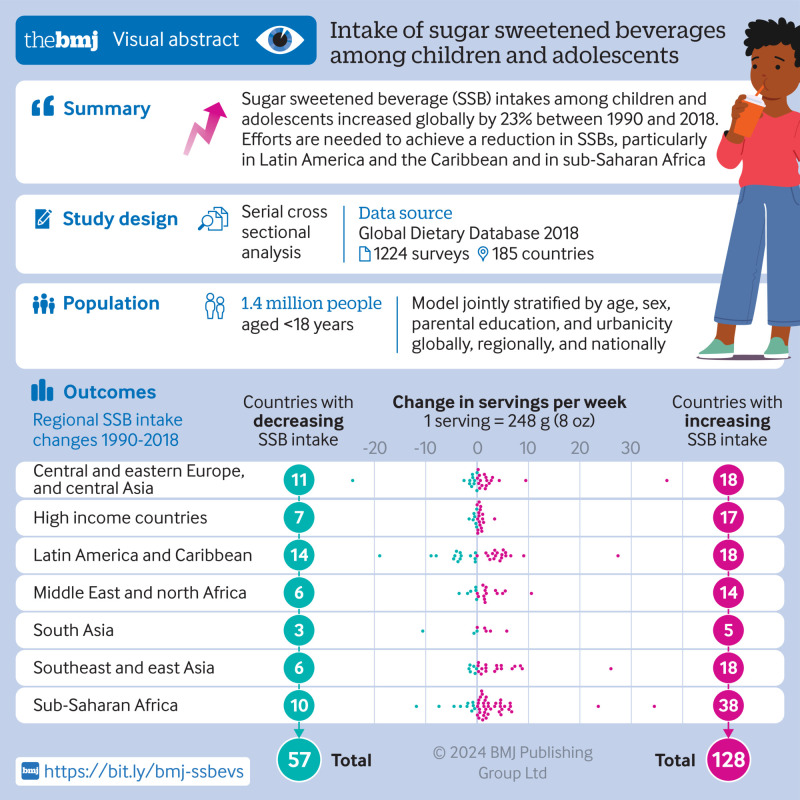


## Introduction

In 2015, obesity was estimated to affect more than 100 million children and adolescents, in line with observed increases in body mass index among this population from 1975 to 2016 in most world regions.^1^ ^43^ Among the main risk factors for obesity, unhealthy diets play a crucial role.^2^ In particular, intake of sugar sweetened beverages (SSBs) has been consistently reported to increase the risk of obesity among children and adolescents.^2^ ^3^ This is especially concerning because obesity in childhood tends to persist into adulthood, increasing the risk of type 2 diabetes, cardiovascular disease, and premature mortality.^4^ Explanations for the increase in intake of SSBs include globalization of markets, transformation of food systems, aggressive marketing strategies directed at children and adolescents, and lack of (or poor) regulatory measures to limit intake.^5^ ^6^ In studies at national and subnational level, policies and strategies such as taxation on sugar sweetened drinks, restrictions on food marketing, regulations for front-of-package labeling, and restrictions at school level have proven to curb the intake of SSBs among children and adolescents.^6^ ^7^ ^8^

Although quantifying the intake of SSBs among children and adolescents is critical to further evaluate the impact of these beverages on disease and the effectiveness of policies to control intake, recent national estimates among young people are unavailable for most countries.^6^ The lack of such data prevents an analysis of the trends in SSB intake over time, as well as the role of key sociodemographic factors such as age, sex, education, and urbanicity to more accurately inform current and future policies. In this study we present SSB intakes among children and adolescents aged 3-19 years at global, regional, and national level and trends over time from 1990 to 2018, jointly stratified at subnational level by age, sex, parental level of education, and area of residence.

## Methods

### Study design

This investigation is based on a serial cross sectional analysis of SSB intakes from the Global Dietary Database 2018 for 185 countries. Details on the methods and standardized data collection protocol are described in detail elsewhere.^9^ ^10^ ^11^ ^12^ ^13^ Compared with the Global Dietary Database 2010, innovations include major expansion of individual level dietary surveys and global coverage up to 2018; inclusion of new data jointly stratified at subnational level by age, sex, education level, and urban or rural residence; and updated modeling methods, covariates, and validation to improve prediction of stratum specific mean intakes and uncertainty. This present analysis focused on children and adolescents aged 3-19 years.

### Data sources

The approach and results of our survey search strategy by dietary factor, time, and region are reported in detail elsewhere.^11^ We performed systematic online searches for individual level dietary surveys in global and regional databases: PubMed, Embase, Web of science, LILACS, African Index Medicus, and the South-east Asia Index Medicus, using search terms “nutrition” *or* “diet” *or* “food habits” *or* “nutrition surveys” *or* “diet surveys” *or* “food habits”[mesh] *or* “diet”[mesh] *or* “nutrition surveys”[mesh] *or* “diet surveys”[mesh] *and* (“country of interest”). Additionally, we identified surveys through extensive personal communications with researchers and government authorities throughout the world, inviting them to be corresponding members of the Global Dietary Database. The search included surveys that collected data on at least one of 54 foods, beverages, nutrients, or dietary indices, including SSBs. A single reviewer screened identified studies by title and abstract, a random subset of articles was screened by a second reviewer to ensure consistency and accuracy, and a third reviewer screened studies to ensure that survey inclusion criteria were met. Surveys were prioritized if they were performed at national or subnational level and used individual level dietary assessments with standardized 24 hour recalls, food frequency questionnaires, or short standardized questionnaires (eg, Demographic Health Survey questionnaires). When national or subnational surveys at individual level were not identified for a country, we searched for individual level surveys from large cohorts, the World Health Organization (WHO) Global Infobase, and the WHO Stepwise Approach to Surveillance database. When individual level dietary surveys were not identified for a particular country, we considered household budget surveys. We excluded surveys focused on special populations (eg, exclusively pregnant or nursing mothers, individuals with a specific disease) or cohorts (eg, specific occupations or dietary patterns). Supplementary methods 1-3, supplementary tables 1-2, and supplementary figure 1 provide additional details on the methods. The final Global Dietary Database model incorporated 1224 dietary surveys from 185 countries, with 89% representative at national or subnational level, thus covering about 99.0% of the global population in 2018. Among these, 450 surveys reported data on SSBs, 85% of which provided individual level data. These 450 originated from 118 countries and surveyed a total of 2.9 million individuals, with 94% being representative at national or subnational level (see supplementary tables 4 and 5). Supplementary data 1 provides details on the characteristics of the survey.

### Data extraction

For each survey, we used standardized methods to extract data on survey characteristics and dietary metrics, units, and mean and standard deviation of intake by age, sex, education level, and urban or rural residence (see supplementary methods 1).^12^ All intakes are reported adjusted to 5439 kilojoules (kJ) daily (1300 kilocalories (kcal) daily) for ages 3-5 years, 7113 kJ/day (1700 kcal/day) for ages 6-10 years, and 8368 kJ/day (2000 kcal/day) for ages 11-19 years. SSBs were defined as any beverages with added sugars and ≥209 kJ (50 kcal) for each 237 g serving, including commercial or homemade beverages, soft drinks, energy drinks, fruit drinks, punch, lemonade, and aguas frescas. This definition excluded 100% fruit and vegetable juices, non-caloric artificially sweetened drinks, and sweetened milk. All included surveys used this definition.

### Data modeling

Our model estimates intakes of SSBs for years for which we have survey data available. To incorporate and deal with differences in data comparability and sampling uncertainty, we used a bayesian model with a nested hierarchical structure (with random effects by country and region) to estimate the mean consumption of SSBs and its statistical uncertainty for each of 264 population strata across 185 countries for 1990, 1995, 2000, 2005, 2010, 2015, and 2018. Our model incorporated seven world regions: central and eastern Europe and central Asia, high income countries, Latin America and the Caribbean, the Middle East and north Africa, south Asia, southeast and east Asia, and sub-Saharan Africa. Our team and others (eg, the Global Burden of Disease study) have previously used this (or similar) classification for world regions, which aims to group nations by general similarities in risk profiles and disease outcomes. Although the current analysis only focuses on children and adolescents aged 3-19 years, the model used all age data to generate the strata predictions. Modeling all age groups jointly allows the use of the full set of available data and covariates to inform estimates, including age patterns, relationships between predictors and SSB intakes, and influence of covariates (eg, dietary assessment methods).

Primary inputs were the survey level quantitative data on SSB intakes (by country, time, age, sex, education level, and urban or rural residence), survey characteristics (dietary assessment method, type of dietary metric), and country-year specific covariates (see supplementary methods 2). The model included overdispersion of survey level variance for surveys that were not nationally representative or not stratified by smaller age groups (≤10 years), sex, education level, or urbanicity. Survey level covariates addressed potential survey bias, and the overdispersion parameter non-sampling variation due to survey level error (from imperfect study design and quality). The model then estimated intakes jointly stratified by age (<1, 1-2, 3-4, 5-9, 10-14, 15-19, 20-24, 25-29, 30-34, 35-39, 40-44, 45-49, 50-54, 55-59, 60-64, 65-69, 70-74, 75-79, 80-84, 85-89, 90-94, ≥95 years), sex, education (≤6 years, >6-12 years, >12 years), and urbanicity (urban, rural). For children and adolescents (age <20 years) the stratification by education refers to parental education.

The uncertainty of each stratum specific estimate was quantified using 4000 Monte Carlo iterations to determine posterior predictive distributions of mean intake jointly by country, year, and sociodemographic subgroup. We computed the median intake and the 95% uncertainty interval (UI) for each stratum as the 50th, 2.5th, and 97.5th percentiles of the 4000 draws, respectively. For model selection and validation, we compared results from fivefold cross validation (randomly omitting 20% of the survey data at the stratum level and using that to evaluate predictive ability, run five times), compared predicted country intakes with survey observed intakes, assessed implausible estimates (see supplementary table 2), and visually assessed global and national mean intakes using heat maps.

A second bayesian model was used to strengthen time trend estimates for dietary factors (including SSBs) with corresponding available date on food or nutrients from the Food and Agriculture Organization’s food balance sheets^14^ or the Global Expanded Nutrient Supply dataset.^15^ No time component was formally included in the model; rather, time was captured by the underlying time variation in the model covariates. This second model incorporated country level intercepts and slopes, along with their correlation estimated across countries. The model is commonly referred to as a varying slopes model structure, and it leverages two dimensional partial pooling between intercepts and slopes to regularize all parameters and minimize the risk of overfitting.^16^ ^17^ The final presented results are a combination of these two bayesian models, as detailed in supplementary methods 3.

### Statistical analysis

Global, regional, national, and within country population subgroup intakes of SSBs and their uncertainty were calculated as population weighted averages using all 4000 posterior predictions for each of the 264 demographic strata in each country-year. Population weights for each year were derived from the United Nations (UN) Population Division,^18^ supplemented with data for education and urban or rural status from Barro and Lee^19^ and the UN.^20^

Intakes were calculated as 248 g (8 oz) servings weekly, or two thirds of a common 355 mL (12 oz) can of a sugar sweetened drink weekly. Absolute changes and percentage changes in consumption between 1990 and 2005, 2005 and 2018, and 1990 and 2018 were calculated at the stratum specific prediction level to account for the full spectrum of uncertainty and standardized to the proportion of individuals within each stratum in 2018 to account for changes in population characteristics over time. Stratum specific predictions were summed to calculate the differences in intake between all children and adolescents aged 3-19 years, high and low parental education (>12 years and ≤6 years, respectively), and urban and rural residence, further stratified by sex, age, parental education, and area of residence, as appropriate.

National intakes of SSBs and trends were assessed by sociodemographic development index, including trends over time between 1990 and 2005, 2005 and 2018, and 1990 and 2018. The sociodemographic development index is a measure of the development of a country or region, ranging from 0 to 1, with 0 representing the minimum level and 1 the maximum level of development of a given nation, and it is based on income per capita, average educational attainment, and fertility rates.^21^ Our UIs are derived from a bayesian model and can be interpreted as at least 95% probability that the true mean is contained within the interval. For comparisons between groups (or over time), if the 95% UI of the difference (or change over time) does not include zero, this can be interpreted as at least 95% probability of a true difference. No hypothesis testing was conducted, as estimation with uncertainty has been recognized as a more informative approach.^22^

### Patient and public involvement

No patients or members of the public were involved in the study as we did not collect data directly from individuals, the funding source did not provide support for direct patient and public involvement, and the study was initiated before patient and public involvement was common. The present analysis used modeled data derived from dietary data that had been previously collected, and we engaged with a diverse set of 320 corresponding members in nations around the world.

## Results

### Global, regional, and national SSB intakes in 2018

**Fig 1:**
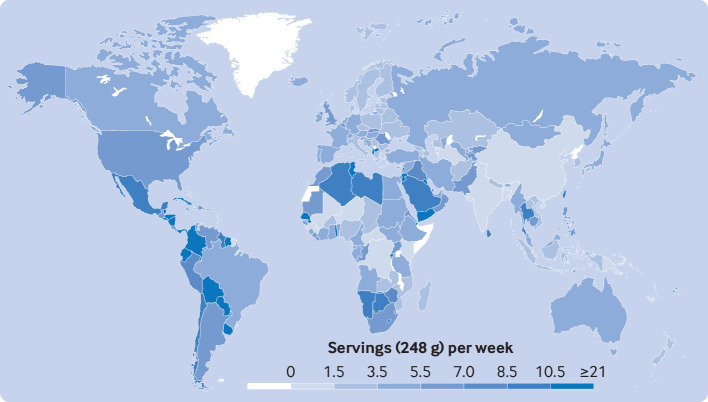
National mean intakes of SSBs (standardized 248 g (8 oz) serving/week for this analysis) in children and adolescents aged 3-19 years across 185 countries in 2018. SSBs were defined as any beverage with added sugars and ≥209 kJ (50 kcal) per 237 g serving, including commercial or homemade beverages, soft drinks, energy drinks, fruit drinks, punch, lemonade, and aguas frescas. This definition excludes 100% fruit and vegetable juices, non-caloric artificially sweetened drinks, and sweetened milk. For this visual representation, values were truncated at 21 servings/week to better reflect the distribution of intakes globally. The figure was created using the rworldmap package (v1.3-6). SSB=sugar sweetened beverage

In 2018, the mean global intake of SSBs among children and adolescents was 3.6 (standardized serving=248 g (8 oz)) servings/week (95% UI 3.3 to 4.0), with wide (sevenfold) variation across world regions, from 1.3 servings/week (1.0 to 1.9) in south Asia to 9.1 (8.3 to 10.1) in Latin America and the Caribbean (table 1). Among the 25 countries with the largest population of children and adolescents worldwide, mean highest intakes were in Mexico (10.1 (9.1 to 11.3)), followed by Uganda (6.9 (4.5 to 10.6)), Pakistan (6.4 (4.3 to 9.7)), South Africa (6.2 (4.7 to 8.1)), and the US (6.2 (5.9 to 6.6)); while the lowest intakes were in India and Bangladesh (0.3 servings/week each) (fig 1, also see supplementary figure 9). Of the 185 countries included in the analysis, 56 (30.3%) had mean SSB intakes of ≥7 servings/week, representing 238 million young people aged 3-19 years, or 10.4% of the global population for this age group.

**Table 1:** Global and regional mean intake of SSBs (248 g (8 oz) serving/week) in children and adolescents aged 3-19 years, by age, sex, parental education, and area of residence across 185 countries in 2018

	Mean (95% UI)							
	Worldwide	Central and eastern Europe and central Asia*	High income countries	Latin America and the Caribbean	Middle East and north Africa	South Asia*	Southeast and east Asia	Sub-Saharan Africa
Overall	3.6 (3.3 to 4.0)	4.0 (3.5 to 4.7)	5.0 (4.8 to 5.2)	9.1 (8.3 to 10.1)	7.3 (6.2 to 8.9)	1.3 (1.0 to 1.9)	2.1 (1.8 to 2.5)	4.2 (3.3 to 5.3)
**Sex**								
Female	3.6 (3.2 to 3.9)	3.8 (3.3 to 4.5)	4.5 (4.3 to 4.8)	8.8 (7.9 to 9.8)	7.3 (6.2 to 9.0)	1.3 (0.9 to 1.9)	2.1 (1.8 to 2.5)	4.1 (3.2 to 5.3)
Male	3.7 (3.4 to 4.1)	4.2 (3.6 to 5.0)	5.4 (5.2 to 5.7)	9.5 (8.5 to 10.5)	7.3 (6.1 to 8.9)	1.4 (1.0 to 1.9)	2.1 (1.8 to 2.5)	4.3 (3.3 to 5.5)
**Age (years)**								
3-4	1.8 (1.6 to 2.1)	1.4 (1.0 to 1.9)	1.9 (1.7 to 2.0)	4.4 (4.0 to 5.0)	3.3 (2.7 to 4.0)	1.7 (1.2 to 2.5)	0.9 (0.7 to 1.2)	1.4 (1.0 to 1.9)
5-9	3.2 (2.9 to 3.6)	3.4 (2.9 to 4.2)	4.0 (3.8 to 4.2)	8.0 (7.2 to 8.9)	6.8 (5.8 to 8.3)	1.4 (1.0 to 2.0)	1.9 (1.6 to 2.2)	3.3 (2.5 to 4.4)
10-14	4.3 (3.9 to 4.7)	5.1 (4.5 to 6.1)	6.0 (5.7 to 6.3)	10.6 (9.6 to 11.8)	9.2 (7.8 to 11.2)	1.2 (0.9 to 1.8)	2.6 (2.2 to 3.0)	5.3 (4.1 to 6.8)
15-19	4.6 (4.2 to 5.1)	5.4 (4.7 to 6.4)	6.7 (6.4 to 7.1)	11.5 (10.4 to 12.7)	9.0 (7.6 to 11.0)	1.1 (0.8 to 1.6)	2.5 (2.2 to 3.0)	6.6 (5.2 to 8.4)
**Parental education level**								
0-6 years (low)	3.2 (2.8 to 3.6)	4.0 (3.1 to 5.2)	5.1 (4.7 to 5.5)	8.3 (7.4 to 9.5)	8.0 (6.5 to 10.3)	1.1 (0.7 to 1.6)	2.0 (1.7 to 2.5)	3.4 (2.6 to 4.5)
>6-12 years (medium)	3.8 (3.4 to 4.2)	4.3 (3.6 to 5.2)	5.2 (4.9 to 5.5)	9.7 (8.6 to 10.9)	7.0 (6.0 to 8.2)	1.1 (0.8 to 1.7)	2.0 (1.8 to 2.4)	5.9 (4.5 to 7.7)
>12 years (high)	4.6 (4.3 to 5.1)	3.8 (3.2 to 4.5)	4.9 (4.6 to 5.1)	9.8 (8.5 to 11.4)	5.7 (4.9 to 6.8)	4.5 (3.0 to 7.1)	2.3 (2.1 to 2.7)	6.3 (4.6 to 8.6)
**Area of residence**								
Rural	2.7 (2.4 to 3.1)	4.4 (3.6 to 5.4)	5.0 (4.8 to 5.2)	8.8 (7.8 to 10.0)	8.3 (6.6 to 11.0)	0.6 (0.5 to 0.9)	2.1 (1.8 to 2.6)	3.6 (2.8 to 4.6)
Urban	4.6 (4.2 to 5.0)	3.8 (3.3 to 4.4)	5.0 (4.7 to 5.3)	9.2 (8.3 to 10.2)	6.8 (5.9 to 7.9)	2.8 (1.9 to 4.3)	2.0 (1.8 to 2.4)	5.2 (3.9 to 6.9)

SSB=sugar sweetened beverage; UI=uncertainty interval.

The standardized serving size used for this analysis was a 248 g (8oz) serving. All intakes are reported adjusted to 5439 kJ/day (1300 kcal/day) for ages 3-5 years, 7113 kJ/day (1700 kcal/day) for ages 6-10 years, and 8368 kJ/day (2000 kcal/day) for ages 11-19 years. Data are based on a bayesian model that incorporated up to 451 individual level dietary surveys, and additional survey level and country level covariates, to estimate dietary consumption levels. Overall SSB intake was defined as any beverage with added sugars and ≥209 kJ (50 kcal) per 237 g serving, including commercial or homemade beverages, soft drinks, energy drinks, fruit drinks, punch, lemonade, and aguas frescas. This definition excluded 100% fruit and vegetable juices, non-caloric artificially sweetened drinks, and sweetened milk.

* In previous Global Dietary Database reports, the region central or eastern Europe and central Asia was referred to as the former Soviet Union, and southeast and east Asia was referred to as Asia.

### SSB intake by sex and age in 2018

**Fig 2:**
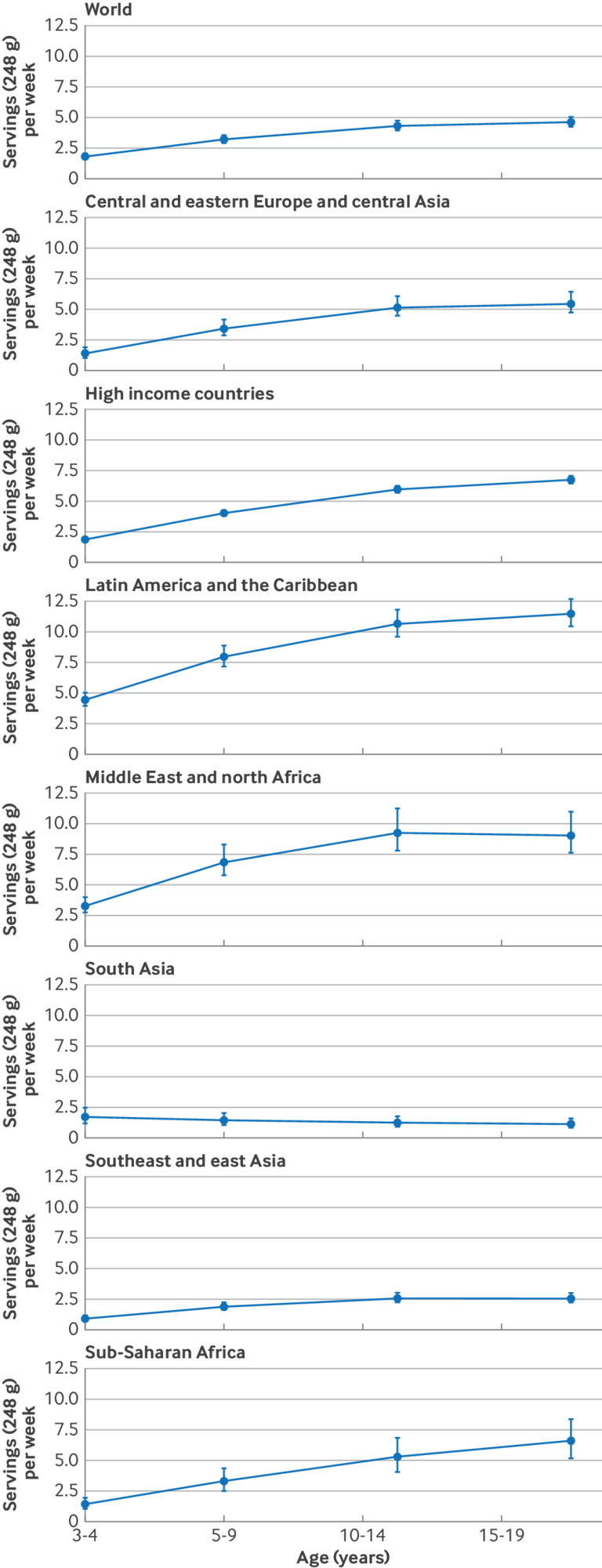
Global and regional intakes of SSBs (standardized 248 g (8 oz) serving/week for this analysis) by age in children and adolescents aged 3-19 years in 2018. SSBs were defined as any beverage with added sugars and ≥209 kJ (50 kcal) per 237 g serving, including commercial or homemade beverages, soft drinks, energy drinks, fruit drinks, punch, lemonade, and aguas frescas. This definition excludes 100% fruit and vegetable juices, non-caloric artificially sweetened drinks, and sweetened milk. The filled circles represent the mean SSBs intake (248 g (8 oz) serving/week) and the error bars the 95% UIs. In previous Global Dietary Database reports, the region central and eastern Europe and central Asia was referred to as the former Soviet Union, and southeast and east Asia was referred to as Asia. SSBs=sugar sweetened beverages; UI=uncertainty interval

Globally, regionally, and nationally, SSB intakes between male and female children and adolescents aged 3-19 years did not differ noticeably, as observed by the 95% UI of the differences including zero (table 1, also see supplementary tables 7 and 8). Intake of SSBs in young people was greater with increasing age globally and regionally, although with varying magnitude of these differences by region (table 1 and fig 2). For instance, intakes of SSBs exceeded 9 servings/week among children aged ≥10 years in Latin America and the Caribbean and in the Middle East and north Africa but were just over 1 serving/week among young people of the same age in south Asia. Regionally, patterns of intake by age were similar between young people (see supplementary figure 2). Considering both age and region, the highest weekly intakes of SSBs were in Latin America and the Caribbean in 15-19 year olds (11.5 servings/week) and lowest in southeast and east Asia in 3-4 year olds (0.9 servings/week) (table 1). Among the 25 most populous countries, the highest intakes of SSBs were in Mexico among 10-14 year olds (11.9 servings/week) and 15-19 year olds (12.8 servings/week) and lowest in Kenya and China among 3-4 year olds (0.2 servings/week each) (supplementary table 6).

### SSB intake by parental education and urbanicity in 2018

**Fig 3:**
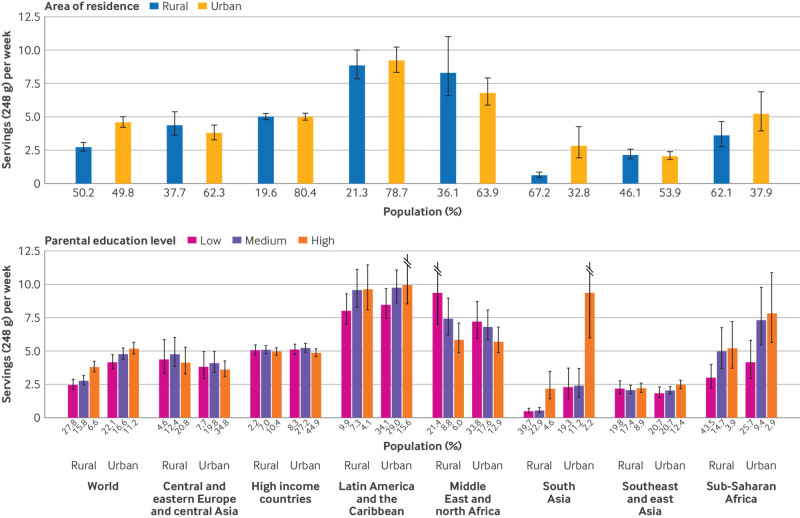
Global and regional mean SSB intakes (standardized 248 g (8 oz) serving/week for this analysis) in children and adolescents aged 3-19 years by area of residence and parental education level in 2018. SSBs were defined as any beverage with added sugars and ≥209 kJ (50 kcal) per 237 g serving, including commercial or homemade beverages, soft drinks, energy drinks, fruit drinks, punch, lemonade, and aguas frescas. This definition excludes 100% fruit and vegetable juices, non-caloric artificially sweetened drinks, and sweetened milk. Error bars represent 95% UIs. Values were truncated at 11.5 servings/week to better reflect the distribution of intakes. Upper 95% UIs above that value are shown with a dashed line. In previous Global Dietary Database reports, the region central and eastern Europe and central Asia was referred to as the former Soviet Union, and southeast and east Asia was referred to as Asia. SSBs=sugar sweetened beverages; UI=uncertainty interval

Intakes of SSBs were greater in children and adolescents from urban areas than those from rural areas (4.6 servings/week (4.2 to 5.0) *v* 2.7 servings/week (2.4 to 3.1); table 1). When parental education and area of residence was assessed jointly, globally the highest intakes of SSBs were among children and adolescents of parents with high education in urban areas (5.15 servings/week (4.76 to 5.64)), representing 11.2% of the global population of children and adolescents (fig 3). Regionally, a similar pattern was observed in Latin America and the Caribbean, south Asia, and sub-Saharan Africa, with the largest intakes of SSBs in children and adolescents of parents with high and medium education in urban and rural areas in Latin America and the Caribbean (≥9 servings/week each), representing 56% of the population of children and adolescents in that region. Intakes of SSBs by area of residence and education were inverted in the Middle East and north Africa, with larger intakes among children and adolescents from rural areas and of parents with lower education, and little variation was observed in other world regions. See supplementary tables 7, 9, and 10, supplementary figures 3 and 4, and supplementary results for further details on SSB intakes by parental education and area of residence.

### Trends in SSB intake during 1990-2005, 2005-18, and 1990-2018

**Fig 4:**
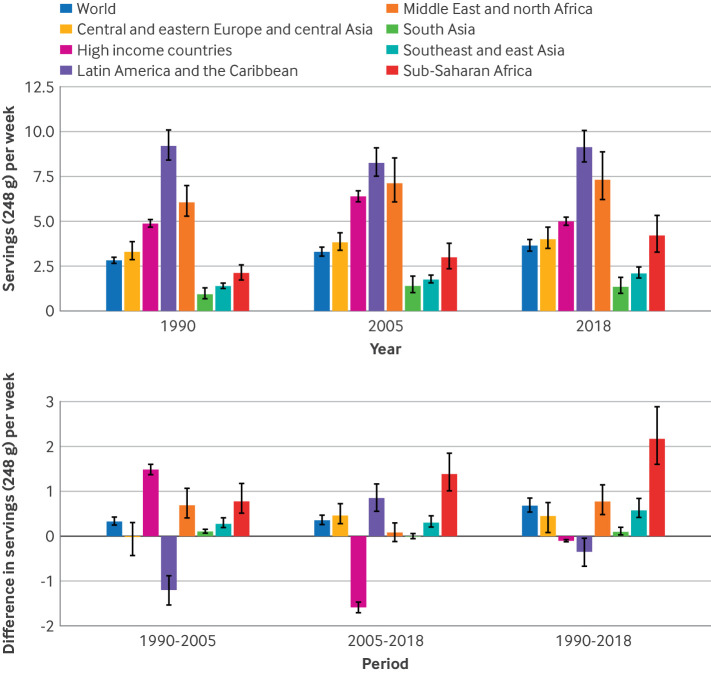
(Top panel) Mean SSB intakes (standardized 248 g (8 oz) serving/week for this analysis) by world region in 1990, 2005, and 2018, and absolute changes from 1990 to 2005, 2005-18, and 1990-2018 in children and adolescents aged 3-19 years. (Bottom panel) Absolute changes in SSB intakes from 1990-2005, 2005-18, and 1990-2018. SSBs were defined as any beverage with added sugars and ≥209 kJ (50 kcal) per 237 g serving, including commercial or homemade beverages, soft drinks, energy drinks, fruit drinks, punch, lemonade, and aguas frescas. This definition excludes 100% fruit and vegetable juices, non-caloric artificially sweetened drinks, and sweetened milk. Error bars represent 95% UIs. In previous Global Dietary Database reports, the region central and eastern Europe and central Asia was referred to as the former Soviet Union, and southeast and east Asia was referred to as Asia. SSBs=sugar sweetened beverages; UI=uncertainty interval

Supplementary tables 11-14 and supplementary figures 5-8 show absolute global, regional, and national intakes of SSBs for 1990 and 2005. Globally, from 1990 to 2018, intakes among children and adolescents increased by 0.68 servings/week (95% UI 0.54 to 0.85; 22.9%) (fig 4, also see supplementary data 2). The magnitude of global increase was similar from 1990 to 2005 (0.33 (0.25 to 0.43); 11.0%) and from 2005 to 2018 (0.35 (0.26 to 0.47); 10.7%). However, regionally, changes did not follow the same global pattern. Between 1990 and 2005, increases in intakes of SSBs were observed in most regions, with the largest increase in high income countries (1.48 (1.37 to 1.60); 29.1%), little change in central and eastern Europe and central Asia and in south Asia, and a decrease in Latin America and the Caribbean (−1.20 (−1.54 to −0.88); −12.7%). More recently, from 2005 to 2018, increases continued in most regions, with the largest in sub-Saharan Africa (1.38 (1.01 to 1.85); 49.2%), except for south Asia where little change was evident and high income countries where intakes decreased (−1.59 (−1.71 to −1.47); −24.1%). In the overall period from 1990 to 2018, the largest regional increase was in sub-Saharan Africa (2.17 (1.60 to 2.88); 106%), with other world regions showing steady, more modest increases over time. Exceptions were high income countries and Latin America and the Caribbean, where intakes increased after 1990 and then decreased close to 1990 levels by 2018. The supplementary results and supplementary table 15 describe regional trends over time by age, sex, parental education, and urbanicity.

Among the 25 most populous countries, the largest increase from 1990 to 2005 was in the US (2.95 (2.73 to 3.17); 43.2%) and the largest decrease was in Brazil (−3.42 (−3.95 to −2.97); −40.6%) (see supplementary data 2 and supplementary figure 9). From 2005 to 2018, the largest increase was in Uganda (4.30 (2.31 to 7.39); 173%), and the largest decrease was in the US (−3.55 (−3.81 to −3.30); −36.4%). Overall, between 1990 and 2018, the largest increased was in Uganda (6.73 (4.38 to 10.39); 5573%) and the largest decrease was in Brazil (−3.29 (−3.79 to −2.86); −39.0%) (see supplementary data 2 and supplementary figure 10). The supplementary results and supplementary tables 16-19 show trends over time within the 25 most populous countries by age, sex, parental education, and urbanicity.

### SSB intakes and trends by sociodemographic development index and obesity

In 1990 and 2005 a positive correlation was evident between national intakes of SSBs and sociodemographic development index, with greater intakes observed in countries with a higher sociodemographic development index (see supplementary figures 11 and 12). However, this correlation was no longer present in 2018 (r=−0.001, P=0.99). Intakes of SSBs and prevalence of obesity were positively correlated in both 1990 (r=0.28, P<0.001) and 2018 (r=0.23, P<0.001) (see supplementary figure 13).

## Discussion

Intakes of SSBs among children and adolescents aged 3-19 years in 185 countries increased by 23% (0.68 servings/week (0.54 to 0.85)) from 1990 to 2018, parallel to the rise in prevalence of obesity among this population globally.^23^ We found a positive correlation between intake of SSBs and prevalence of obesity among children and adolescents in all years. This finding needs particular attention given the incremental economic costs associated with overweight and obesity globally, which are projected to increase from about $2.0tn (£1.6tn; €1.9tn) in 2020 to $18tn by 2060, exceeding 3% of the world’s gross domestic product.^24^ Chronic diet related conditions such as obesity have been recognized as part of a global syndemic along with undernutrition given their interaction and shared underlying societal drivers.^25^ Tackling drivers of obesity and other diet related diseases among children and adolescents is also critical to be better equipped for potential future pandemics, as cardiometabolic conditions such as obesity, diabetes, and hypertension were top drivers of increased risk of hospital admission and death with covid-19.^26^ The increase in intakes of SSBs among children and adolescents corresponded to nearly twice the absolute increase in intake observed among the adult population from 1990 to 2018, for which policies targeting specifically children and adolescents are critical.^13^ Young people are particularly appealing to the food industry as they are easily influenced by food marketing, having an effect on not only their current intakes but also their preferences as they develop into adulthood.^27^ Their susceptibility to marketing, rising trends in obesity, and accelerated increases in intakes of SSBs underline the necessity for interventions such as taxes, regulations on front-of-package labeling, and regulations in the school environment to curb intakes of SSBs.^6^ ^8^ ^27^ ^28^

Changes in intakes of SSBs in children and adolescents from 1990 to 2018 varied substantially by world region. As with the adult population, the largest increase from 1990 to 2018 was in sub-Saharan Africa, emphasizing the need for prompt interventions in this region. Young people in the Middle East and north Africa and in southeast and east Asia showed a more accelerated increase in SSB consumption compared with adults, underlining the importance of policies targeting young people in these regions. The Middle East and north Africa had the second highest intakes of SSBs among children and adolescents in 2018, which differed from our findings among adults, where the Middle East and north Africa occupied third place after sub-Saharan Africa.

Latin America and the Caribbean experienced an overall decrease in intakes of SSBs from 1990 to 2005, which could be attributed to the economic crisis experienced among most of the major economies in the region during this period,^29^ in addition to potential greater health awareness as a result of healthy eating campaigns in several countries in the region.^30^ In contrast, the increases in intakes in this region from 2005 to 2018 may relate to economic recovery, increased marketing campaigns, and industry opposition to public policies to reduce the intake of SSBs.^31^ These findings align with findings in the adult population of this region.^13^ Over the past 30 years, Latin America and the Caribbean has undergone an accelerated transformation in the food systems, resulting in wider availability of unhealthy foods, including SSBs, that could explain the large intakes in this region.^7^ Moreover, the influence of multinational corporations responsible for ultra-processed foods, marketing strategies targeted at young people, lack of (or poor) regulatory measures to limit the intake of SSBs have also been consistently observed in Latin America and other regions with improving economies.^1^ ^6^ ^7^ The use of social media and TV to target advertising at young people has been identified as being especially high in Latin America as well as in the Middle East.^6^ ^27^

High income countries experienced an overall decrease in intakes of SSBs from 2005 to 2018. This might be explained by the increasing scientific and public health attention on the harms of SSBs as well as obesity in these nations during this period, which may have led to increased media and public awareness about the harms to health associated with SSBs, wider formulation, promotion, and availability of non-caloric sweetened beverage substitutes, and, more recently, taxation on SSBs in several of these nations.^32^

The potential role of sociodemographic factors on intakes of SSBs was evidenced by the large variations in intake by parental education and urbanicity, particularly in south Asia and sub-Saharan Africa, evidencing the need to account for these factors in the design of policies and interventions. At national level, the correlation between intake of SSBs and sociodemographic development index changed from positive in 1990 to null in 2018 (see supplementary figure 11), suggesting that the association between the two might be reversing. This is similar to what was observed in adults, where the association between intake of SSBs and sociodemographic index changed from null to negative from 1990 to 2018.^13^ Our new findings show similar directional trends in national and subnational intakes of SSBs among young people compared with adults,^13^ although with generally higher absolute intakes among young people, suggesting nation specific influences on SSB intakes are at least partly shared across the lifespan. Further efforts are needed to incorporate data on other social determinants of health, such as income, access to water, access to healthcare, and race/ethnicity to elucidate additional potential heterogeneities.

### Strengths and limitations of this study

Our study has several strengths. We assessed and reported global, regional, and national estimates of SSB intakes jointly stratified by age, sex, parental education, and urbanicity among children and adolescents. Compared with previous estimates, our current model included a larger number of dietary surveys, additional demographic subgroups, and years of assessment. Our updated bayesian hierarchical model better incorporated survey and country level covariates—and addressed heterogeneity and uncertainty about sampling and modeling.^13^ ^33^ Intakes were estimated from 450 surveys—mostly representative at national and subnational levels and collected at individual level—and represented 87.1% of the world’s population. Other recent estimates for global intakes of SSBs relied mostly on national per capita estimates of food availability (eg, Food and Agriculture Organization food balance sheets) or sales data.^34^ Such estimates can substantially overestimate and underestimate intake compared with individual level data^35^ and are less robust for characterizing differences across population subgroups. Our estimates are informed by dietary data at individual level collected from both 24 hour recalls (24% of surveys), considered the ideal method for assessing nutritional intakes of populations), and food frequency questionnaires (61% of surveys), a validated approach for measuring intakes of SSBs^36^ (see supplementary table 4).

Overall, our findings should be taken as the best currently available, but nonetheless imperfect, estimates of SSB intakes worldwide. Even with systematic searches for all relevant surveys, we identified limited availability of data for several countries (particularly lower income nations) and time periods.^11^ Thus, estimated findings in countries with no primary individual level surveys have higher corresponding uncertainty, informing surveillance needs to assess SSBs nationally and in populations at subnational level. Particularly, we identified limited surveys for south Asia (n=9) and sub-Saharan Africa (n=22), which might have affected the accuracy of our estimates in those regions (see supplementary table 4). This finding emphasizes the critical need for further efforts in data collection and surveillance, particularly in these regions. Categorization by age, parental education, and urbanicity were in groups rather than in more nuanced classifications, balancing the interest in subgroup detail versus the realities required from a global demographic effort of de novo harmonized analyses of individual level dietary data from hundreds of different dietary surveys and corresponding members globally. All types of dietary assessments include some errors, whether from individual level surveys, national food availability estimates, or other sources. Our model’s incorporation of multiple types and sources of dietary assessments provided the best available estimates of global diets, as well as the uncertainty of these estimates. For instance, self-reported data rely on the memory and personal biases of the respondents, thus introducing potential bias from underreporting or overreporting of actual intakes. Furthermore, assumptions relating to standardization of serving sizes, SSB definitions, energy adjustment, and disaggregation at household level, as well as of no interaction between sociodemographic variables in our model, could have impacted our estimates. To minimize these limitations, we used standardized approaches and carefully documented each survey’s methods and standardization processes to maximize transparency.

Our definition and data collection on SSBs excluded 100% fruit juice, sugar sweetened milk, tea, and coffee, given that evidence for health effects of these beverages is inconsistent and does not achieve at least probable evidence for causal harms.^37^ ^38^ These differences may relate to additional nutrients, such as calcium, vitamin D, fats, and protein in milk, caffeine and polyphenols in coffee and tea, and fiber and vitamins in 100% juice; or to differences in rapidity of consumption and drinking patterns. Each of these beverages is generally also excluded in policy and surveillance efforts around SSBs. A recent meta-analysis suggested a modest positive association between 100% fruit juices and body mass index in children (0.03 units higher for each daily serving),^39^ highlighting the need for more research on the health impacts of these and other beverages in children. Sweetened milks are mostly targeted at children and adolescents, and in some regions are mostly consumed by the youngest children.^40^ Given that our SSBs definition excluded sweetened milk, this could partially explain the low intakes observed in our study among the youngest age categories. Future studies should also look into characterizing intakes of sweetened milks, especially in countries such as the US, Australia, Pakistan, and Chile where high intakes among children and adolescents have been reported.^40^ ^41^ Home sweetened teas and coffees were not explicitly excluded from the definition of SSBs at the time of data collection, but tea and coffee were collected as separate variables and thus most likely excluded by data owners from the SSBs category. SSBs were defined as beverages with added sugars and ≥209 kJ (50 kcal) per 237g serving, capturing most of the SSBs during the time period of our investigation that typically contained about 418 kJ (100 kcal) per serving. More recently, some SSBs with slightly less than 10 g of added sugar have entered the market. As these are a relatively recent addition, their exclusion is unlikely to meaningfully alter our findings, but future research should focus on more refined surveillance of SSBs to allow flexibility in beverage group definitions—for example, similar to the data harmonized in our collaboration with the FAO/WHO GIFT food consumption data tool.^42^ Our current definition leveraging product name and caloric content to identify beverages with added sugar across the world ensures consistency in reporting.

### Conclusion

Intakes of SSBs among children and adolescents aged 3-19 years in 185 countries increased by almost a quarter from 1990 to 2018, parallel to the rise in prevalence of obesity among this population globally. Policies and approaches at both a national level and a more targeted level are needed to reduce intakes of SSBs among young people worldwide, highlighting the larger intakes across all education levels in urban and rural areas in Latin America and the Caribbean, and the growing problem of SSBs for public health in sub-Saharan Africa. Our findings are intended to inform current and future policies to curb SSB intakes, adding to the UN’s 2030 Agenda for Sustainable Development for improving health and wellbeing, reducing inequities, responsible consumption, poverty, and access to clean water.

**What is already known in this topic**

The intake of sugar sweetened beverages (SSBs) has been consistently reported to increase the risk of obesity among children and adolescents
This is especially concerning given that obesity in childhood tends to persist into adulthood, increasing the risk of type 2 diabetes, cardiovascular disease, and premature mortality
Quantification of SSB intakes among children and adolescents is therefore critical, yet recent estimates among children and adolescents are unavailable for most nations

**What this study adds**

Intakes of SSBs among children and adolescents aged 3-19 years in 185 countries increased by almost a quarter from 1990 to 2018, parallel to the rise in prevalence of obesity among this population globally
Larger intakes were identified across all education levels in urban and rural areas in Latin America and the Caribbean, along with the growing problem of SSBs for public health in sub-Saharan Africa
Intake of SSBs among children and adolescents showed large heterogeneity by region and population characteristics, informing the need for national and targeted policies and approaches to reduce SSB intake among this population worldwide

## Data Availability

The individual SSB intake estimate distribution data used in this as means and uncertainty (SE) for each strata in the analysis are available freely online at the Global Dietary Database (Download 2018 Final Estimates: https://www.globaldietarydatabase.org/data-download). Global Dietary Database data were utilized in agreement with the database guidelines. Absolute and relative differences by strata and by year presented in this analysis were calculated using the 4000 simulations corresponding to the stratum level intake data derived from the bayesian model. The 4000 simulations files can be made available to researchers upon request. Eligibility criteria for such requests include utilization for non-profit purposes only, for appropriate scientific use based on a robust research plan, and by investigators from an academic institution. If you are interested in requesting access to the data, please submit the following documents: (1) proposed research plan (please download and complete the proposed research plan form: https://www.globaldietarydatabase.org/sites/default/files/manual_upload/research-proposal-template.pdf), (2) data-sharing agreement (please download this form https://www.globaldietarydatabase.org/sites/default/files/manual_upload/tufts-gdd-data-sharing-agreement.docx and complete the highlighted fields, have someone who is authorized to enter your institution into a binding legal agreement with outside institutions sign the document. Note that this agreement does not apply when protected health information or personally identifiable information are shared), (3) email items (1) and (2) info.globaldietarydatabase@tufts.edu. Please use the subject line “GDD Code Access Request.” Once all documents have been received, the Global Dietary Database team will be in contact with you within 2-4 weeks about subsequent steps. Data will be shared as .csv or .xlsx files, using a compressed format when appropriate. Population weights for each strata and year were derived from the United Nations Population Division (https://population.un.org/wpp/), supplemented with data for education and urban or rural status from Barro and Lee (doi:10.3386/w15902) and the United Nations (https://population.un.org/wup/Download/).

## Web extra

Extra material supplied by authors

Supplementary information: Supplementary text 1, methods 1-3, tables 1-19, and figures 1-13

## Notes

Contributors: LLC, RM, and DM conceived the study. FC, PS, JZ, JRS, JEM, VM, LLC, RM, DM curated the data. FC, LLC, RM, and DM were responsible for the methodology. LLC, JRS, VM, and RM collected the data. FC, PS, JZ, JEM, VM, and LLC developed the software. FC, PS, JZ, VM, LLC, RM, and DM validated the data. LLC, SBC, SB, RM, and DM performed the formal analysis. LLC prepared the original draft of the manuscript. LLC, FC, PS, JZ, JRS, JEM, VM, SBC, SB, RM, and DM wrote, reviewed, and edited the manuscript. LLC generated the original figures and tables; SBC, SB, RM, and DM supervised the analysis, manuscript draft, and generation of figures and tables. LLC, RM, and DM acquired funding. They are the guarantors. The corresponding author attests that all listed authors meet authorship criteria and that no others meeting the criteria have been omitted.

Transparency: The lead author (LLC) affirms that the manuscript is an honest, accurate, and transparent account of the study being reported; that no important aspects of the study have been omitted; and that any discrepancies from the study as originally planned (and, if relevant, registered) have been explained.

Dissemination to participants and related patient and public communities: Our research will be disseminated to the scientific community in a scientific conference and scientific publications; to the public through our website and social media; and to funders and interested ministries in various nations through presentations and brief reports.

Provenance and peer review: Not commissioned; externally peer reviewed.

Publisher’s note: Published maps are provided without any warranty of any kind, either express or implied. BMJ remains neutral with regard to jurisdictional claims in published maps.

Editor’s note: The visual abstract was included in this article on 9 August 2024 post-publication.
