# Supplementary material for: Intake of sugar sweetened beverages among children and adolescents in 185 countries between 1990 and 2018: population based study
Source: BMJ. 2024 Aug 7;386:e079234. doi: 10.1136/bmj-2024-079234 (PMC11305379; doi:10.1136/bmj-2024-079234)
Supplement: Supplementary file 1 — Supplementary information: Supplementary text 1, methods 1-3, tables 1-19, and figures 1-13 [file larl079234.ww.pdf]

Supplementary Information | Sugar-sweetened beverage intakes among children and adolescents between 1990 and 2018 in 185 countries

Table of Contents

Supplementary Text 1. Global Dietary Database[11] 4

Supplementary Methods 1. Description of data extraction, assessment, standardization, and analysis. 4

Supplementary Table 1. Definition and units of sugar-sweetened beverages[§]. 11

Supplementary Table 2. Group and individual mean plausibility cutoffs for sugar-sweetened beverages 11

Supplementary Methods 2. Covariates 13

Supplementary Methods 3. GDD Estimation Model 15

Supplementary Figure 1. Flowchart of number of countries with dietary surveys identified, standardized, and included in the Bayesian hierarchical model. 22

Supplementary Table 3. Countries by world region (superregion) in GDD 2018. 23

Supplementary Table 4. Characteristics of global data sources of sugar-sweetened beverage intakes. 24

Supplementary Table 5. Number of dietary surveys for SSB intake globally, regionally, and in the 25 most populous countries by time period. 25

Supplementary Table 6. National mean (95% UI) sugar-sweetened beverage intakes (248 g servings/week) in youth (3-19 years) by sex, age, parental education, and area of residence in the 25 most populous countries in 2018. 26

Supplementary Table 7. Absolute difference in sugar-sweetened beverage intakes (248 g serving/week) in youth (3-19 years) by sex, parental education level, and area of residence globally and regionally across 185 countries in 2018. 27

Supplementary Table 8. Absolute difference in sugar-sweetened beverage intakes (248 g serving/week) in boys vs. girls (3-19 years) in the 25 most populous countries in 2018. 28

Supplementary Figure 2. Global and regional sugar-sweetened beverage intakes (248 g servings/week) by age among youth (3-19 years) girls and boys in 2018. 29

Supplementary Figure 3. Difference in sugar-sweetened beverage intakes (248 g serving/week) between youth (3-19 years) from high vs. low educated parents by age and by area of residence. 30

Supplementary Figure 4. Difference in sugar-sweetened beverage intakes (248 g serving/week) between youth (3-19 years) from urban vs. rural areas by age and by parental education level in 2018. 31

Supplementary Table 9. Absolute difference in sugar-sweetened beverage intakes (248 g serving/week) in youth (3-19 years) of high vs. low educated parents in the 25 most populous countries in 2018. 32

Supplementary Table 10. Absolute difference in sugar-sweetened beverage intakes (248 g serving/week) in urban vs. rural areas in the 25 most populous countries in youth (3-19 years) in 2018 33

Supplementary Table 11. Global and regional mean (95% UI) sugar-sweetened beverage intakes (248 g serving/week) in youth (3-19 years) by age, sex, parental education, and area of residence across 185 countries in 1990. 34

Supplementary Table 12. Global and regional mean (95% UI) sugar-sweetened beverage intakes (248 g serving/week) in youth (3-19 years) by age, sex, parental education, and area of residence across 185 countries in 2005. 35

Supplementary Table 13. National mean (95% UI) sugar-sweetened beverage intakes (248 g servings/week) in youth (3-19 years) by sex, age, parental education, and area of residence in the 25 most populous countries in 1990. 36

Supplementary Table 14. National mean (95% UI) sugar-sweetened beverage intakes (248 g servings/week) in youth (3-19 years) by sex, age, parental education, and area of residence in the 25 most populous countries in 2005. 37

Supplementary Figure 5. National mean sugar-sweetened beverage intakes (248 g servings/week) in youth (3-19 years) across 185 countries in 1990 (a) and 2005 (b). 38

Supplementary Figure 6. Global and regional sugar-sweetened beverage intakes (248 g servings/week) by age in youth (3-19 years) in 1990 and 2005. 39

Supplementary Figure 7. Global and regional sugar-sweetened beverage intakes (248 g servings/week) in youth (3-19 years) by area of residence in 1990 and 2005. 40

Supplementary Figure 8. Global and regional sugar-sweetened beverage intakes (248 g servings/week) in youth (3-19 years) by education and area of residence in 1990 and 2005. 41

Supplementary Figure 9. Mean sugar-sweetened beverage intakes (248 g servings/week) in youth (3-19+ years) in the 25 most populous countries in 2018 and absolute change from 1990 to 2005 and from 2005 to 2018. 42

Supplementary Figure 10. Absolute change in mean sugar-sweetened beverage intakes (248 g servings/week) in youth (3-19 years) in the 25 most populous countries from 1990 to 2018. 43

Supplementary Figure 11. National correlation of sugar-sweetened beverage intake (248 g servings/week) in youth (3-19 years) and socio-demographic development index by world region in 1990 and 2018 for 185 countries. 44

Supplementary Figure 12. National correlation of sugar-sweetened beverage intake (248 g servings/week) in youth (3-19 years) and socio-demographic development index by world region in 2005 for 185 countries. 45

Supplementary Figure 13. National correlation of sugar-sweetened beverage intake (248 g servings/week) in youth (3-19 years) and prevalence of obesity (%) in 1990 and 2018 for 185 countries. 46

Supplementary Table 15. Absolute change in mean sugar-sweetened beverage intakes (248 g servings/week) from 1990-2005, 2005-2018, and 1990-2018 in youth (3-19 years) by sex, age, parental education, and area of residence and by world region across 185 countries. 47

Supplementary Results 48

Supplementary Table 16. Absolute change in mean sugar-sweetened beverage intakes (248 g servings/week) in youth (3-19 years) from 1990-2005, 2005-2018, and 1990-2018 by sex in the 25 most populous countries. 49

Supplementary Table 17. Absolute change in mean sugar-sweetened beverage intakes (248 g servings/week) in youth (3-19 years) from 1990-2005, 2005-2018, and 1990-2018 by age in the 25 most populous countries. 50

Supplementary Table 18. Absolute change in mean sugar-sweetened beverage intakes (248 g servings/week) in youth (3-19 years) from 1990-2005, 2005-2018, and 1990-2018 by parental education level in the 25 most populous countries. 51

Supplementary Table 19. Absolute change in mean sugar-sweetened beverage intakes (248 g servings/week) in youth (3-19 years) from 1990-2005, 2005-2018, and 1990-2018 by area of residence in the 25 most populous countries. 52

Supplementary Text 1. Global Dietary Database[11]

Antonia Trichopoulou[11], Murat Bas[12], Jemal Haidar Ali[13], Tatyana El-Kour[14], Anand Krishnan[15], Puneet Misra[15], Nahla Hwalla[16], Chandrashekar Janakiram[17], Nur Indrawaty Lipoeto[18], Abdulrahman Musaiger[19], Farhad Pourfarzi[20], Iftikhar Alam[21], Celine Termote[22], Anjum Memon[23], Marieke Vossenaar[24], Paramita Mazumdar[25], Ingrid Rached[26], Alicia Rovirosa[27], María Elisa Zapata[27], Roya Kelishadi[28], Tamene Taye Asayehu[29], Francis Oduor[30], Julia Boedecker[30], Lilian Aluso[30], Emanuele Marconi[31], Laura D'Addezio[31], Raffaela Piccinelli[31], Stefania Sette[31], Johana Ortiz-Ulloa[32], J.V. Meenakshi[33], Giuseppe Grosso[34], Anna Waskiewicz[35], Umber S. Khan[36], Kenneth Brown[37], Lene Frost Andersen[38], Anastasia Thanopoulou[39], Reza Malekzadeh[40], Neville Calleja[41], Anca Ioana Nicolau[42], Cornelia Tudorie[42], Marga Ocke[43], Zohreh Etemad[43], Mohannad Al Nsour[44], Lydiah M. Waswa[45], Maryam Hashemian[46], Eha Nurk[47], Joanne Arsenault[48], Patricio Lopez-Jaramillo[49], Abla Mehio Sibai[50], Albertino Damasceno[51], Pulani Lanerolle[52], Carukshi Arambepola[53], Carla Lopes[54], Milton Severo[54], Nuno Lunet[54], Duarte Torres[55], Heli Tapanainen[56], Jaana Lindstrom[56], Suvi Virtanen[56], Cristina Palacios[57], Noel Barengo[57], Eva Roos[58], Irmgard Jordan[59], Charmaine Duante[60], Corazon Cerdena (retired)[60], Imelda Angeles-Agdeppa (retired)[60], Josie Desnacido[60], Mario Capanzana (retired)[61], Anoop Misra[62,125], Ilse Khouw[63], Swee Ai Ng[63], Edna Gamboa Delgado[64], Mauricio T. Caballero[65], Johanna Otero[66], Hae-Jeung Lee[67], Eda Koksal[68], Idris Guessous[69], Carl Lachat[70], Stefaan De Henauw[70], Ali Reza Rahbar[71], Alison Tedstone[71], Annie Ling[71], Beth Hopping[71], Catherine Leclercq[71], Christian Haerpfer[71], Christine Hotz[71], Christos Pitsavos[71], Coline van Oosterhout[71], Debbie Bradshaw[71], Dimitrios Trichopoulos[71], Dorothy Gauci[71], Dulitha Fernando[71], Elzbieta Sygnowska[71], Erkki Vartiainen[71], Farshad Farzadfar[71], Gabor Zajkas[71], Gillian Swan[71], Guansheng Ma[71], Hajah Masni Ibrahim[71], Harri Sinkko[71], Isabelle Sioen[71], Jean-Michel Gaspoz[71], Jillian Odenkirk[71], Kanitta Bundhamcharoen[71], Keiu Nelis[71], Khairul Zarina[71], Lajos Biro[71], Lars Johansson[71], Leanne Riley[71], Mabel Yap[71], Manami Inoue[71], Maria Szabo[71], Marja-Leena Ovaskainen[71], Meei-Shyuan Lee[71], Mei Fen Chan[71], Melanie Cowan[71], Mirnalini Kandiah[71], Ola Kally[71], Olof Jonsdottir[71], Pam Palmer[71], Philippos Orfanos[71], Renzo Asciak[71], Robert Templeton[71], Rokiah Don[71], Roseyati Yaakub[71], Rusidah Selamat[71], Safiah Yusof[71], Sameer Al-Zenki[71], Shu-Yi Hung[71], Sigrid Beer-Borst[71], Suh Wu[71], Widjaja Lukito[71], Wilbur Hadden[71], Xia Cao[71], Yi Ma[71], Yuen Lai[71], Zaiton Hjdaud[71], Jennifer Ali[72], Ron Gravel[72], Tina Tao[72], Jacob Lennert Veerman[73], Mustafa Arici[74], Demosthenes Panagiotakos[75], Yanping Li[76], Gülden Pekcan[77], Karim Anzid[78], Anuradha Khadilkar[79], Veena Ekbote[79], Irina Kovalskys[80], Arlappa Nimmathota[81], Avula Laxmaiah[81], Balakrishna Nagalla[81], Brahmam Ginnela[81], Hemalatha Rajkumar[81], Indrapal Meshram[81], Kalpagam Polasa[81], Licia Iacoviello[82,108], Marialaura Bonaccio[82], Simona Costanzo[82], Yves Martin-Prevel[83], Nattinee Jitnarin[84], Wen-Harn Pan[85], Yao-Te Hsieh[85], Sonia Olivares[86], Gabriela Tejeda[87], Aida Hadziomeragic[88], Le Tran Ngoan[89,154], Amanda de Moura Souza[90], Daniel Illescas-Zarate[91,163], Inge Huybrechts[92], Alan de Brauw[93], Mourad Moursi[93], Augustin Nawidimbasba Zeba[94], Maryam Maghroun[95], Nizal Sarrafzadegan[95], Noushin Mohammadifard[95], Lital Keinan-Boker[96], Rebecca Goldsmith[96], Tal Shimony[96], Gudrun B. Keding[97], Shivanand C. Mastiholi[98], Moses Mwangi[99], Yeri Kombe[99], Zipporah Bukania[99], Eman Alissa[100], Nasser Al-Daghri[101], Shaun Sabico[101], Rajesh Jeewon[102,194], Martin Gulliford[103], Tshilenge S. Diba[104], Kyungwon Oh[105], Sihyun Park[105], Sungha Yun[105], Yoonsu Cho[106], Suad Al-Hooti[107], Chanthaly Luangphaxay[109], Daovieng Douangvichit[109], Latsamy Siengsounthone[109], Pedro Marques-Vidal[110], Peter Vollenweider[110], Constance Rybak[111], Amy Luke[112], Nipa Rojroongwasinkul[113], Noppawan Piaseu[113], Kalyana Sundram[114], Jeremy Koster[115], Donka Baykova[116], Parvin Abedi[117], Sandjaja Sandjaja[118], Fariza Fadzil[119], Noriklil Bukhary Ismail Bukhary[120], Pascal Bovet[121,179], Yu Chen[122], Norie Sawada[123], Shoichiro Tsugane[123], Lalka Rangelova[124], Stefka Petrova[124], Vesselka Duleva[124], Ward Siamusantu[126], Lucjan Szponar[127], Hsing-Yi Chang[128], Makiko Sekiyama[129], Khanh Le Nguyen Bao[130], Sesikeran Boindala[131], Jalila El Ati[132], Ivonne Ramirez Silva[133], Juan Rivera Dommarco[133], Luz Maria Sanchez-Romero[133], Simon Barquera[133], Sonia Rodríguez-Ramírez[133], Nayu Ikeda[134], Sahar Zaghloul[135], Anahita Houshiar-rad[136], Fatemeh Mohammadi-Nasrabadi[136], Morteza Abdollahi[136], Khun-Aik Chuah[137], Zaleha Abdullah Mahdy[137], Alison Eldridge[138], Eric L. Ding[139], Herculina Kruger[140], Sigrun Henjum[141], Milton Fabian Suarez-Ortegon[142], Nawal Al-Hamad[143], Veronika Janská[144], Reema Tayyem[145], Bemnet Tedla[146], Parvin Mirmiran[147], Almut Richter[148], Gert Mensink[148], Lothar Wieler[148], Daniel Hoffman[149], Benoit Salanave[150], Shashi Chiplonkar[151], Anne Fernandez[152], Androniki Naska[153], Karin De Ridder[155], Cho-il Kim[156], Rebecca Kuriyan[157], Sumathi Swaminathan[157], Didier Garriguet[158], Anna Karin Lindroos[159], Eva Warensjo Lemming[159], Jessica Petrelius Sipinen[159], Lotta Moraeus[159], Saeed Dastgiri[160], Sirje Vaask[161], Tilakavati Karupaiah[162], Fatemeh Vida Zohoori[164], Alireza Esteghamati[165], Sina Noshad[165], Suhad Abumweis[166], Elizabeth Mwaniki[167], Simon G. Anderson[168], Justin Chileshe[169], Sydney Mwanza[169], Lydia Lera Marques[170], Samuel Duran Aguero[171], Mariana Oleas[172], Luz Posada[173], Angelica Ochoa[174], Alan Martin Preston[175], Khadijah Shamsuddin[176], Zalilah Mohd Shariff[177], Hamid Jan Bin Jan Mohamed[178], Wan Manan[178], Bee Koon Poh[180], Pamela Abbott[181], Mohammadreza Pakseresht[182], Sangita Sharma[182], Tor Strand[183], Ute Alexy[184], Ute Nöthlings[184], Indu Waidyatilaka[185], Ranil Jayawardena[185], Julie M. Long[186], K. Michael Hambidge (†)[186], Nancy F. Krebs[186], Aminul Haque[187], Liisa

Korkalo[188], Maijaliisa Erkkola[188], Riitta Freese[188], Laila Eleraky[189], Wolfgang Stuetz[189], Laufey Steingrimsdottir[190], Inga Thorsdottir[191], Ingibjorg Gunnarsdottir[191], Lluis Serra-Majem[192], Foong Ming Moy[193], Corina Aurelia Zugravu[195], Elizabeth Yakes Jimenez[196], Linda Adair[197], Shu Wen Ng[197], Sheila Skeaff[198], Regina Fisberg[199], Carol Henry[200], Getahun Ersino[200], Gordon Zello[200], Alexa Meyer[201], Ibrahim Elmadfa[201], Claudette Mitchell[202], David Balfour[202], Johanna M. Geleijnse[203], Mark Manary[204], Laetitia Nikiema[205], Masoud Mirzaei[206], Rubina Hakeem[207]

[11]Academy of Athens, Athens, Greece, [12]Acibadem University, Istanbul, Turkey, [13]Addis Ababa University, Addis Ababa, Ethiopia, [14]Aga Khan Foundation, Geneva, Switzerland, [15]All India Institute of Medical Sciences, New Delhi, India, [16]American University of Beirut, Beirut, Lebanon, [17]Amrita School of Dentistry, Eranakulum, India, [18]Andalas University, Padang, Indonesia, [19]Arab Center for Nutrition, Manama, Bahrain, [20]Ardabil University of Medical Sciences, Ardabil, Iran, [21]Bacha Khan University, Charsadda, Pakistan, [22]Biodiversity International, Nairobi, Kenya, [23]Brighton and Sussex Medical School, Brighton, United Kingdom, [24]Center for Studies of Sensory Impairment, Aging and Metabolism (CeSSIAM), Guatemala City, Guatemala, [25]Centre For Media Studies, New Delhi, India, [26]Centro de Atencion Nutricional Antimano (CANIA), Miami, United States, [27]Centro de Estudios sobre Nutrición Infantil (CESNI), Buenos Aires, Argentina, [28]Child Growth and Development Research Center, Isfahan University of Medical Sciences, Isfahan, Iran, [29]College of Applied Sciences, Department of Food Science and Applied Nutrition, Addis Ababa Science and Technology University, Addis Ababa, Ethiopia, [30]Consultative Group on International Agricultural Research (CGIAR), Montpellier, France, [31]Council for Agricultural Research and Economics, Research Centre for Food and Nutrition, Rome, Italy, [32]Cuenca University, Cuenca, Ecuador, [33]Delhi School of Economics, University of Delhi, Delhi, India, [34]Department of Biomedical and Biotechnological Sciences, University of Catnia, Catania, Italy, [35]Department of CVD Epidemiology, Prevention and Health Promotion, Institute of Cardiology, Warsaw, Poland, [36]Department of Community Health Sciences, Aga Khan University, Karachi, Pakistan, [37]Department of Nutrition and Institute for Global Nutrition, University of California Davis, Davis, United States, [38]Department of Nutrition at the University of Oslo, Oslo, Norway, [39]Diabetes Center, 2nd Department of internal Medicine, Athens University, Athens, Greece, [40]Digestive Disease Research Institute, Tehran University of Medical Sciences, Tehran, Iran, [41]Directorate for Health Information & Research, Pietà, Malta, [42]Dunarea de Jos University of Galati, Galati, Romania, [43]Dutch National Institute for Public Health and the Environment (RIVM), Bilthoven, Netherlands, [44]Eastern Mediterranean Public Health Network (EMPHNET), Amman, Jordan, [45]Egerton University, Njoro, Kenya, [46]Epidemiology and Community Health Branch, National Heart, Lung, and Blood Institute, National Institutes of Health, Bethesada, Maryland, USA, [47]Estonian National Institute for Health Development, Tallinn, Estonia, [48]FHI360, Washington, DC, United States, [49]FOSCAL and UDES, Bucaramanga, Colombia, [50]Faculty of Health Sciences, American University of Beirut, Beirut, Lebanon, [51]Faculty of Medicine, Eduardo Mondlane University, Maputo, Mozambique, [52]Faculty of Medicine, University of Colombo, Colombo, Sri Lanka, [53]Faculty of Medicine, University of Colombo, Sri Lanka, Colombo 5, Sri Lanka, [54]Faculty of Medicine/ Institute of Public Health, University of Porto, Porto, Portugal, [55]Faculty of Nutrition and Food Sciences of University of Porto, Porto, Portugal, [56]Finnish Institute for Health and Welfare, Helsinki, Finland, [57]Florida International University, Miami, United States, [58]Folkhälsan Research Center, Helsinki, Finland, [59]Food Envrionment Consumer Behaviour Lever of the Alliance Bioversity International and CIAT, Nairobi, Kenya, [60]Food and Nutrition Research Institute (DOST-FNRI), Manila, Philippines, [61]Food and Nutrition Research Institute (DOST-FNRI), Taguig City, Philippines, [62]Fortis CDOC Center for Excellence for Diabetes, New Delhi, India, [63]FrieslandCampina, Amersfoort, The Netherlands, [64]Fundacion Cardiovascular de Colombia, Bucaramanga, Colombia, [65]Fundacion INFANT and Consejo Nacional De Investigaciones Cientificas y Tecnicas (CONICET), Ciudad Autonoma de Buenos Aires, Argentina, [66]Fundacion Oftalmologica de Santander (FOSCAL), Floridablanca, Colombia, [67]Gachon University, Seongnam-si, South Korea, [68]Gazi University, Yenimahalle/Ankara, Turkey, [69]Geneva University Hospitals, Geneva, Switzerland, [70]Ghent University, Ghent, Belgium, [71]Global Dietary Database Consortium, Boston, United States, [72]Government of Canada, Statistics Canada, Ottawa, Canada, [73]Griffith University, Gold Coast, Queensland, Australia, [74]Hacettepe University Faculty of Medicine, Ankara, Turkey, [75]Harokopio University, Athens (Kallithea), Greece, [76]Harvard School of Public Health, Cambridge, United States, [77]Hasan Kalyoncu University Dept. Nutrition and Dietetics, Gaziantep, Turkey, [78]Higher Institute of Nursing Professions and Health Techniques, Marrakesh, Morocco, [79]Hirabai Cowasji Jehangir Medical Research Institute, Pune, India, [80]ICCAS (Instituto para la Cooperacion Científica en Ambiente y Salud), Buenos Aires, Argentina, [81]ICMR-National Institute of Nutrition, Hyderabad, India, [82]IRCCS Neuromed, Pozzilli, Italy, [83]Institut de Recherche pour le Developpement, Montpellier, France, [84]Institute for International Investigation, NDRI-USA, New York, United States, [85]Institute of Biomedical Sciences, Academia Sinica, Taipei, Taiwan, [86]Institute of Nutrition and Food Technology (INTA), University of Chile, Santiago, Chile, [87]Institute of Nutrition in Central America and Panama (INCAP), Guatemala City, Guatemala, [88]Institute of Public Health of Federation of Bosnia and

Herzegovina, Sarajevo, Bosnia And Herzegovina, [89]Institute of Research and Development, Duy Tan University, Da Nang City, Viet Nam, [90]Institute of Studies in Public Health, Federal University of Rio de Janeiro (UFRJ), Rio de Janeiro, Brazil, [91]Instituto Nacional de Ciencias Médicas y Nutrición Salvador Zubirán, Mexico City, Mexico, [92]International Agency for Research on Cancer, Lyon, France, [93]International Food Policy Research Institute (IFPRI), Washington, DC, United States, [94]Intitut de Recherche en Sciences de la Sante, Bobo Dioulasso, Burkina Faso, [95]Isfahan Cardiovascular Research Center, Cardiovascular Research Institute, Isfahan University of Medical Sciences, Isfahan, Iran, Isfahan, Iran, [96]Israel Center for Disease Control, Tel-Hashomer, Israel, [97]Justus Liebig University of Giessen, Institute of Nutritional Sciences, Giessen, Germany, [98]KLE Academy of Higher Education and Research (Deemed-to-be-University) Jawaharlal Nehru Medical College, Belagavi, India, [99]Kenya Medical Research Institute, Nairobi, Kenya, [100]King Abdulaziz University, Jeddah, Saudi Arabia, [101]King Saud University, Riyadh, Saudi Arabia, [102]King Saud University, Riyadh , Saudi Arabi, [103]King's College London, London, United Kingdom, [104]Kinshasa School of Public Health, Kinshasa, Democratic Republic of Congo, [105]Korea Disease Control and Prevention Agency (KDCA), Cheongju-si, Korea, [106]Korea University, Seoul, Korea, [107]Kuwait Institute for Scientific Research, Safat, Kuwait, [108]LUM University "Giuseppe Degennaro", Casamassima, Italy, [109]Lao Tropical and Public Health Institute, Vientiane Capital, Lao PDR, [110]Lausanne University Hospital (CHUV) and University of Lausanne, Lausanne, Switzerland, [111]Leibniz Centre for Agricultural Landscape Research, Muncheberg, Germany, [112]Loyola University Chicago, Chicago, United States, [113]Mahidol University, Pathom, Thailand, [114]Malaysian Palm Oil Council (MPOC), Kelana Jaya, Malaysia, [115]Max Planck Institute for Evolutionary Anthropology, Leipzig, Germany, [116]Medical Center Markovs, Sofia, Bulgaria, [117]Menopause Andropause Research Center, Ahvaz Jundishapur University of Medical Sciences, Ahvaz, Iran, [118]Ministry of Health, Jakarta, Indonesia, [119]Ministry of Health, Kuala Lumpur, Malaysia, [120]Ministry of Health, Sungai Besar, Sabak Bernam, Malaysia, [121]Ministry of Health, Victoria, Seychelles, [122]NYU School of Medicine, New York, United States, [123]National Cancer Center Institute for Cancer Control, Tokyo, Japan, [124]National Centre of Public Health and Analyses (NCPHA), Sofia, Bulgaria, [125]National Diabetes, Obesity and Cholesterol Foundation (N-DOC), Chairman, Director, Diabetes Foundation (India), Fortis CDOC Hospital for Diabetes and Allied sciences., New Delhi, India, [126]National Food and Nutrition Commission, Lusaka, Zambia, [127]National Food and Nutrition Institute, Warsaw, Poland, [128]National Health Research Institutes, Zhunan township, Taiwan ROC, [129]National Institute for Environmental Studies, Health and Environmental Risk Division, Tsukuba, Japan, [130]National Institute of Nutrition, Hanoi, Viet Nam, [131]National Institute of Nutrition, Hyderabad, India, [132]National Institute of Nutrition and Food Technology & SURVEN RL, Tunis, Tunisia, [133]National Institute of Public Health (INSP), Cuernavaca, Mexico, [134]National Institutes of Biomedical Innovation, Health and Nutrition, Osaka, Japan, [135]National Nutrition Institute, Cairo, Egypt, [136]National Nutrition and Food Technology Research Institute (NNFTRI): SBMU, Tehran, Iran, [137]National University of Malaysia (UKM), Kuala Lumpur, Malaysia, [138]Nestlé Research, Lausanne, Switzerland, [139]New England Complex Systems Institute, Cambridge, United States, [140]North-West University, Potchefstroom South Africa, Potchefstroom, South Africa, [141]Oslo Metropolitan University (OsloMet), Oslo, Norway, [142]Pontificia Universidad Javeriana Seccional Cali, Cali, Colombia, [143]Public Authority For Food and Nutrition, Sabah Al Salem, Kuwait, [144]Public Health Authority of the Slovak Republic, Bratislava, Slovak Republic, [145]Qatar University and University of Jordan, Doha, Qatar, [146]Queensland University of Technology, Brisbane, Australia, [147]Research Institute for Endocrine Sciences, Shahid Beheshti University of Medical Sciences, Tehran, Iran, [148]Robert Koch Institute, Berlin, Germany, [149]Rutgers University, New Brunswick, United States, [150]Santé publique France, the French Public Health Agency, Saint Maurice, France, [151]Savitribai Phule Pune University (SPPU), Pune, India, [152]School of Medicine, Deakin University, Waurn Ponds, Australia, [153]School of Medicine, National and Kapodistrian University of Athens, Athens, Greece, [154]School of Preventive Medicine and Public Health, Hanoi City, Viet Nam, [155]Sciensano (Belgian Public Health Institute), Brussels, Belgium, [156]Seoul National University, Seoul, Korea, [157]St John's Research Institute, Bangalore, India, [158]Statistics Canada, Ottawa, Canada, [159]Swedish Food Agency, Uppsala, Sweden, [160]Tabriz University of Medical Sciences, Tabriz, Iran, [161]Tallinn University, Tallinn, Estonia, [162]Taylor's University, Selangor, Malaysia, [163]Tecnologico de Monterrey, Escuela de Medicina y Ciencias de la Salud, Mexico City, Mexico, [164]Teesside University, Middlesbrough, United Kingdom, [165]Tehran University of Medical Sciences, Tehran, Iran, [166]The Hashemite University, Az Zarqa, Jordan, [167]The Technical University of Kenya, Nairobi, Kenya, [168]The University of the West Indies, Kingston, Jamaica, [169]Tropical Diseases Research Centre, Ndola, Zambia, [170]Unidad de Nutricion Publica, Macul, Chile, [171]Universidad San Sebastian, Providencia, Chile, [172]Universidad Tecnica del Norte, Ibarra, Ecuador, [173]Universidad de Antioquia, Medellin, Colombia, [174]Universidad de Cuenca, Cuenca, Ecuador, [175]Universidad de Puerto Rico, Medical Sciences Department of Biochemistry, San Juan, Puerto Rico, [176]Universiti Kebangsaan Malaysia Medical Centre, Kuala Lumpur, Malaysia, [177]Universiti Putra Malaysia, Serdang, Malaysia, [178]Universiti Sains Malaysia, Kubang Kerian, Malaysia, [179]University Center for Primary Care and Public Health (Unisanté), Lausanne, Switzerland, [180]University

Kebangsaan Malaysia, Selangor, Malaysia, [181]University of Aberdeen, Aberdeen, United Kingdom, [182]University of Alberta, Edmonton, Canada, [183]University of Bergen, Bergen, Norway, [184]University of Bonn, Department of Nutrition and Food Sciences, Bonn, Germany, [185]University of Colombo, Colombo, Sri Lanka, [186]University of Colorado School of Medicine, Aurora, USA, [187]University of Dhaka, Dhaka, Bangladesh, [188]University of Helsinki, Department of Food and Nutrition, Helsinki, Finland, [189]University of Hohenheim, Stuttgart, Germany, [190]University of Iceland, Reykjavik, Iceland, [191]University of Iceland, Reykjavík, Iceland, [192]University of Las Palmas de Gran Canaria (ULPGC), Canary Islands, Las Palmas, Spain, [193]University of Malaya, Kuala Lumpur, Malaysia, [194]University of Mauritius, Reduit, Mauritius, [195]University of Medicine and Pharmacy Carol Davila, Bucharest, Romania, [196]University of New Mexico Health Sciences Center, Albuquerque, United States, [197]University of North Carolina at Chapel Hill, Chapel Hill, United States, [198]University of Otago, Dunedin, New Zealand, [199]University of Sao Paulo, Sao Paulo, Brazil, [200]University of Saskatchewan, Saskatoon, Canada, Saskatoon, Canada, [201]University of Vienna, Vienna, Austria, [202]University of the Southern Caribbean, Port-of-Spain, Trinidad And Tobago, [203]Wageningen University, Wageningen, Netherlands, [204]Washington University in St. Louis, St. Louis, United States, [205]World Health Organization (WHO), Geneva, Switzerland, [206]Yazd Cardiovascular Research Centre, Shahid Sadoughi University of Medical Sciences, Yazd, Iran, [207]Ziauddin University

Supplementary Methods 1. Description of data extraction, assessment, standardization, and analysis.

Data extraction

The sociodemographic characteristics were used as reported by each survey, and comprehensive details on whether these were self-reported or measured in any other way are unavailable. Data were assessed for extraction errors and for plausibility using standardized algorithms, and survey quality by evaluating evidence for selection bias, sample representativeness, response rate, and validity of diet assessment method. Measurement comparability across surveys was maximized by using a standardized data analysis approach including averaging all days of dietary assessment to quantify mean individual-level intakes; using harmonized dietary factor definitions and units of measure across surveys; and adjusting for total energy using age-specific energy intakes to reduce measurement error and account for differences in body size, metabolic efficiency, and physical activity, as further explained below.

Data assessment

Data received from corresponding members or from publicly available surveys were checked to confirm survey-level characteristics; dietary intake variables were categorized into GDD dietary factors; necessary unit and format conversions were noted. Data-owners or survey directors were contacted extensively to resolve questions about data quality, categorization, or assessment methods to ensure accuracy and completeness of data prior to analysis.

Preliminary data checks

Biostatisticians generated survey description files for each survey including survey characteristics, variable lists, and summary statistics for categorical and continuous variables. Research assistants used these description files to assess inclusion of survey level information and demographic variables. Discrepancies between author-reported characteristics and those ultimately included in the dataset were noted for further discussion with the data-owner.

Categorization of variables into GDD dietary factors

Research assistants matched reported dietary data to GDD dietary factors. This involved categorizing foods, nutrients, mixed dishes, and regional items into the matched GDD dietary factor, noting cases where variables represented less than the optimal GDD definition. Unit conversions were included as necessary to transform variables into the optimal GDD units.

Categorization of variables into GDD dietary factors – Food Frequency Questionnaires

To transform food and beverage data reported from Food Frequency Questionnaires into optimal GDD units, most often grams per day, all categorical variables were standardized into single daily serving units. When ranges of frequencies were provided, the mean of each range was utilized to represent each frequency category. Variables reported in times per week were divided by 7 to calculate the average daily servings. Variables reported in times per month were divided by 30.42 (the average number of days in a month) to calculate the average daily servings. In cases where the upper range was open-ended (e.g., "5 or more times per week"), the ranges of the other frequency categories were used to calculate an upper limit. Servings per day were then converted into grams per day by multiplying the number of servings by the author-reported serving sizes or by the GDD standard serving sizes.

Communication with data owners and creation of data key

Any questions regarding the data, including those about region-specific diets (e.g., disaggregation of mixed dishes, classification of regional items), survey-level characteristics, and serving sizes for foods and beverages, were communicated to the data owner. After all questions regarding the data were answered, research assistants generated a data key outlining all available variables of interest, including demographic and dietary variables. Categorizations of dietary variables were turned into STATA code for clear identification and research assistants flagged the quality assurance checks.

Converting household data to individual data

Household-level data were transformed into individual-level data using the Adult Male Equivalent (AME) method. The AME method estimates individual-level intakes by assigning each household member a reference AME based on their age and sex. Household members' reference AMEs are summed to find total household AME. Each individual's reference AME is then divided by the total household AME to find individual-level AME. This individual-level AME represents the proportion each individual contributes to the overall household AME. This individual level proportion is multiplied by the household consumption of each food item to estimate individual-level intake.

Data aggregation

Using preliminary checking documents provided by research assistants, biostatisticians converted individual-level data into aggregated outputs for each dietary factor stratified by the available demographic variables. Stata version 12 was used to convert all demographic and dietary variables from raw data files to a single data file containing only relevant variables. Missing observations were excluded from the dataset and all variables were recoded to match the GDD demographic and dietary variable coding scheme. Data were then aggregated into demographic strata by age, sex, residence, education, and pregnancy/lactating status. We used education as a measure of socioeconomic status, as based on expert consultation this was felt to be a more stable and comparable measure than other variables which can be reported with significant error and underreporting in different countries (e.g., household income). In addition to the single, final data file, supporting files were generated including a summary report, minimum/maximum values for each dietary factor, and group level means, standard deviations, and percentiles of intake. All output files were stored in each survey's specific folder on the Tufts GDD Box, accessible to all members of the research team.

Energy adjustment

We extracted both raw and energy-adjusted data when available. If energy adjustment of individual-level data had not been completed by the data owner, biostatisticians completed energy adjustment at the aggregation stage to age-specific levels using the residual method. Insufficient data were available to globally estimate differences in energy intakes by factors other than age. This approach was considered the "gold standard." We adjusted for total energy intake to mitigate the effects of measurement error in data collection, account for differences in energy requirements related to body size, metabolic efficiency, and physical activity, and facilitate comparisons between surveys, age groups, and sexes.

Total daily energy values by age:

- 0-0.9 years = 700 kcal
- 1-1.9 years = 1,000 kcal
- 2-5 years = 1,300 kcal
- 6-10 years = 1,700 kcal
- 11-74 years = 2,000 kcal
- 75+ years = 1,700 kcal

Child and older adult-specific daily energy values were selected using dietary recommendations and mean population ranges from the USA, United Kingdom, and India.

Energy adjustment corrections

We initially asked that all data be shared both unadjusted and energy-adjusted to 2,000 kcal, regardless of age category, but retrospectively changed this decision to reflect the age-specific levels. When possible, energy adjustment using the residual method was repeated to reflect these changes. In some cases, this approach was not possible, and thus alternative approaches for energy adjustment correction were taken.

Energy adjustment correction of aggregate ("stratum-level") data
In some cases, data were provided or accessed at the stratum level (i.e., age group, sex, education level, etc.). In these cases, energy adjustment correction depended on whether 2,000 kcal/day-adjusted values had previously been provided by the data owner. If energy-adjusted data had been provided, a simple ratio of the age-specific level to 2,000 kcal was applied post-hoc to convert the value to the correct energy level. If stratum-level data were only provided in an unadjusted format but with corresponding total energy intake, intake was adjusted to the age-specific energy level using the energy density method, in which a simple ratio of reported calorie intake to age-specific level was applied to the unadjusted value. If stratum-level data were provided in age groups which traversed more than one level of age-specific energy intake, a weighted mean daily energy intake was calculated. This weighted mean daily energy level was then used to adjust intake using the ratio readjustment method. If only unadjusted intake was available, the energy density method was used.

Energy adjustment of data without adjusted values or total energy intake
In limited cases, individual-level data were not initially energy-adjusted or provided with mean caloric intake data, precluding the use of the gold standard and ratio readjustment methods. In these instances, daily per capita energy availability data from Food and Agriculture Organization (FAO) Food Balance Sheets (FBS) were used to inform stratum-level caloric intake. In short, country-year-specific FBS energy data were adjusted using coefficients derived from a multivariate linear regression of GDD input data, FBS data, and both regional and survey-level covariates. Adjusted FBS energy was then corrected to the prescribed energy level by applying a factor of the energy level's proportion of 2,000 kcal. Unadjusted food and nutrient intake values were then adjusted with this corrected energy intake via the energy density method.

Quality control

Data integrity and quality were assessed at each step during survey collection, processing, harmonization, and analyses. Duplicate reviews were performed of recorded survey characteristics, demographic variables, dietary definition classifications, and unit conversions. To assess for outliers and validity (errors) in reported intakes, plausibility thresholds were defined for each dietary factor, both at the individual level and stratum (e.g., group mean) level, based on dietary reference intakes, tolerable upper limits, toxicity ranges, and existing regional data on mean intakes in populations. Any value identified as potentially implausible was reviewed for extraction errors, followed by direct correspondence with the corresponding member or public survey data owners, to detect and correct potential errors. Data remaining implausible after such steps were excluded from final datasets. Results for each dietary factor were further graphed and visually inspected by country, age, sex, dietary assessment method, representativeness, and time, reviewing survey result plausibility and consistency within and across countries.

Data finalization

After data has been finalized for inclusion, it was stored within the Access database, which houses information on all surveys, corresponding authors, and survey checking statuses. Aggregated data was collated by dietary factor and prepared for input into the GDD prediction model.

Protocol for converting FFQ frequency data into GDD servings

1. Step 1- Standardize the categorical frequency variables to a single daily serving unit
    a. If a range of frequencies is given, take the mean ("Avg") of the range
    b. If the frequency is presented in times/week, divide by 7 (for days in a week)
    c. If the frequency is presented in times/month, divide by 30·42 (average days in a month)
        i. *Note:* If the category is presented as days/week instead of times/week, assume one serving per day and treat as times/week
        ii. Example A) 5-7 days/week = (6 days/week) / (7days/week) = 0·857 servings/day
        iii. Example B) 1-3 times/month = (2 times/month) / (30.42 days/month) = 0·066 servings/day
    d. If the upper range is open ended, use the range of the other frequency categories in the survey to create an upper limit and then take the average of that range.
        i. Example: "5 or more times per week" where next lowest level is 2-4 times per week. Assume a range of 5-7 times per week, take the average (6 times per week)/(7 days/week) = 0·857 servings/day
2. Step 2- Convert servings to grams
    a. If available, survey-specific serving sizes were used for conversions.
    b. If survey-specific serving sizes are not available, ask the data owner for usual, country-specific serving sizes.
    c. If data owner does not provide country-specific serving sizes, utilize country-specific serving sizes identified from national agencies (e.g., USDA).
    d. If no country-specific serving sizes are identified, use the GDD standard serving size conversions.

Common categories of intake and their servings per day conversions

- Never
    - Calculation: 0; Daily Serving: 0
- Occasional-Few times/year
    - Calculation: Should capture the range of values between never and the next highest choice based on the data set.
    - Serving: Depends on next level categorization.
- Less than once a month (1-11 times per year)
    - Calculation: 1+11=12/2=6 Avg servings/year; 6/12 months=0.5 servings/month; 0.5/30.42 days
    - Daily serving: 0.0164
- 1-3 times/month
    - Calculation: 1+3=4/2=2 Avg servings/month; 2/30.42 days
    - Daily serving: 0.066
- 1/ week
    - Calculation: 1 servings/7days
    - Daily serving: 0.143
- 2-4 days/week
    - Calculation: 2+4=6/2=3 Avg servings/week; 3servings/7days
    - Daily serving: 0.429
- 5-6 days/week
    - Calculation: 5+6=11/2= 5.5 Avg servings/week; 5.5/7days
    - Daily serving: 0.786
- 5-7 days/week

- Calculation: 5+7=12/2=6 Avg servings/week; 6/7days
    - Daily serving: 0.857
- 1/day
    - Calculation: NA; Daily serving: 1
- 2-3/day
    - Calculation: 2+3=5/2= Avg 2.5 servings
    - Daily serving: 2.5
- 4-5/day
    - Calculation: 4+5=9/2= Avg 4.5 servings
    - Daily serving: 4.5

Common weight conversions

1 Kilogram = 1000 grams
1 Ounce *Cannot use for fluid ounces = 28 grams
1 Pound = 454 grams

Standard serving sizes for sugar-sweetened beverages

- Variable code: V15
- Variable name: Sugar-sweetened beverages
- Reference serving sizes: 2003-06 US NHANES (median) = 368 g per serving
- "Usual" average serving sizes (g/serving): Adults and children/adolescents older than 2 years = 248g; 12-24 months = 130g; 6 to 11 months = 84g

Supplementary Table 1. Definition and units of sugar-sweetened beverages[§].

Dietary factor	Unit	Preferred definition	Alternative definition
Sugar-sweetened beverages	g/day	Total sugar-sweetened beverage intake, defined as any beverage with added sugars having ≥50 kcal per 8 oz (236.5 g) serving, including commercial or homemade beverages, soft drinks, energy drinks, fruit drinks, punch, lemonade, and frescas. This definition excludes 100% fruit and vegetable juices and non-caloric artificially-sweetened drinks.	Optimal (our condition), including fruits and vegetable juices.

[§] These definitions were used for extracting/requesting SSB intake data.

Supplementary Table 2. Group and individual mean plausibility cutoffs for sugar-sweetened beverages

Group or individual	Age group	Serving size	Mean minimum serving/day	Mean maximum serving/day	Mean min g/day	Mean max g/day
Group	Children/ Adolescents	248 grams	≤ 0	6	≤ 0	1488

	Adults	248 grams	≤ 0	4	≤ 0	992
Individual	Children/ Adolescents	248 grams	≤ 0	15	≤ 0	3720
	Adults	248 grams	≤ 0	15	≤ 0	3720

Supplementary Methods 2. Covariates

Covariate identification

We identified country- and time-specific covariate data from various sources to further inform our model estimates. These data supplement our individual-level dietary intake data, particularly in countries for which these inputs are limited. We consulted experts and conducted comprehensive searches of publicly available databases to identify >800 covariates. We prioritized approximately 400 covariates for testing:

Data source*:	Year(s)
• UN FAO food balance sheets	1980 - 2018
• Harvard Global Expanded Nutrient Supply (GENuS)	1980 - 2011
• Principal component analysis of FAO and GENuS data	2013
• Euromonitor fat and oils sales data	1998 - 2018
• World Bank Gross Domestic Product (GDP)	1980 - 2018
• World Bank unemployment rate	1980 - 2015
• World Bank Gini coefficient	1980 - 2015
• World Bank poverty rate	1980 - 2015
• Barro Lee education	1980 - 2010
• World Bank precipitation	1982 - 2014
• CIA Factbook latitude	N/A
• CIA Factbook land area	N/A
• CIA Factbook coastline ratio	N/A

*Percentage of country-years imputed for each covariate source: GDP=3%; unemployment rate=7%; food balance sheets=11%; fat and oil sales data=11%; GENuS=26%; Gini coefficient and poverty rate=28%; education=87%.

We conducted principal component analysis (PCA) using the 'princomp' function in R separately for: 1) 23 grouped FAO food balance sheet (FBS) foods, beverages, and energy, 2) 142 GeNUS foods, and beverages, and 3) 19 GeNUS nutrients and energy. The first four components from each PCA were considered for covariate testing.

Covariate imputation and truncation

If covariate data were missing for some (but not all) years of a given country, we used linear interpolation to fill in those years. Covariate data sources that ended before 2018 were imputed using a moving average of the three most recent values to obtain values for all covariates through the year 2018. Region-level means were assigned to countries for which entire covariates were missing. To assess validity of the imputations, we imputed non-missing values with the same model and visually compared observed vs. imputed values via scatter plots.

The GDD prediction model operates on the natural log scale (except for dietary factors measured as proportions), including the covariate data. To minimize a strong effect of very small values for covariates with a broad range of values on the log scale having an outsized influence on modeled estimates, we truncated covariate data on the non-transformed scale using the following rules:

For covariates with a 95th percentile value

1. > 3.5: Truncate values < 0.5 to 0.5
2. ≥ 1 and ≤ 3.5: Truncate values < than 0.1 to 0.1
3. < 1: No truncation

Covariate testing

For each dietary factor, we calculated the correlations between covariates and original survey-level stratified mean dietary intakes, and we selected up to 10 covariates for model inclusion, favoring those with the highest correlations, a mix of food/nutrients and other covariates, and sensible links to the dietary factor.

Each of the covariates identified in the correlation stage (maximum 10 covariates) and the four PCA components were then included in a stepwise regression to test for inclusion in GDD models, with an entry point p-value of <0.299 and an exit point of >0.30 during the stepwise process. These stepwise regressions resulted in three nested versions of the GDD model per diet factor:

1. Base model: Closest diet factor proxy from FAO or GENuS (1-2 covariates per model)
2. Restricted model: All covariates with $p<0.1$ from the results of the stepwise regression plus base model covariate(s).
3. Inclusive model: All covariates from the results of the stepwise regression plus base model covariate(s).

For each dietary factor, five-fold cross-validation was used to compare model fit for the three versions of the GDD model. Data were split into five partitions at the survey level: four partitions making up the training dataset, and the remaining segment as the testing data. The models were fit to the training set, and resulting outputs were compared to testing set to assess model fit via calculating expected log predictive density (ELPD).[1] This was repeated five times so that each partition was used once as the training set, and the results were summed across repetitions.

Final model selection and included covariates for sugar-sweetened beverages

Sugar-sweetened beverages:
- Model: Base model (model 1)
- Covariates: FAO sugar and sweeteners

Supplementary Methods 3. GDD Estimation Model

1 Data Overview

Survey data were collected and collated from Corresponding Members and publicly available sources from across the globe as newly analyzed summary data (using harmonized definition, joint strata according to the defined sociodemographics, and energy adjustment) or provided as individual-level data that were analyzed by the GDD using the same methods. Thus, the analysis may be considered as de novo harmonized analyses of individual-level data, pooled in a hierarchical Bayesian model which used a predefined prior and a likelihood function derived from the available data, as well as covariates, to generate a posterior distribution of the mean intakes. Mean intakes were estimated from each survey by subgroup (age group, sex, urbanicity, education level), which we used as our primary inputs for the model. These data have an inherently nested hierarchical structure which makes a multilevel approach to modeling data appealing. The hierarchical structure of the data we assumed was as follows: countries were nested in world regions (superregions), which are nested in the globe.
Our model uses the following seven world regions:

- CEECA (Central/Eastern Europe and Central Asia)*
- HIC (High-Income Countries)
- LAC (Latin America and Caribbean)
- MENA (Middle East and North Africa)
- SA (South Asia)
- SEEA (Southeast and East Asia)*
- SSA (Sub-Saharan Africa)

*In prior GDD reports, the region Central/ Eastern Europe and Central Asia was called the Former Soviet Union, and Southeast and East Asia was called Asia.

Additionally, a host of country-year level data was collected for potential use as predictor variables. A list of these variables, and their sources, can be found below. Note that surveys are rarely fully stratified by all four subgroups of interest. The model uses the most granular stratifications available as input. The availability and level of stratification has been described elsewhere. The uncertainty of the input data was accounted for in the likelihood function, and it was used to characterize the distribution of the observed data.

2 Model description

The GDD estimation model aimed to estimate mean intake of 53 dietary factors in 185 countries by, country/year/age/sex/urbanicity/education, by synthesizing survey mean intake data from sources of varying quality. For the estimation of SSB intakes, our model used 451 surveys, mostly nationally or subnationally representative, collected at the individual-level, and representing 87.1% of the world's population. The Bayesian multilevel framework has some advantageous properties that are appealing for our purposes. Namely,

- "Shrinkage" of parameter estimates towards an overall mean. For example, mean estimates for data sparse countries are pulled towards the region mean, allowing for more reasonable estimates for countries with potentially unreliable data.
- Intuitive framework for predicting means (with uncertainty bounds) for countries with no available data.
- Ability to include prior knowledge about intake through priors.
- Allows for model flexibility and complexity often not granted in similar frequentist approaches due to difficulty in optimization.

Fundamentally, our model is a Bayesian model on the log-means of intake with a nested hierarchical structure (it clusters countries within world regions and world regions within the globe), assuming exchangeability between countries and between world regions. Using this structure allowed us to borrow strength across units, a concept commonly known as "partial pooling". In partial pooling, each country's mean estimate borrows from the other countries' data within the region, resulting in shrinkage of the country mean estimate towards the region mean. The less informative the data were for a particular country, the more pooling there is. To this structure, we added sex,

urban/rural area of residence, education, and non-linear age effects (also within a nested hierarchical structure), survey and country-level covariates through 2018, and overdispersion on study-level variance to account for non-sampling variation. It borrows heavily from models presented by Finucane et al.[3] and Flaxman et al.[4] For dietary factors that were measured as proportions of energy intake, we use *-log(log(y))* as the link function instead of *log(y)*.

Below we provide a full mathematical description of the model, with detailed descriptions for each component, but first, we present some notation:

Subscript notation:

- h: age/sex/educ/urbanicity group
- i: study
- j: country
- k: world regions

Superscript notation:

- c: country
- s: world regions
- g: globe

2.1 The model

$$f(y_{h,i}) \sim N(a_j + b_{1,j} sex_{h,i} + b_{2,j} u_{h,i} + b_{3,j} educ_{h,i} + \gamma_j(z_{h,i}) + X_i\beta,\ SE^2_{h,i} + \tau^2_i)$$

Where,

$f(y)$	$\Leftarrow -log(-log(y))$ for dietary factors measured as proportions, $log(y)$ otherwise
$y_{h,i}$	$\Leftarrow$ mean intake level for stratum h in study i
a_j	$\Leftarrow$ country-specific intercept, for country j
$b_{1,j}$	$\Leftarrow$ country-specific difference between females and males, for country j
$sex_{h,i}$	$\Leftarrow$ variable indicating whether the $y_{h,i}$ corresponds to an all-male group (0), all female group (1), or mixed (0.5), for stratum h in study i
$b_{2,k}$	$\Leftarrow$ region-specific difference between urban and rural, for region k
$u_{h,i}$	$\Leftarrow$ variable indicating whether the $y_{h,i}$ corresponds to an all-rural group (0), all urban group (1),or mixed (% urban), for stratum h in study i
$b_{3,k}$	$\Leftarrow$ region-specific education effect, for region k
$educ_{h,i}$	$\Leftarrow$ two variables indicating whether $y_{h,i}$ corresponds to low education (defined to be 6 years or less of schooling if mixed), proportion of low education, and high education for stratum h in study i
γ_k	$\Leftarrow$ non-linear age-trend, for region k
$z_{h,i}$	$\Leftarrow$ midpoint age, for stratum h in study i
$X_i\beta$	$\Leftarrow$ study + country level covariate effects
$SE^2_{h,i}$	$\Leftarrow$ standard error of $f(y_{h,i})$ (estimated via delta method), for stratum h in study i
τ^2_i	$\Leftarrow$ overdispersion parameter, for study i

2.2 Intercept, sex differences, education differences, and urban/rural differences

We fit a multi-level model with 3 levels (countries nested in world regions nested in the globe) for intercepts and sex differences, and 2 levels (world regions nested in the globe) for age pattern, education differences, and urban/rural differences. Mathematically, this can be described as follows:

$$a_j = a^c_j + a^s_{k[j]} + a^g$$
$$b_{1j} = b^c_{1j} + b^s_{1k[j]} + b^g_1$$
$$b_{2j} = b^s_{2k[j]} + b^g_2$$
$$b_{3j} = b^s_{3k[j]} + b^g_3$$

$$a_j^c \sim N(0, \kappa_a^c), b_{1j}^c \sim N(0, \kappa_{1b}^c),$$
$$a_k^s \sim N(0, \kappa_a^s), b_{1k}^s \sim N(0, \kappa_{1b}^s), b_{2k}^s \sim N(0, \kappa_{2b}^s), b_{3k}^s \sim N(0, \kappa_{3b}^s)$$

where,

a_j ⇐ country-specific intercept, for country *j*
a_j^c ⇐ country-specific random effects, for country *j*
$a_{k[j]}^s$ ⇐ world region-specific random effects, for country *j* in world region *k*
a^g ⇐ global intercept
$b_{1,j}$ ⇐ country-specific sex effect, for country *j*
$b_{1,j}^c$ ⇐ country-specific sex random effects, for country *j*
$b_{1,k[j]}^s$ ⇐ world region-specific sex random effects, for country *j* in world region *k*
b_1^g ⇐ global sex effect
$b_{2,j}$ ⇐ country-specific urban effect, for country *j*
$b_{2,j}^c$ ⇐ country-specific urban random effects, for country *j*
$b_{2,k[j]}^s$ ⇐ world region-specific urban random effects, for country *j* in world region *k*
b_2^g ⇐ global urban effect
$b_{3,j}$ ⇐ country-specific education effect, for country *j*
$b_{3,j}^c$ ⇐ country-specific education random effects, for country *j*
$b_{3,k[j]}^s$ ⇐ world region-specific education random effects, for country *j* in world region *k*
b_3^g ⇐ global education effect
κ_a^c ⇐ between-country variance, at the country level
κ_{1b}^c ⇐ between-country sex variance, at the country level
κ_a^s ⇐ between-world region variance, at the country level
κ_{1b}^s ⇐ between-world region sex variance, at the country level
κ_{2b}^s ⇐ between-world region urban variance, at the country level
κ_{3b}^s ⇐ between-world region education variance, at the country level

Note that $b_{3,j}$ is a vector of two parameters, one for the difference between low and middle education and one for the difference between high and middle education. Also note that the model assumes between-country variance was the same across all world regions. Likewise, education, urban/rural differences and age patters are assumed to be the same for countries within a world region.
Weakly informative priors are used for the hyper-parameters: half-Normal (0, 0.5) for the κ parameters, for a^g, b_1^g, b_2^g, and b_3^g, a prior of N(0, 0.35) is used. We selected weakly informative priors to allow the survey data and covariates to have the largest influence on the posterior distribution. Input data are standardized to the standard normal scale to ensure priors are sensible for all dietary factors and to increase computational stability. In addition, thin-tailed priors like the normal distributions we use, tend to lead to more computational stability when running the model particularly when running complex models in STAN, as we do.

2.3. Covariate effects

There were two survey-level covariate effects included in the model to explain potential bias from a survey: survey type and food definition. There were four main types of diet surveys included as covariates in the model: short-term recalls (single or multiple); food frequency questionnaires (FFQs); household budget/intake surveys; and DHS (Demographic Health Survey) questionnaires. Only the recall is considered the "gold standard" with regards to estimating the mean unbiasedly. Likewise, not all surveys use the optimal definition for a dietary factor. For example, in the case of fruits, most surveys define fruits as "all fruits". However, some surveys will only measure a sub-optimal metric, such as fruits and fruit juices combined. Currently, we combine all sub-optimal metrics into one category. Of note, this was not the case for sugar-sweetened beverages (SSBs), as all surveys report using the optimal definition (i.e., "total sugar-sweetened beverage intake, defined as any beverage with added sugars having ≥50 kcal per 8 oz (236.5g) serving, including commercial or homemade beverages, soft drinks, energy drinks, fruit drinks, punch, lemonade, and frescas; excluding 100% fruit and vegetable juices and non-caloric artificially-sweetened drinks").We also included country-year specific

predictors in the model (e.g., food availability FAO food balance sheets or Global Expanded Nutrient Supply (GENuS) model). The model assumed the relationship of these covariates to $f(y_i)$ was linear, and that the relationships were independent of location (not super-region dependent, or country dependent) and year. Mathematically, this portion of the model can be described as follows:

$$X_i\beta = X_i^{survey\ characteristics}\ \beta_s + X_{j[i]}^{country-year\ predictions}\ \beta_c$$

where,

$X_i\beta$ ⇐ study + country level covariate effects

$X_i^{survey\ characteristics}\beta_s$ ⇐ study characteristics for study i + world region level covariate effects

$X_{j[i]}^{country-year\ predictions}\ \beta_c$ ⇐ country-year predictors for study i in country j + country level covariate effects

For survey level-covariates, we used a weakly prior of N(0, 0.35), reflecting our a priori belief that average survey bias exceeding 50% would be extremely rare. The prior for βc parameters depended on the dietary factor. For many dietary factors, we only used 1 or 2 country-level covariates, all from FAO. For these variables, we had a very strong prior belief that they should be strongly correlated with our outcome variable of interest (e.g., log(sugar and sweeteners availability from FAO) should be strongly positively correlated with log(SSBs intake)). In these cases, we used a highly informative prior of N(1, 0.1). For other dietary factors, either no such variable existed, or other country-year level predictors were also included and do not warrant such a high degree of certainty in a strong relationship. In these cases, we used a much weaker prior of N(0, 0.5). Thin-tailed priors were utilized to enhance model stability and convergence, particularly in STAN.

2.4 Age trend

Age is treated as a continuous variable in the model, and every survey strata is assigned the midpoint of the age group for modeling purposes. For many surveys, intake was not linearly associated with age. We modelled age using restricted cubic splines with 4 knots at $k1$, $k2$, $k3$, $k4$, corresponding to ages 5, 20, 50 and 65, respectively, after standardization:

$$\gamma_{j[i]}(z_h) = \gamma_{1j[i]}z_h + \gamma_{2j[i]}S_1 + \gamma_{3j[i]}S_2$$

where,

$$S_1 = (z_h - k_1)^3 - \frac{k_4 - k_1}{k_4 - k_3}(z_h - k_3)^3 + \frac{k_3 - k_1}{k_4 - k_3}(z_h - k_4)^3$$

$$S_2 = (z_h - k_2)^3 - \frac{k_4 - k_2}{k_4 - k_3}(z_h - k_3)^3 + \frac{k_3 - k_2}{k_4 - k_3}(z_h - k_4)^3$$

As with the urban and education effect parameters, we used 2 levels of hierarchy for the age-trend:

$$\gamma_{1j[i]} = \gamma_{1k[j]}^s + \gamma_1^g$$
$$\gamma_{2j[i]} = \gamma_{2k[j]}^s + \gamma_2^g$$
$$\gamma_{3j[i]} = \gamma_{3k[j]}^s + \gamma_3^g$$

$$\gamma_{1k}^s \sim N(0, \kappa_{1\gamma}^s), \gamma_{2k}^s \sim N(0, \kappa_{2\gamma}^s), \gamma_{3k}^s \sim N(0, \kappa_{3\gamma}^s)$$

Weakly informative priors are used for the hyper-parameters: half-Normal (0, 0.5) for the κ parameters and Normal (0, 0.35) for γ^g parameters.

2.5 Overdispersion

An additional variance component was added to each study to allow the model to account for non-sampling variation due to survey-level error (from imperfect study design and quality). This additional variance component

was modeled in such a way to reflect our expectation that surveys that are less likely to represent the true mean (but not necessarily biased) were more variable. Sources of this non-sampling variation accounted for included surveys not being nationally representative, surveys not being stratified by sex, urban/rural or education, and surveys that used large age groupings (greater than10 years). We also added an additional constraint to ensure local surveys were considered more variable than regional surveys.

Thus,

$$\tau_i^2 = exp(\ \phi_{intercept} + \ \phi_{regional} I\left(X_i^{rep} = regional\right) + \ \phi_{local} I\left(X_i^{rep} = local\right) + \phi_{AgeRange} I\left(X_i^{AgeRange} > 10\right) + \phi_{sex} I(X_i^{sex} = both) + \phi_{urban/rural} I((X_i^{urban/rural} = both)\ or\ (X_i^{educ} = all))$$

with the constraints $\phi^2_{regional} < \ \phi^2_{local}$, and all $\phi > 0$ except $\phi_{intercept}$. We use a prior of Normal (-2.5, 1) for $\phi_{intercept}$ to reflect our a priori belief than an "ideal" survey that is both fully stratified and nationally representative should have minimal overdispersion. For all other ϕ parameters, we used a prior of Normal (0, 0.5).

3 Computation

We fit each model using STAN[5,6] through Rstan,[7] using the No-U-turn sampler (NUTS),[8] a variant of Hamiltonian Monte Carlo.[9] We use 4 chains of 2000 iterations each, treating the first 1000 iterations of each chain as warm up, for a total of 4000 Monte Carlo iterations to define our posterior distributions.

4 Estimates

The model described above was ultimately used to provide posterior distributions of mean intake for each dietary factor by country-year and subgroup. Note that the model specified $g\left(\psi_{h,i[j]}\right)$ of subgroup h in survey i from country j as a linear combination of model parameters and survey-year-subgroup specific information:

$$a_j + \ b_{1j} sex_{h,i} + \ b_{2s} u_{h,i} + \ b_{3s} educ_{h,i} + \ \gamma_s(z_{h,i}) + \ X_i\beta$$

where we have posterior distributions for model parameters a_j, b_j, b_{2s}, b_{3s}, γ_s, and β. To obtain a posterior distribution for subgroup h in country j, we calculate for each draw of our posterior distribution:

$$\mu_{hj} = \ g^{-1}(a_j + \ b_{1j} sex_{h,j} + \ b_{2s} u_{h,j} + \ b_{3s} educ_{h,j} + \ \ \gamma_s(z_{h,j}) + \ X_j^{country-year\ predictors} \beta_c)$$

Because we are interested in country-specific means, we did not use survey specific parameters in our estimates. For countries with no survey data, we did not have posterior distribution for a_j. To get the posterior distributions for these countries in such a way that accounts for the variation of mean intake between countries within a region, we report the distribution of $a_j^*$and b_{1j}^*, where $a_j^* \ \sim N(a_{k[j]}, \kappa_a^c)$ and $b_{1j}^* \ \sim N\left(b_{1k[j]}, \kappa_{1b}^c\right)$. Here, $a_{k[j]}$ and $b_{1k[j]}$ are world region-level intercepts and sex effects corresponding to country j, and κ_a^c and κ_{1b}^c are the between-country variances for intercept and sex effects, respectively. In other words, each posterior draw for the world region-level parameter and it's corresponding between-country variance parameter generates a unique normal distribution for that draw, and we take a one sample draw from each of these distributions to generate the posterior distribution of that parameter for an unknown country in that region. Note that the uncertainty around the world region level parameter and between country variance propagate into the posterior distribution for the mean. For some dietary factors, there were entire world regions with no data. For those world regions, posterior distributions for b_{2s}, b_{3s}, and γ_s were obtained in a similar way, generating a normal distribution for each draw from the global level parameter and between region variance parameter and sampling from that. For a_j and b_{1j}, we needed to account for between-world region variance and the between-country variance. Therefore, taking the intercept as an example, for each posterior draw, we sampled from $N(a^g, \kappa_a^c + \ \kappa_a^s)$. Note that this is equivalent to drawing a sample region mean from $N(a^g, \kappa_a^s)$ then using that sample as mean and κ_a^c as variance to form a normal distribution to sample country mean from. Importantly, the uncertainty inherent in this approach is captured and reported as 95% UIs of each mean; and nations with more missing data or other uncertainty had larger UIs.

5 Varying slopes modeling structure

- Our extensive work to identify surveys and model intakes led to recognition and the finding that, for certain dietary factors, the available global data and model were insufficient to accurately model differences in intakes jointly stratified by country, age, sex, education level, and urban/rural status while also modeling differences in intakes over time.
- For countries without multiple comparable dietary surveys over time (the great majority of global nations), trends over time are largely determined by the strength of the relationship between the best available covariates (often variables from FAO food balance sheets or associated GENuS variables) and the raw survey data. For certain dietary factors, this relationship was sufficiently robust to allow modeling of all joint demographic strata and time trends. By reviewing extensive time trends plots for individual dietary factors and nations, dietary factors with a model beta coefficient>=0.4 with their corresponding FAO/GENuS covariate were identified as having a reasonable statistical relationship to capture both all demographic strata differences and time trends. For others (FAO/GENuS beta coefficient<0.4), time trends were modeled using a second, separate Bayesian model.
- This second Bayesian model assessed the country-specific associations over time of the survey data for each dietary factor with its corresponding FAO/GENuS covariate. The model incorporated country-level intercepts and slopes, along with their correlation that is estimated across countries, to provide more sensitivity and specificity for estimating country-specific associations from dietary data to generate country-specific time trends. Input data were the same stratified survey data as for the GDD Core model and including dietary assessment method as a covariate. This time component model did not separately estimate differences by age, sex, education, or urban/rural status, but focused on the relationships with FAO/GENuS over time. In sensitivity analyses, age and sex were included as main effects (not varying by country or region) but were found to not qualitatively alter the parameter estimates for the relationship of a country's dietary intake data with its FAO/GENuS data. Thus, including these demographics did not largely affect the time-varying estimates. This model is commonly referred as a varying slopes model structure and leverages two-dimensional partial pooling between intercepts and slopes to regularize all parameters and minimize overfitting risk[10-12]. Estimates with the varying slopes model take into account a country-specific intercept and slope when the country has dietary factor data and use the global intercept and slope for countries where data are not available. Time effects were predicted separately for each year including 1990, 1995, 2000, 2005, 2010, 2015, and 2018.
- For each country and dietary factor, the country-specific time-trend central estimates from the varying slopes models were used to generate a country-year specific adjustment scaling factor, one for each year of 1990, 1995, 2000, 2005, 2010, 2015, and 2018, compared to the reference of one of these years as determined by the median year of that country's survey data (or 2005 if no country data). This scaling factor, determined by taking the ratio of the predicted dietary intake for that year as compared to the reference year, was multiplied by the country-year posterior estimates from the fully stratified, Core GDD model to determine a time-adjusted final estimate for each stratum.
- To be conservative, this varying slopes adjustment (scaling factor) was only used for dietary factors and countries meeting all of the following criteria: at the model level, (a) FAO/GENuS beta coefficient<0.4 in the Core GDD model; and (b) availability of a closely corresponding FAO/GENuS covariate (e.g., dietary survey vitamin A intake vs. GENuS vitamin A); and at the country-level, (c) identification of a positive relationship (coefficient or slope) between the national survey data and FAO/GENuS covariate in the varying slopes model; and (d) to minimize implausible results at the country level, no more than a 3-fold difference between the ratio of the country's range of predicted intake between 1990-2018 divided by the ratio of the country's range of FAO/GENuS values over that same time period.
- Among 53 evaluated dietary factors in the GDD, 29 were modeled and incorporated time adjustment using this Bayesian varying slopes model. The other dietary factors were not because (in order of criteria applied) 11 did not have any closely corresponding FAO/GENuS variable (e.g., dietary iodine), 8 had an FAO/GENuS beta coefficient in Core GDD Core Model of at least 0.4, and 4 were unable to complete sampling for the varying-slopes model (i.e., the MCMC chains did not finalize, independent of parameterization). One additional dietary factor, vitamin B9, with a borderline FAO/GENuS beta (0.34) was also not further scaled based on adequate qualitative characteristics of the observed time trends in the GDD Core Model.

A measurement error, varying slopes model that accounts for dietary assessment method, using standardized log-intakes for all dietary factors except those reported in percent energy:

$Y_{obs,i} \sim Normal(Y_{true,i}\, , DE_{SE,i})$ [distribution for observed intake, Y_{obs}, including measurement error associated with the stratum estimate, $DE_{SE,i}$]

$Y_{true,i} \sim Normal(\mu_i\, , \sigma)$ [distribution for true strata intake Y]

$$\mu_i = \alpha_{country[i]} + \beta_{country[i]} * FAO + M_{method[i]}$$

[linear equation for the average intake; Each country receives its own intercept and slope while also accounting for dietary assessment method]

$$\begin{bmatrix} \alpha_{country} \\ \beta_{country} \end{bmatrix} \sim MVNormal\left(\begin{bmatrix} \alpha \\ \beta \end{bmatrix}, S\right)$$

[population of varying effects]

$$S = \begin{pmatrix} \sigma_\alpha & 0 \\ 0 & \sigma_\beta \end{pmatrix} R \begin{pmatrix} \sigma_\alpha & 0 \\ 0 & \sigma_\beta \end{pmatrix}$$

[construct covariance matrix]

With hyperpriors that define the adaptive varying effects and effects for dietary assessment:

$\alpha \sim Normal(0\, , 1)$ [prior for average intercept]

$\beta \sim Normal(1\, , 0.11)$ [prior for average slope]

$M[method] \sim Normal(0\, , 0.2)$ [prior for method effect]

$\sigma \sim Halfnormal\ (0\, ,0.5)$ [prior for stddev within countries]

$\sigma_\alpha \sim Halfnormal\ (0\, ,0.5)$ [prior for stddev among intercepts]

$\sigma_\beta \sim Halfnormal\ (0\, ,0.5)$ [prior for stddev among slopes]

$R \sim LKJcorr\ (2)$ [prior for correlation matrix]

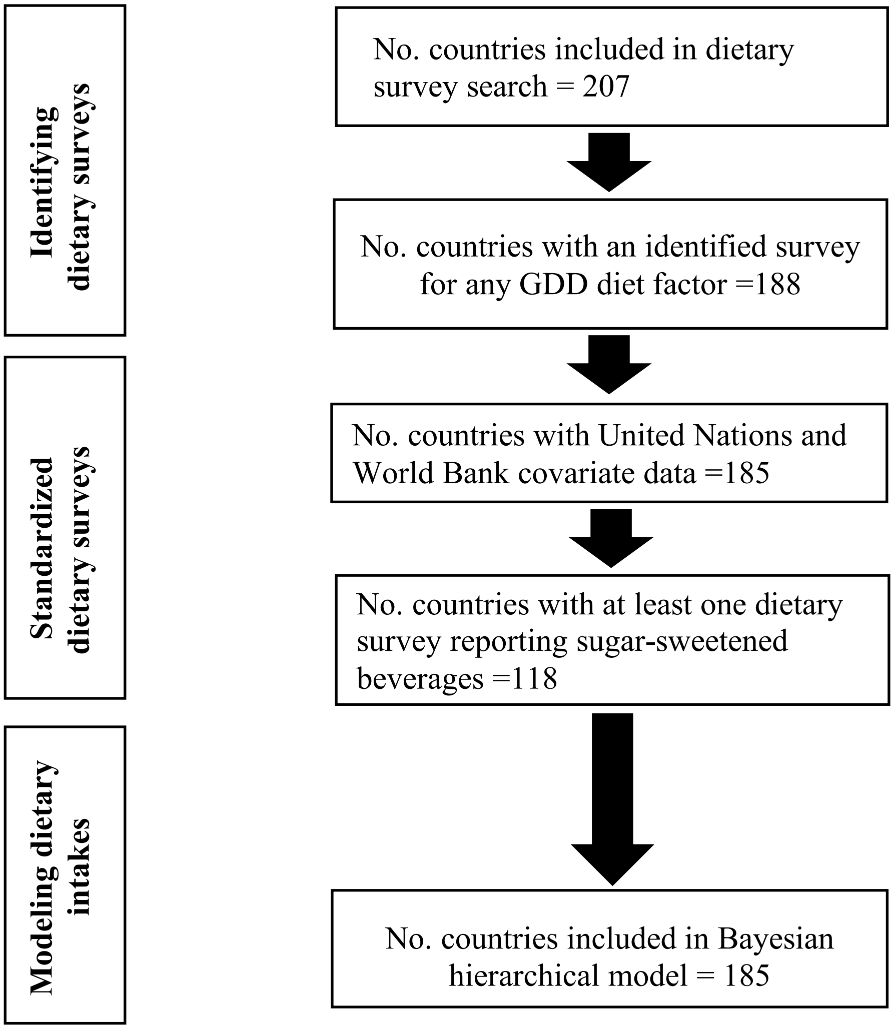

Supplementary Figure 1. Flowchart of number of countries with dietary surveys identified, standardized, and included in the Bayesian hierarchical model. Any GDD diet factor: fruit; non-starchy vegetables; potatoes; other starchy vegetables; beans/legumes; nuts/seeds; refined grains; whole grains; processed meats; unprocessed red meats; seafood; eggs; cheese; yogurt; sugar- sweetened beverages; fruit juices; coffee; tea; reduced fat milk; whole fat milk; total milk; energy; carbohydrate, total protein; animal protein; plant protein; saturated fat; monounsaturated fat; omega-6 fat; seafood omega-3 fat; plant omega-3 fat; dietary cholesterol; dietary fiber; added sugar; calcium; sodium; iodine; iron; magnesium; potassium; selenium; vitamin A with supplement; vitamin A without supplement; vitamin B1; vitamin B2; vitamin B3; vitamin B6; vitamin B9; vitamin C; vitamin D; vitamin E or zinc.

Supplementary Table 3. Countries by world region (superregion) in GDD 2018.

Central/Eastern Europe and Central Asia[†]		High-Income Countries	Latin America and Caribbean		Middle East and North Africa	South Asia[§]	Southeast and East Asia	Sub-Saharan Africa	
n=29		*n=24*	*n=32*		*n=20*	*n=8*	*n=24*	*n=48*	
Albania	Slovenia	Australia	Antigua and Barbuda	Panama	Algeria	Afghanistan	Brunei	Angola	Liberia
Armenia	Tajikistan	Austria	Argentina	Paraguay	Bahrain	Bangladesh	Cambodia	Benin	Madagascar
Azerbaijan	Turkmenistan	Belgium	Bahamas, The	Peru	Egypt, Arab Rep.	Bhutan	China	Botswana	Malawi
Belarus	Ukraine	Canada	Barbados	St. Lucia	Iran, Islamic Rep.	India	Fiji	Burkina Faso	Mali
Bosnia and Herzegovina	Uzbekistan	Cyprus	Belize	St. Vincent and the Grenadines	Iraq	Maldives	Indonesia	Burundi	Mauritania
Bulgaria		Denmark	Bolivia	Suriname	Israel	Nepal	Japan	Cameroon	Mauritius
Croatia		Finland	Brazil	Trinidad and Tobago	Jordan	Pakistan	Kiribati	Cape Verde	Mozambique
Czech Republic		France	Chile	Uruguay	Kuwait	Sri Lanka	Korea, Rep.	Central African Republic	Namibia
Estonia		Germany	Colombia	Venezuela	Lebanon		Lao PDR	Chad	Niger
Georgia		Greece	Costa Rica		Libya		Malaysia	Comoros	Nigeria
Hungary		Iceland	Cuba		Morocco		Marshall Islands	Congo, Dem. Rep.	Rwanda
Kazakhstan		Ireland	Dominica		Oman		Micronesia, Fed. Sts.	Congo, Rep.	Sao Tome and Principe
Kyrgyz Republic		Italy	Dominican Republic		Palestine		Myanmar	Cote d'Ivoire	Senegal
Latvia		Luxembourg	Ecuador		Qatar		Papua New Guinea	Djibouti	Seychelles
Lithuania		Malta	El Salvador		Saudi Arabia		Philippines	Equatorial Guinea	Sierra Leone
Macedonia, FYR		Netherlands	Grenada		Syrian Arab Republic		Samoa	Eritrea	South Africa
Moldova		New Zealand	Guatemala		Tunisia		Singapore	Ethiopia (excludes Eritrea)	South Sudan
Mongolia		Norway	Guyana		Turkey		Solomon Islands	Gabon	Sudan
Montenegro		Portugal	Haiti		United Arab Emirates		Taiwan	Gambia, The	Swaziland
Poland		Spain	Honduras		Yemen, Rep.		Thailand	Ghana	Tanzania
Romania		Sweden	Jamaica				Timor-Leste	Guinea	Togo
Russian Federation		Switzerland	Mexico				Tonga	Guinea-Bissau	Uganda
Serbia		United Kingdom	Nicaragua				Vanuatu	Kenya	Zambia
Slovak Republic		United States					Vietnam	Lesotho	Zimbabwe

[†]Referred to as Former Soviet Union in previous Global Dietary Database reports. [§]Referred to as Asia in previous Global Dietary Database reports.

Supplementary Table 4. Characteristics of global data sources of sugar-sweetened beverage intakes.

	World	Central or Eastern Europe and Central Asia†	High-Income Countries	Latin America and Caribbean	Middle East and North Africa	South Asia†	Southeast and East Asia	Sub-Saharan Africa
Number of surveys (% nationally or subnationally representative)	450 (94.1%)*	89 (100%)	210 (97.6%)	39 (92.3%)	39 (84.6%)	9 (77.8%)	42 (85.7%)	22 (81.8%)
Number of countries represented	118	18	24	22	16	5	18	15
Time period of surveys	1980-2018	1985-2015	1980-2018	1993-2015	1990-2015	1993-2012	1990-2017	1995-2016
Total sample size of surveyed subjects§	2,941,704	390,246	1,289,722	285,156	225,687	81,630	605,044	49,033
By sex								
Females	1,292,940 (44.2%)	154,851 (39.7%)	451,638 (35.0%)	170,074 (59.6%)	119,603 (53.0%)	41,930 (51.4%)	329,007 (54.4%)	25,837 (52.7%)
Males	1,633,578 (55.8%)	235,395 (60.3%)	838,084 (65.0%)	115,082 (40.4%)	106,084 (47.0%)	39,700 (48.6%)	276,037 (45.6%)	23,196(47.3%)
By area of residence								
Urban	2,046,673 (69.9%)	246,967 (63.3%)	974,386 (75.6%)	208,591 (73.1%)	152,482 (67.6%)	23,804 (29.2%)	426,267 (70.5%)	14,176 (28.9%)
Rural	879,845 (30.1%)	143,279 (36.7%)	315,336 (24.4%)	76,565 (26.9%)	73,205 (32.4%)	57,826 (70.8%)	178,777 (29.5%)	34,857 (71.1%)
By education level								
Low education	467,408 (16.0%)	20,509 (5.3%)	130,957 (10.2%)	109,074 (38.3%)	71,817 (31.8%)	35,838 (43.9%)	70,807 (11.7%)	28,405 (57.9%)
Medium education	1,099,716 (37.6%)	131,621 (33.7%)	530,353 (41.1%)	109,359 (38.4%)	68,382 (30.3%)	26,462 (32.4%)	219,211 (36.2%)	14,329 (29.2%)
High education	1,359,394 (46.5%)	238,116 (61.0%)	628,412 (48.7%)	66,723 (23.4%)	85,488 (37.9%)	19,330 (23.7%)	315,026 (52.1%)	6,299 (12.8%)
By adult/youth								
Adults (18+ years)	1,549,750 (53.0%)	119,355 (30.6%)	652,460 (50.6%)	91,677 (32.1%)	110,400 (48.9%)	40,819 (50.0%)	530,735 (87.7%)	4,304 (8.8%)
Children/adolescent (<18 years)	1,376,768 (46.8%)	270,891 (69.4%)	637,262 (49.4%)	193,479 (67.9%)	115,287 (51.1%)	40,811 (50.0%)	74,309 (12.3%)	44,729 (91.2%)
World population represented in 2018ƒ	6,596,636 (87.1%)	310,208 (74.6%)	817,099 (100.0%)	551,485 (86.5%)	476,147 (89.9%)	1,747,992 (96.3%)	2,231,431 (97.2%)	462,274 (43.4%)
Number of surveys by characteristic¥								
By adult/youth								
Adults	191 (42.4%)	29 (32.6%)	93 (44.3%)	12 (30.1%)	12 (30.8%)	4 (44.4%)	31 (73.8%)	10 (45.5%)
Children/adolescents	323 (71.8%)	65 (73.0%)	143 (68.1%)	35 (89.7%)	30 (76.9%)	7 (77.8%)	27 (64.3%)	16 (72.7%)
By area of residence								
Both urban and rural	278 (61.8%)	62 (69.7%)	115 (54.8%)	28 (71.8%)	28 (71.8%)	4 (44.4%)	27 (64.3%)	14 (63.3%)
Only urban	13 (2.9%)	0 (0%)	1 (0.5%)	3 (7.7%)	6 (15.4%)	1 (11.1%)	0 (0%)	2 (9.0%)
Only rural	7 (1.6%)	0 (0%)	0 (0%)	0 (0%)	0 (0%)	3 (33.3%)	1 (2.4%)	3 (13.6%)
Not reported	152 (33.8%)	27 (30.3%)	94 (44.8%)	8 (20.5%)	5 (12.8%)	1 (11.1%)	14 (33.3%)	3 (13.6%)
By dietary assessment method								
FFQ	272 (60.4%)	58 (65.2%)	115 (54.8%)	26 (66.7%)	27 (69.2%)	5 (55.6%)	29 (69.0%)	12 (54.5%)
24hr recall	109 (24.2%)	14 (15.7%)	58 (27.7%)	8 (20.4%)	7 (17.9%)	2 (22.2%)	12 (28.5%)	8 (36.3%)
Household budget survey	54 (12.0%)	17 (19.1%)	37 (17.6%)	0 (0%)	0 (0%)	0 (0%)	0 (0%)	0 (0%)
DHS	15 (3.3%)	0 (0%)	0 (0%)	5 (12.8%)	5 (12.8%)	2 (22.2%)	1 (2.4%)	2 (9.1%)

*Of the 450 surveys reporting data on sugar-sweetened beverages, 46.7% of surveys were in High-Income Countries, followed by Central/Eastern Europe and Central Asia (19.8%), Southeast and East Asia (9.3%), Latin American and the Caribbean (8.7%), Middle East and Northern Africa (8.7%), Sub-Saharan Africa (4.9%), and South Asia (2.0%).

§ The total of subjects sampled can correspond to multiple years, thus in some cases this number is larger than the world population represented in 2018 shown in a few rows below.

ƒ Population expressed in millions. Total population of the countries with sugar-sweetened beverage intake data and the percentage in relation to the total population of all countries within that region.

¥ The values represent the absolute number of surveys and the percentage in relation to the total number of surveys within that region.

† In prior GDD reports, the region Central or Eastern Europe and Central Asia was referred as Former Soviet Union, and Southeast and East Asia was referred as Asia.

Supplementary Table 5. Number of dietary surveys for SSB intake globally, regionally, and in the 25 most populous countries by time period.

	1980-1984	1985-1989	1990-1994	1995-1999	2000-2004	2005-2009	2010-2014	2015-2018
World	3	17	52	75	82	87	116	18
World Region								
Central Eastern Europe and Central Asia†	0	2	10	11	29	18	18	1
High-Income Countries	3	15	39	49	36	32	29	7
Latin America and Caribbean	0	0	0	4	2	15	17	1
Mid. East and North Africa	0	0	2	3	6	8	18	2
South Asia†	0	0	1	1	1	2	4	0
Southeast and East Asia	0	0	0	6	8	9	15	4
Sub-Saharan Africa	0	0	0	1	0	3	15	3
Country								
India	0	0	0	0	0	0	3	0
China	0	0	0	0	1	0	0	0
Nigeria	0	0	0	0	0	0	0	0
Pakistan	0	0	0	0	0	1	0	0
Indonesia	0	0	0	0	0	1	0	0
US	0	1	3	3	4	3	4	2
Brazil	0	0	0	0	0	2	1	0
Bangladesh	0	0	1	1	0	0	1	0
Ethiopia	0	0	0	0	0	2	1	0
DR Congo	0	0	0	0	0	0	0	1
Mexico	0	0	0	0	0	1	1	0
Philippines	0	0	0	0	1	1	1	0
Egypt	0	0	0	1	1	1	1	0
Russia	0	0	1	1	1	1	1	0
Tanzania	0	0	0	0	0	0	0	0
Vietnam	0	0	0	0	0	1	1	0
Turkey	0	0	0	0	1	2	1	0
Kenya	0	0	0	0	0	0	1	0
Iran	0	0	0	1	0	2	2	2
Uganda	0	0	0	0	0	0	0	0
Japan	0	0	0	5	3	0	0	0
South Africa	0	0	0	1	0	0	0	0
Sudan	0	0	0	0	0	0	1	0
Afghanistan	0	0	0	0	0	0	0	0

*Countries are ordered top to bottom from most to least populous in 2018 based on 2018 youth (3-19 years) population data. † In prior GDD reports, the region Central or Eastern Europe and Central Asia was referred as Former Soviet Union, and Southeast and East Asia was referred as Asia.

Supplementary Table 6. National mean (95% UI) sugar-sweetened beverage intakes (248 g servings/week) in youth (3-19 years) by sex, age, parental education, and area of residence in the 25 most populous countries in 2018.

	Sex		Age				Parental education			Area of residence	
	Girls	Boys	3-4 years	5-9 years	10-14 years	15-19 years	≤6 years	>6-12 years	>12 years	Rural	Urban
Country	*mean (95% UI)*	*mean (95% UI)*	*mean (95% UI)*	*mean (95% UI)*	*mean (95% UI)*	*mean (95% UI)*	*mean (95% UI)*	*mean (95% UI)*	*mean (95% UI)*	*mean (95% UI)*	*mean (95% UI)*
India	0.2 (0.2-0.4)	0.3 (0.2-0.5)	0.3 (0.2-0.5)	0.3 (0.2-0.5)	0.3 (0.2-0.4)	0.3 (0.2-0.4)	0.2 (0.1-0.3)	0.4 (0.2-0.6)	0.5 (0.3-0.8)	0.1 (0.1-0.2)	0.6 (0.4-1.0)
China	0.5 (0.4-0.6)	0.5 (0.4-0.6)	0.2 (0.2-0.3)	0.4 (0.4-0.6)	0.6 (0.5-0.8)	0.6 (0.5-0.7)	0.4 (0.3-0.5)	0.5 (0.4-0.7)	0.5 (0.4-0.7)	0.5 (0.4-0.6)	0.5 (0.4-0.6)
Nigeria	2.9 (1.7-4.8)	3.0 (1.9-5.1)	1.0 (0.6-1.8)	2.3 (1.3-4.2)	3.7 (2.1-6.7)	4.7 (2.7-8.4)	2.5 (1.5-4.2)	4.1 (2.5-7.0)	4.6 (2.7-8.0)	2.3 (1.4-3.8)	3.6 (2.2-6.1)
Pakistan	6.4 (4.1-10.0)	6.4 (4.2-9.9)	7.2 (4.7-11.3)	6.6 (4.4-10.1)	6.1 (4.1-9.3)	5.7 (3.8-8.7)	4.7 (2.9-7.5)	8.6 (5.4-13.6)	11.5 (7.0-19.6)	2.8 (1.8-4.2)	12.7 (8.1-20.3)
Indonesia	1.7 (1.2-2.3)	1.8 (1.3-2.5)	0.8 (0.5-1.1)	1.6 (1.2-2.2)	2.2 (1.6-2.9)	2.1 (1.6-2.8)	1.6 (1.2-2.2)	1.9 (1.4-2.6)	1.9 (1.4-2.6)	1.7 (1.3-2.3)	1.8 (1.3-2.4)
United States	5.6 (5.3-6.0)	6.8 (6.3-7.2)	2.3 (2.2-2.5)	5.0 (4.7-5.3)	7.4 (7.0-7.9)	8.4 (7.9-8.9)	8.0 (7.4-8.6)	7.9 (7.3-8.5)	5.8 (5.4-6.1)	6.5 (6.2-6.8)	6.2 (5.8-6.6)
Brazil	4.9 (4.2-5.7)	5.4 (4.6-6.3)	2.5 (2.1-2.9)	4.5 (3.9-5.1)	5.9 (5.2-6.8)	6.4 (5.7-7.3)	4.9 (4.3-5.8)	5.3 (4.6-6.2)	5.4 (4.5-6.3)	4.6 (3.9-5.4)	5.2 (4.6-6.0)
Bangladesh	0.3 (0.2-0.5)	0.4 (0.3-0.5)	0.4 (0.3-0.5)	0.4 (0.3-0.5)	0.3 (0.2-0.5)	0.3 (0.2-0.5)	0.2 (0.2-0.4)	0.4 (0.3-0.7)	0.6 (0.4-1.0)	0.2 (0.1-0.2)	0.7 (0.5-1.1)
Ethiopia	4.4 (3.2-5.9)	4.6 (3.3-6.3)	1.5 (1.1-1.9)	3.4 (2.5-4.6)	5.6 (4.1-7.6)	7.0 (5.3-9.3)	3.7 (2.7-5.0)	6.2 (4.5-8.6)	7.0 (4.8-10.1)	4.1 (3.0-5.4)	6.4 (4.8-8.7)
DR Congo	1.1 (0.4-3.1)	1.2 (0.5-3.5)	0.4 (0.1-1.1)	0.9 (0.3-2.6)	1.5 (0.6-4.2)	1.9 (0.7-5.4)	1.0 (0.4-2.8)	1.7 (0.6-4.6)	1.9 (0.7-5.0)	0.9 (0.3-2.6)	1.5 (0.6-4.2)
Mexico	9.5 (8.4-10.8)	10.8 (9.5-12.2)	4.9 (4.4-5.6)	8.9 (7.9-10.0)	11.9 (10.6-13.3)	12.8 (11.5-14.1)	9.7 (8.6-11.0)	10.4 (9.1-11.8)	10.5 (8.9-12.4)	9.1 (8.1-10.3)	10.4 (9.3-11.6)
Philippines	6.3 (5.3-7.4)	5.4 (4.3-6.7)	2.6 (1.9-3.4)	5.4 (4.4-6.4)	7.2 (6.1-8.3)	7.0 (6.0-8.1)	5.1 (4.3-6.0)	6.1 (5.1-7.2)	6.1 (5.1-7.2)	5.7 (4.8-6.7)	6.0 (5.1-7.0)
Egypt	4.5 (3.7-5.5)	3.7 (3.0-4.7)	1.9 (1.5-2.3)	3.9 (3.2-4.7)	5.3 (4.3-6.4)	5.2 (4.3-6.3)	4.1 (3.4-5.0)	4.4 (3.6-5.5)	3.6 (2.9-4.5)	4.4 (3.5-5.3)	3.8 (3.1-4.6)
Russia	3.9 (3.2-4.8)	4.4 (3.6-5.4)	1.4 (1.1-2.0)	3.6 (3.0-4.4)	5.5 (4.6-6.4)	5.7 (4.8-6.7)	4.0 (3.1-5.4)	4.4 (3.6-5.5)	4.1 (3.4-4.9)	4.4 (3.7-5.4)	4.1 (3.4-4.8)
Tanzania	4.7 (2.9-7.8)	5.0 (3.0-8.2)	1.6 (0.9-2.8)	3.8 (2.1-6.8)	6.2 (3.5-10.6)	7.7 (4.4-13.6)	3.8 (2.3-6.4)	6.4 (3.8-10.5)	7.1 (4.2-12.1)	4.1 (2.5-6.8)	6.5 (3.9-10.6)
Vietnam	3.0 (2.3-3.9)	3.0 (2.3-4.0)	1.4 (1.0-1.9)	2.8 (2.2-3.6)	3.8 (3.0-4.8)	3.7 (2.9-4.7)	2.7 (2.2-3.5)	3.3 (2.6-4.2)	3.3 (2.6-4.2)	3.0 (2.4-3.8)	3.1 (2.5-3.9)
Turkey	4.4 (3.3-5.9)	4.5 (3.4-6.0)	1.9 (1.5-2.6)	4.0 (3.1-5.3)	5.5 (4.3-7.1)	5.4 (4.2-7.0)	4.4 (3.4-5.8)	4.8 (3.7-6.2)	3.9 (3.0-5.1)	5.0 (3.8-6.5)	4.3 (3.3-5.6)
Kenya	0.5 (0.3-0.9)	0.7 (0.4-1.1)	0.2 (0.1-0.3)	0.4 (0.3-0.7)	0.7 (0.4-1.2)	0.9 (0.6-1.5)	0.5 (0.3-0.8)	0.8 (0.5-1.3)	0.9 (0.5-1.5)	0.5 (0.3-0.9)	0.8 (0.5-1.4)
Iran	4.1 (3.5-4.8)	4.3 (3.6-5.0)	1.9 (1.6-2.2)	3.9 (3.4-4.5)	5.3 (4.6-6.1)	5.2 (4.6-5.9)	4.2 (3.6-4.9)	4.5 (3.9-5.3)	3.7 (3.1-4.4)	4.6 (3.9-5.4)	4.0 (3.5-4.6)
Uganda	6.6 (4.0-11.2)	6.9 (4.2-11.3)	2.2 (1.2-4.0)	5.2 (2.9-9.5)	8.5 (4.8-15.6)	10.7 (6.0-19.3)	5.6 (3.3-9.5)	9.4 (5.5-15.8)	10.4 (6.0-17.4)	6.0 (3.7-10.0)	9.5 (5.8-15.3)
Japan	4.6 (3.8-5.6)	5.4 (4.4-6.5)	2.2 (1.6-2.9)	4.5 (3.7-5.4)	6.1 (5.1-7.1)	5.9 (5.1-7.0)	4.3 (3.5-5.1)	5.1 (4.3-6.0)	5.1 (4.3-6.0)	4.8 (4.0-5.8)	5.0 (4.3-5.9)
South Africa	5.9 (4.4-7.9)	6.4 (4.7-8.6)	2.0 (1.5-2.6)	4.6 (3.5-6.1)	7.5 (5.7-10.0)	9.5 (7.3-12.4)	4.2 (3.1-5.9)	7.1 (5.3-9.6)	8.0 (5.9-10.8)	4.5 (3.3-6.0)	7.1 (5.3-9.3)
Sudan	4.1 (2.7-6.3)	4.3 (2.8-6.7)	1.4 (0.9-2.1)	3.2 (2.1-4.9)	5.3 (3.5-7.9)	6.6 (4.5-9.9)	3.5 (2.3-5.3)	5.9 (3.8-9.1)	6.6 (4.1-10.5)	3.5 (2.3-5.3)	5.6 (3.7-8.6)
Afghanistan	2.7 (1.3-5.8)	2.9 (1.4-6.0)	3.1 (1.4-7.3)	2.8 (1.3-6.5)	2.6 (1.2-6.1)	2.5 (1.2-5.6)	2.4 (1.2-5.2)	4.5 (2.2-9.4)	6.0 (2.9-12.6)	1.5 (0.7-3.0)	6.8 (3.3-14.9)
Myanmar	5.3 (3.3-8.8)	5.6 (3.6-9.2)	2.3 (1.3-4.4)	4.8 (2.8-8.9)	6.5 (3.8-11.7)	6.4 (3.7-11.9)	5.1 (3.2-8.7)	6.1 (3.9-10.2)	6.2 (3.8-10.2)	5.4 (3.5-8.9)	5.6 (3.6-8.9)

*Data are mean intakes (95% uncertainty interval) in 248g servings per week. The standardized serving size used for this analysis is 248 g (8 oz) serving. Countries are ordered top to bottom from most to least populous based on 2018 youth (3-19 years) population data .

Supplementary Table 7. Absolute difference in sugar-sweetened beverage intakes (248 g serving/week) in youth (3-19 years) by sex, parental education level, and area of residence globally and regionally across 185 countries in 2018.

	World	Central or Eastern Europe and Central Asia†	High-Income Countries	Latin America and Caribbean	Middle East and North Africa	South Asia†	Southeast and East Asia	Sub-Saharan Africa
	mean (95% UI)	*mean (95% UI)*	*mean (95% UI)*	*mean (95% UI)*	*mean (95% UI)*	*mean (95% UI)*	*mean (95% UI)*	*mean (95% UI)*
**Boys vs. Girls**								
Overall	0.13 (-0.09,0.36)	0.41 (-0.07,0.94)	0.87 (0.65,1.09)	0.72 (-0.19,1.58)	-0.07 (-1.08,0.78)	0.06 (-0.25,0.37)	-0.01 (-0.27,0.24)	0.20 (-0.43,0.81)
Age (years)								
3-4	0.08 (-0.09,0.24)	0.14 (-0.12,0.44)	0.33 (0.24,0.41)	0.35 (-0.15,0.84)	-0.05 (-0.54,0.39)	0.07 (-0.36,0.52)	0.00 (-0.13,0.13)	0.07 (-0.31,0.43)
5-9	0.12 (-0.13,0.38)	0.35 (-0.25,1.00)	0.70 (0.52,0.88)	0.65 (-0.25,1.53)	-0.07 (-1.10,0.84)	0.06 (-0.29,0.41)	-0.02 (-0.29,0.24)	0.16 (-0.60,0.96)
10-14	0.16 (-0.18,0.48)	0.54 (-0.34,1.48)	1.04 (0.78,1.30)	0.86 (-0.35,2.05)	-0.08 (-1.40,1.14)	0.06 (-0.26,0.36)	-0.02 (-0.40,0.34)	0.27 (-0.87,1.36)
15-19	0.16 (-0.19,0.50)	0.54 (-0.36,1.55)	1.18 (0.88,1.47)	0.92 (-0.35,2.12)	-0.09 (-1.42,1.13)	0.05 (-0.22,0.33)	0.00 (-0.38,0.35)	0.31 (-1.02,1.63)
Parental education (years)								
0-6	0.07 (-0.20,0.35)	0.39 (-0.20,1.01)	0.89 (0.61,1.19)	0.62 (-0.31,1.51)	-0.11 (-1.39,0.97)	0.04 (-0.23,0.33)	0.02 (-0.34,0.36)	0.16 (-0.50,0.81)
>6-12	0.14 (-0.12,0.38)	0.46 (-0.14,1.15)	0.83 (0.58,1.08)	0.79 (-0.22,1.81)	-0.04 (-1.04,0.89)	0.09 (-0.16,0.36)	-0.03 (-0.28,0.21)	0.28 (-0.78,1.34)
>12	0.30 (0.04,0.54)	0.39 (-0.23,1.04)	0.89 (0.65,1.13)	0.78 (-0.35,1.86)	-0.03 (-0.80,0.68)	0.07 (-1.10,1.19)	-0.04 (-0.32,0.24)	0.29 (-0.80,1.40)
Area of residency								
Rural	0.04 (-0.19,0.27)	0.42 (-0.32,1.24)	0.88 (0.66,1.11)	0.59 (-0.36,1.51)	-0.24 (-1.59,0.90)	0.03 (-0.11,0.17)	-0.04 (-0.38,0.27)	0.16 (-0.48,0.81)
Urban	0.23 (-0.07,0.52)	0.40 (-0.11,0.94)	0.87 (0.64,1.09)	0.75 (-0.19,1.66)	0.04 (-0.89,0.80)	0.12 (-0.58,0.84)	0.02 (-0.22,0.25)	0.26 (-0.73,1.22)
**High vs. Low Educated Parents**								
Overall	1.45 (1.03,1.87)	-0.23 (-1.34,0.73)	-0.23 (-0.60,0.14)	1.50 (-0.03,3.10)	-2.30 (-4.28,-0.86)	3.41 (2.01,5.88)	0.35 (-0.02,0.65)	2.89 (1.41,4.81)
Sex								
Girls	1.33 (0.89,1.77)	-0.23 (-1.35,0.75)	-0.22 (-0.58,0.13)	1.40 (-0.12,3.03)	-2.34 (-4.41,-0.81)	3.41 (1.96,5.97)	0.38 (-0.04,0.71)	2.81 (1.31,4.79)
Boys	1.56 (1.13,2.01)	-0.23 (-1.43,0.84)	-0.23 (-0.66,0.19)	1.59 (-0.04,3.30)	-2.23 (-4.31,-0.81)	3.43 (2.00,5.86)	0.33 (-0.09,0.65)	2.94 (1.38,5.07)
Age (years)								
3-4	0.68 (0.37,1.02)	-0.07 (-0.54,0.33)	-0.10 (-0.24,0.04)	0.73 (-0.06,1.66)	-1.01 (-1.92,-0.33)	4.19 (2.37,7.24)	0.15 (-0.02,0.31)	0.95 (0.37,1.74)
5-9	1.23 (0.80,1.65)	-0.16 (-1.23,0.76)	-0.20 (-0.50,0.10)	1.31 (-0.16,2.89)	-2.12 (-4.07,-0.70)	3.65 (2.12,6.33)	0.32 (-0.06,0.62)	2.21 (0.95,3.98)
10-14	1.74 (1.19,2.26)	-0.28 (-1.84,1.12)	-0.27 (-0.71,0.17)	1.77 (-0.15,3.72)	-2.94 (-5.50,-1.00)	3.21 (1.89,5.59)	0.42 (-0.13,0.83)	3.58 (1.55,6.22)
15-19	1.81 (1.26,2.35)	-0.37 (-2.00,1.13)	-0.31 (-0.80,0.19)	1.93 (-0.06,4.06)	-2.85 (-5.38,-1.00)	2.88 (1.72,5.01)	0.42 (-0.15,0.82)	4.51 (2.08,7.80)
Area of residency								
Rural	1.34 (0.95,1.75)	-0.24 (-1.60,0.95)	-0.11 (-0.47,0.26)	1.60 (0.03,3.32)	-3.51 (-6.67,-1.50)	1.67 (0.96,2.90)	0.02 (-0.43,0.37)	2.18 (0.91,3.88)
Urban	1.01 (0.50,1.51)	-0.22 (-1.29,0.69)	-0.26 (-0.63,0.11)	1.46 (-0.09,3.08)	-1.49 (-2.82,-0.31)	7.01 (3.98,12.43)	0.63 (0.27,0.94)	3.63 (1.69,6.16)
**Urban vs. Rural Areas**								
Overall	1.85 (1.54,2.19)	-0.58 (-1.36,0.09)	-0.01 (-0.31,0.29)	0.36 (-0.61,1.35)	-1.52 (-3.53,-0.09)	2.19 (1.34,3.53)	-0.09 (-0.31,0.10)	1.59 (0.63,2.83)
Sex								
Girls	1.75 (1.43,2.10)	-0.56 (-1.41,0.17)	0.00 (-0.27,0.27)	0.29 (-0.73,1.25)	-1.67 (-3.77,-0.19)	2.13 (1.27,3.48)	-0.12 (-0.39,0.10)	1.55 (0.48,2.88)
Boys	1.94 (1.61,2.31)	-0.59 (-1.55,0.21)	-0.02 (-0.35,0.31)	0.44 (-0.63,1.52)	-1.38 (-3.42,0.05)	2.23 (1.35,3.66)	-0.06 (-0.32,0.15)	1.63 (0.57,3.02)
Age (years)								
3-4	1.01 (0.78,1.32)	-0.21 (-0.62,0.10)	-0.01 (-0.12,0.11)	0.18 (-0.42,0.71)	-0.67 (-1.56,0.00)	2.79 (1.66,4.53)	-0.05 (-0.18,0.05)	0.52 (0.10,1.07)
5-9	1.62 (1.30,1.98)	-0.49 (-1.46,0.19)	-0.01 (-0.25,0.24)	0.33 (-0.69,1.26)	-1.40 (-3.32,-0.03)	2.35 (1.43,3.77)	-0.09 (-0.36,0.10)	1.23 (0.30,2.44)
10-14	2.16 (1.74,2.58)	-0.74 (-2.05,0.33)	-0.01 (-0.37,0.36)	0.41 (-0.85,1.64)	-2.02 (-4.57,-0.10)	2.03 (1.24,3.31)	-0.11 (-0.48,0.17)	2.01 (0.57,3.86)
15-19	2.30 (1.88,2.75)	-0.77 (-2.13,0.36)	-0.01 (-0.41,0.40)	0.41 (-0.93,1.77)	-1.96 (-4.41,-0.15)	1.83 (1.10,3.01)	-0.08 (-0.46,0.20)	2.54 (0.86,4.85)
Parental education (years)								
0-6	1.69 (1.32,2.13)	-0.54 (-1.40,0.17)	0.05 (-0.26,0.36)	0.45 (-0.53,1.43)	-2.18 (-4.90,-0.41)	1.79 (1.02,3.11)	-0.34 (-0.69,-0.09)	1.13 (0.25,2.33)
>6-12	1.99 (1.64,2.35)	-0.66 (-1.63,0.12)	0.13 (-0.18,0.45)	0.17 (-0.99,1.28)	-0.60 (-1.77,0.55)	1.85 (1.06,3.07)	-0.04 (-0.25,0.15)	2.29 (0.79,4.12)
>12	1.37 (1.06,1.71)	-0.52 (-1.45,0.24)	-0.10 (-0.40,0.19)	0.32 (-0.92,1.46)	-0.13 (-1.05,0.73)	7.15 (4.18,12.25)	0.26 (0.04,0.48)	2.59 (1.09,4.56)

*Data are mean absolute difference in intakes (95% uncertainty interval) in 248 g servings per week. All intakes are reported adjusted to 1,300 kcal/day for ages 3-5 years, 1,700 kcal/day for ages 6-10 years, 2,000 kcal/day for ages 11–19 years. The standardized serving size used for this analysis is 248 g (8 oz) serving. † In prior GDD reports, the region Central/ Eastern Europe and Central Asia was referred as Former Soviet Union, and Southeast and East Asia was referred as Asia.

Supplementary Table 8. Absolute difference in sugar-sweetened beverage intakes (248 g serving/week) in boys vs. girls (3-19 years) in the 25 most populous countries in 2018.

	Age					Parental education			Area of residence	
	Overall	3-4 years	5-9 years	10-14 years	15-19 years	≤6 years	>6-12 years	>12 years	Rural	Urban
Country	*mean (95% UI)*	*mean (95% UI)*	*mean (95% UI)*	*mean (95% UI)*	*mean (95% UI)*	*mean (95% UI)*	*mean (95% UI)*	*mean (95% UI)*	*mean (95% UI)*	*mean (95% UI)*
India	0.09 (0.01,0.22)	0.10 (0.01,0.25)	0.10 (0.01,0.23)	0.09 (0.01,0.21)	0.08 (0.01,0.19)	0.06 (0.01,0.16)	0.12 (0.02,0.28)	0.16 (0.02,0.39)	0.04 (0.01,0.09)	0.19 (0.03,0.47)
China	0.01 (-0.11,0.13)	0.01 (-0.05,0.06)	0.01 (-0.10,0.12)	0.01 (-0.14,0.16)	0.01 (-0.14,0.16)	0.01 (-0.10,0.12)	0.01 (-0.12,0.14)	0.01 (-0.12,0.14)	0.01 (-0.11,0.13)	0.01 (-0.12,0.13)
Nigeria	0.17 (-1.43,1.78)	0.05 (-0.92,1.07)	0.13 (-2.19,2.61)	0.24 (-3.46,3.98)	0.21 (-4.49,4.75)	0.16 (-1.79,2.04)	0.22 (-3.05,3.58)	0.21 (-3.54,3.85)	0.12 (-1.61,1.90)	0.22 (-2.57,3.01)
Pakistan	0.01 (-2.07,1.99)	0.01 (-2.36,2.23)	0.01 (-2.15,2.07)	0.01 (-1.99,1.92)	0.01 (-1.85,1.77)	0.01 (-1.55,1.49)	0.01 (-2.83,2.65)	0.01 (-3.92,3.65)	0.00 (-0.91,0.87)	0.01 (-4.17,4.08)
Indonesia	0.17 (-0.31,0.67)	0.08 (-0.14,0.31)	0.16 (-0.28,0.62)	0.21 (-0.38,0.82)	0.21 (-0.37,0.81)	0.16 (-0.28,0.62)	0.19 (-0.34,0.75)	0.19 (-0.33,0.74)	0.17 (-0.30,0.66)	0.18 (-0.31,0.68)
United States	1.13 (0.75,1.51)	0.42 (0.28,0.57)	0.91 (0.61,1.21)	1.35 (0.90,1.80)	1.52 (1.02,2.04)	1.45 (0.97,1.94)	1.43 (0.95,1.91)	1.04 (0.70,1.40)	1.17 (0.78,1.57)	1.12 (0.74,1.50)
Brazil	0.45 (-0.44,1.39)	0.22 (-0.22,0.67)	0.40 (-0.37,1.21)	0.53 (-0.51,1.62)	0.57 (-0.54,1.75)	0.43 (-0.43,1.35)	0.46 (-0.45,1.44)	0.47 (-0.46,1.46)	0.40 (-0.39,1.26)	0.46 (-0.45,1.41)
Bangladesh	0.03 (-0.06,0.11)	0.03 (-0.07,0.13)	0.03 (-0.06,0.12)	0.02 (-0.06,0.11)	0.02 (-0.05,0.10)	0.02 (-0.04,0.08)	0.03 (-0.07,0.14)	0.04 (-0.10,0.20)	0.01 (-0.03,0.05)	0.05 (-0.12,0.24)
Ethiopia	0.23 (-0.88,1.38)	0.08 (-0.28,0.45)	0.18 (-0.66,1.05)	0.29 (-1.07,1.73)	0.37 (-1.34,2.17)	0.19 (-0.74,1.13)	0.32 (-1.22,1.96)	0.35 (-1.40,2.18)	0.21 (-0.79,1.25)	0.32 (-1.28,2.00)
DR Congo	0.12 (-0.31,0.78)	0.04 (-0.10,0.27)	0.09 (-0.24,0.63)	0.15 (-0.39,1.01)	0.19 (-0.48,1.26)	0.10 (-0.25,0.66)	0.16 (-0.45,1.10)	0.18 (-0.46,1.22)	0.09 (-0.25,0.62)	0.15 (-0.38,0.98)
Mexico	1.25 (-0.13,2.60)	0.62 (-0.06,1.28)	1.11 (-0.10,2.29)	1.49 (-0.14,3.06)	1.60 (-0.15,3.29)	1.19 (-0.13,2.50)	1.29 (-0.15,2.66)	1.30 (-0.13,2.74)	1.13 (-0.12,2.35)	1.28 (-0.14,2.67)
Philippines	-0.87 (-2.22,0.47)	-0.39 (-1.03,0.21)	-0.80 (-2.07,0.43)	-1.08 (-2.72,0.58)	-1.06 (-2.66,0.57)	-0.76 (-1.95,0.41)	-0.92 (-2.33,0.50)	-0.91 (-2.31,0.49)	-0.86 (-2.19,0.46)	-0.90 (-2.25,0.49)
Egypt	-0.77 (-1.47,-0.10)	-0.35 (-0.68,-0.04)	-0.73 (-1.39,-0.09)	-0.98 (-1.88,-0.12)	-0.97 (-1.83,-0.12)	-0.77 (-1.47,-0.09)	-0.82 (-1.59,-0.10)	-0.67 (-1.31,-0.08)	-0.81 (-1.57,-0.10)	-0.70 (-1.35,-0.09)
Russia	0.48 (-0.44,1.45)	0.17 (-0.16,0.52)	0.42 (-0.38,1.29)	0.64 (-0.56,1.92)	0.66 (-0.59,1.98)	0.47 (-0.43,1.45)	0.51 (-0.47,1.58)	0.47 (-0.43,1.44)	0.51 (-0.47,1.56)	0.47 (-0.43,1.43)
Tanzania	0.24 (-2.48,2.78)	0.07 (-1.53,1.71)	0.18 (-3.48,4.16)	0.36 (-5.98,6.43)	0.29 (-7.37,7.92)	0.16 (-2.91,3.16)	0.34 (-4.76,5.35)	0.28 (-5.05,5.95)	0.15 (-2.90,3.33)	0.32 (-4.49,4.99)
Vietnam	0.02 (-0.82,0.81)	0.01 (-0.36,0.37)	0.03 (-0.75,0.77)	0.04 (-1.00,1.02)	0.04 (-0.98,1.00)	0.01 (-0.74,0.73)	0.02 (-0.89,0.88)	0.02 (-0.89,0.87)	0.02 (-0.81,0.79)	0.02 (-0.85,0.83)
Turkey	0.10 (-1.10,1.27)	0.04 (-0.48,0.56)	0.09 (-1.00,1.16)	0.13 (-1.35,1.57)	0.12 (-1.32,1.54)	0.10 (-1.10,1.27)	0.11 (-1.17,1.36)	0.09 (-0.98,1.13)	0.11 (-1.22,1.44)	0.10 (-1.06,1.23)
Kenya	0.10 (-0.06,0.37)	0.03 (-0.02,0.12)	0.08 (-0.05,0.28)	0.13 (-0.08,0.46)	0.16 (-0.10,0.58)	0.08 (-0.05,0.29)	0.13 (-0.08,0.50)	0.15 (-0.09,0.56)	0.09 (-0.06,0.32)	0.14 (-0.08,0.51)
Iran	0.18 (-0.52,0.87)	0.09 (-0.22,0.39)	0.18 (-0.47,0.82)	0.24 (-0.64,1.10)	0.24 (-0.63,1.09)	0.19 (-0.52,0.88)	0.20 (-0.55,0.96)	0.16 (-0.46,0.78)	0.20 (-0.57,0.97)	0.18 (-0.49,0.84)
Uganda	0.27 (-3.73,4.19)	0.12 (-2.31,2.47)	0.17 (-5.57,5.79)	0.44 (-8.89,9.52)	0.52 (-10.72,11.99)	0.19 (-4.46,4.90)	0.43 (-7.35,8.15)	0.39 (-7.80,8.92)	0.26 (-4.45,4.90)	0.36 (-6.62,7.34)
Japan	0.73 (-0.23,1.73)	0.32 (-0.10,0.77)	0.66 (-0.20,1.57)	0.89 (-0.28,2.08)	0.87 (-0.27,2.04)	0.62 (-0.19,1.47)	0.75 (-0.23,1.76)	0.74 (-0.23,1.75)	0.71 (-0.22,1.67)	0.74 (-0.22,1.73)
South Africa	0.47 (-1.05,2.10)	0.15 (-0.34,0.68)	0.36 (-0.79,1.58)	0.59 (-1.27,2.59)	0.74 (-1.58,3.25)	0.32 (-0.72,1.47)	0.54 (-1.20,2.42)	0.60 (-1.35,2.71)	0.33 (-0.74,1.50)	0.54 (-1.20,2.40)
Sudan	0.19 (-1.11,1.55)	0.06 (-0.36,0.50)	0.15 (-0.84,1.16)	0.24 (-1.39,1.92)	0.31 (-1.73,2.43)	0.16 (-0.91,1.27)	0.27 (-1.54,2.17)	0.30 (-1.72,2.46)	0.16 (-0.91,1.30)	0.26 (-1.49,2.02)
Afghanistan	0.19 (-1.60,2.14)	0.22 (-3.74,4.35)	0.19 (-3.45,3.82)	0.17 (-3.32,3.81)	0.17 (-2.66,3.47)	0.17 (-1.88,2.36)	0.37 (-3.32,3.97)	0.49 (-4.05,5.11)	0.10 (-1.12,1.48)	0.49 (-5.71,6.94)
Myanmar	0.33 (-2.87,3.63)	0.14 (-2.52,2.76)	0.32 (-5.01,5.86)	0.45 (-6.05,7.63)	0.36 (-6.46,7.29)	0.35 (-3.88,4.74)	0.32 (-4.69,5.73)	0.44 (-4.71,5.59)	0.31 (-3.86,4.67)	0.38 (-3.99,4.70)

*Data are mean absolute difference in intakes (95% uncertainty interval) in 248 g servings per week. The standardized serving size used for this analysis is 248 g (8 oz) serving. Countries are ordered top to bottom from most to least populous based on 2018 youth (3-19 years) population data .

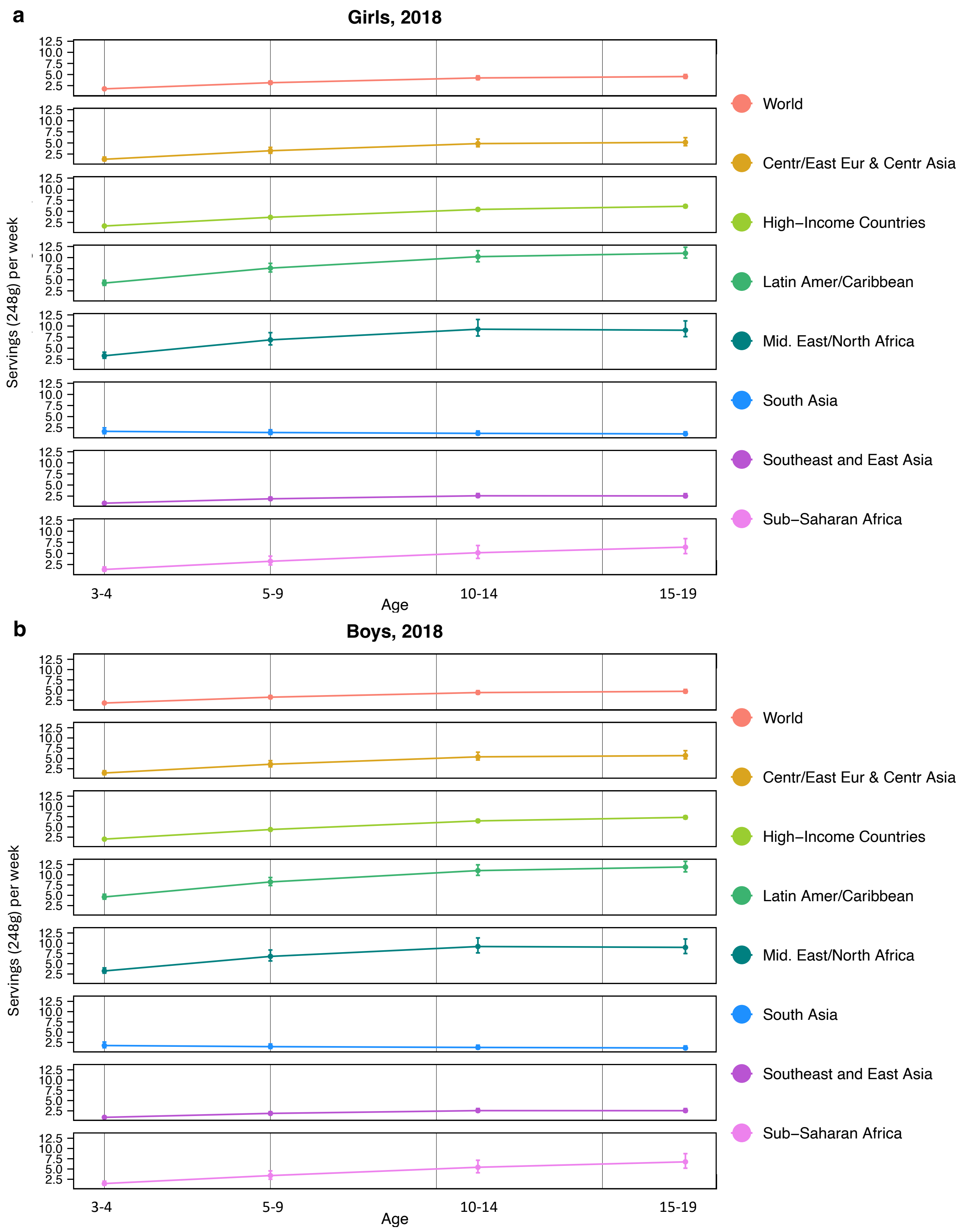

Supplementary Figure 2. Global and regional sugar-sweetened beverage intakes (248 g servings/week) by age among youth (3-19 years) girls and boys in 2018. SSBs were defined as any beverage with added sugars and ≥209 kJ (50 kcal) per 237 g serving, including commercial or homemade beverages, soft drinks, energy drinks, fruit drinks, punch, lemonade, and aguas frescas. This definition excludes 100% fruit and vegetable juices, non-caloric artificially sweetened drinks, and sweetened milk. The standardized serving size used for this analysis is 258 g (8 oz) serving. The filled circles represent the mean SSBs intake (248 g serving/week) and the error bars the 95% UIs. Age groups are 3-4, 5-9, 10-14, and 15-19 years. In prior GDD reports, the region Central or Eastern Europe and Central Asia was referred as Former Soviet Union, and Southeast and East Asia was referred as Asia.

GDD, Global Dietary Database; SSBs, sugar-sweetened beverages; UIs, uncertainty intervals.

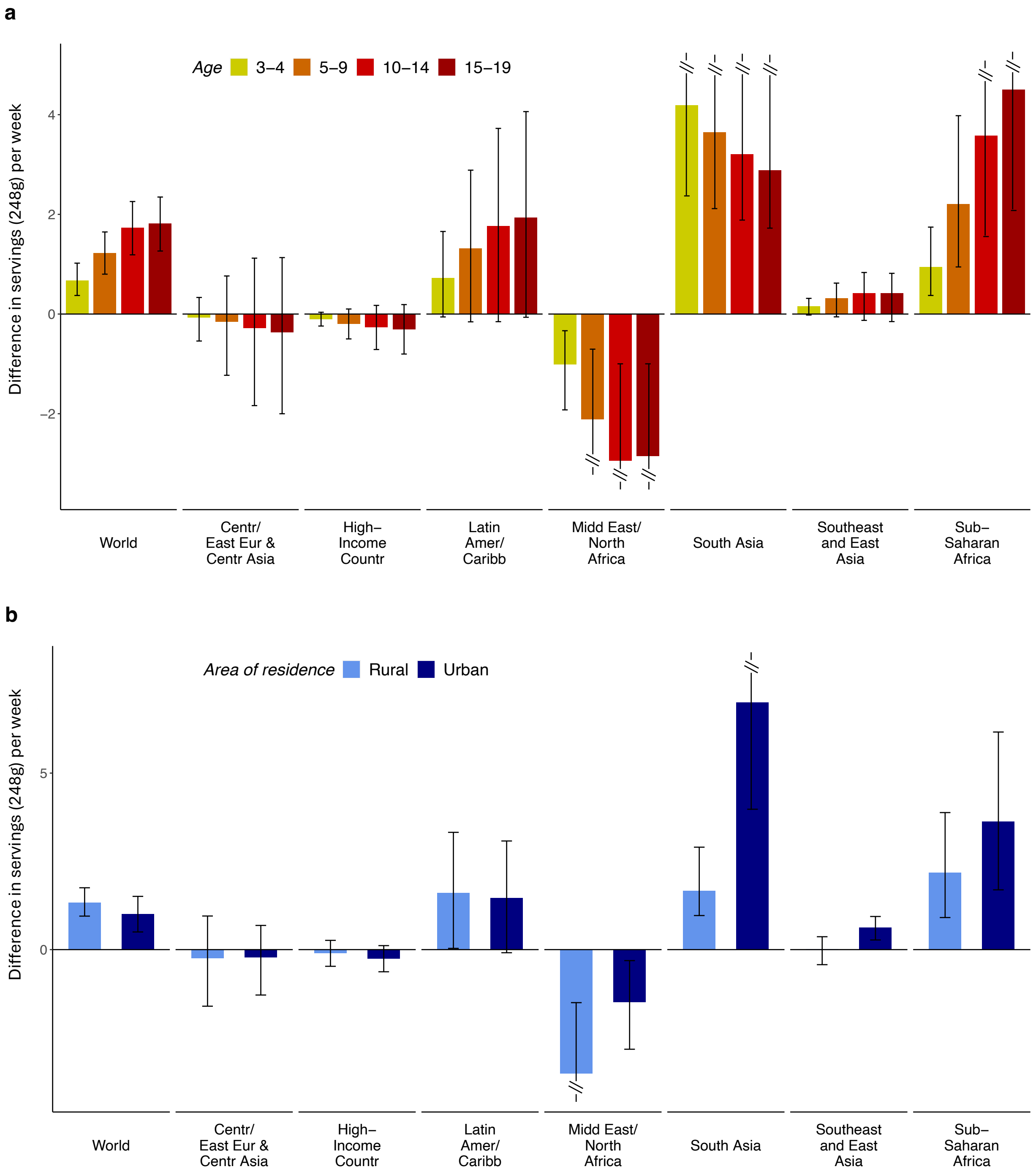

Supplementary Figure 3. Difference in sugar-sweetened beverage intakes (248 g serving/week) between youth (3-19 years) from high vs. low educated parents by age and by area of residence. SSBs were defined as any beverage with added sugars and ≥209 kJ (50 kcal) per 237 g serving, including commercial or homemade beverages, soft drinks, energy drinks, fruit drinks, punch, lemonade, and aguas frescas. This definition excludes 100% fruit and vegetable juices, non-caloric artificially sweetened drinks, and sweetened milk. The standardized serving size used for this analysis is 248 grams (8 oz) serving. The filled bars represent the mean difference in SSBs intake (248 g serving/week) and the error bars the 95% UIs. Values were truncated at -3.5 and 5.0 servings in the top panel (a), and at -4.0 to 8.0 in the bottom panel (b) to better represent the distribution of intakes. Upper and lower 95% UIs above or below those values are displayed with a dashed line. Colors represent the age category as “20-30 years” (orange), “40-59 years” (red) or “60+ years” (dark red); and area of residence as “rural” (light blue) or “urban” (dark blue). In prior GDD reports, the region Central/ Eastern Europe and Central Asia was referred as Former Soviet Union, and Southeast and East Asia was referred as Asia.

Centr/East Eur & Centr Asia, Central/Eastern Europe and Central Asia; GDD, Global Dietary Database; Latin Amer/Caribbean, Latin America/Caribbean; SSBs, sugar-sweetened beverages; UIs, uncertainty intervals.

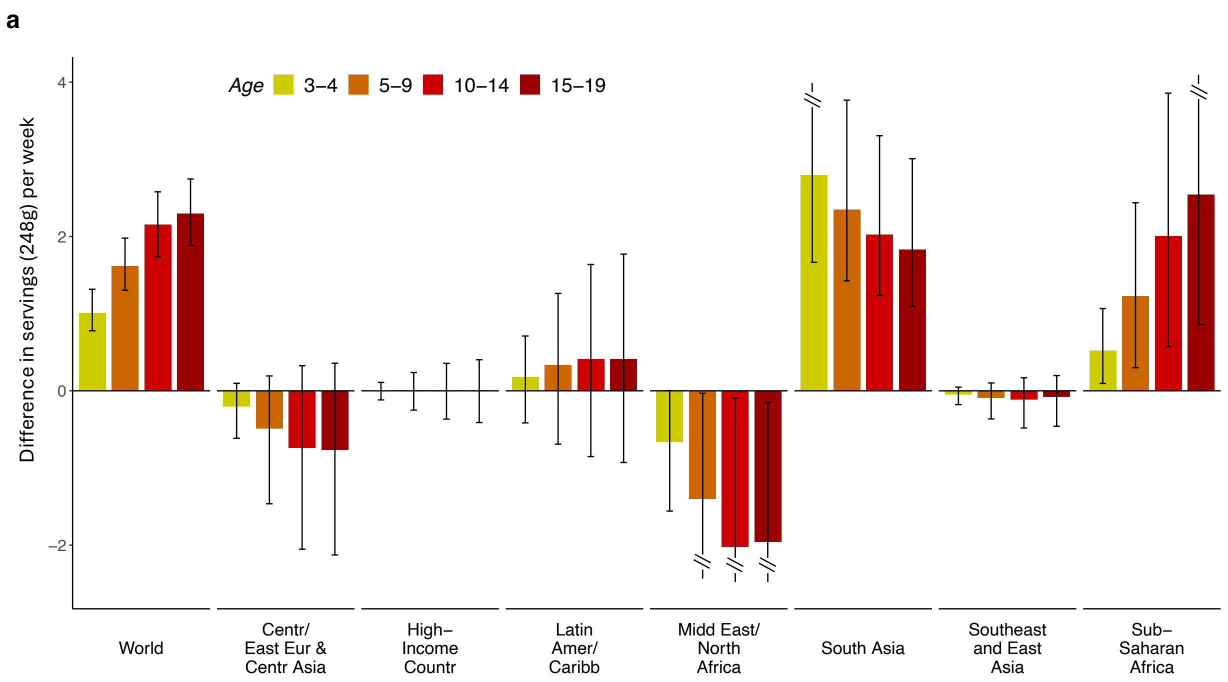

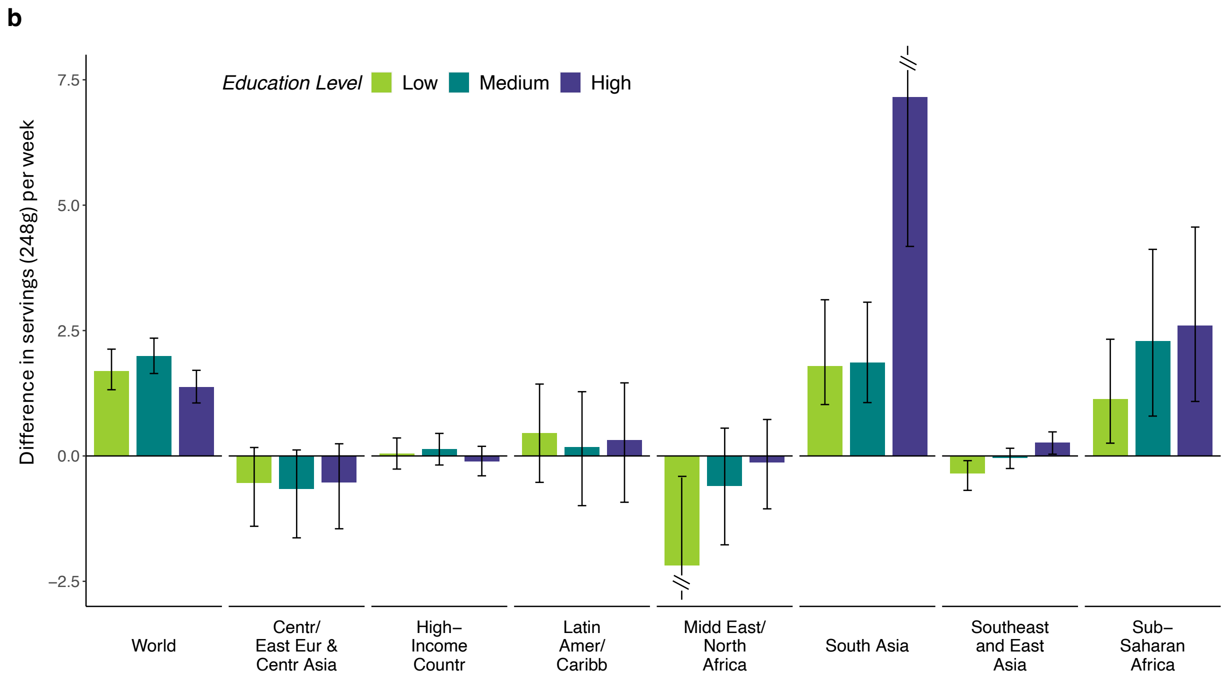

Supplementary Figure 4. Difference in sugar-sweetened beverage intakes (248 g serving/week) between youth (3-19 years) from urban vs. rural areas by age and by parental education level in 2018. SSBs were defined as any beverage with added sugars and ≥209 kJ (50 kcal) per 237 g serving, including commercial or homemade beverages, soft drinks, energy drinks, fruit drinks, punch, lemonade, and aguas frescas. This definition excludes 100% fruit and vegetable juices, non-caloric artificially sweetened drinks, and sweetened milk. The standardized serving size used for this analysis is 248 grams (8 oz) serving. The filled bars represent the mean difference in SSBs intake (248 g serving/week) and the error bars the 95% UIs. Values were truncated at -2.5 and 4.0 servings in the top panel (a), and at -2.5 to 7.5 in the bottom panel (b) to better represent the distribution of intakes. Upper and lower 95% UIs above or below those values are displayed with a dashed line. Colors represent the age category as "20-30 years" (orange), "40-59 years" (red) or "60+ years" (dark red); and education level as "low" 0 to 6 years of education (light green), "medium" >6 years to 12 years of education (dark green), or "high" >12 years of education (purple). In prior GDD reports, the region Central/ Eastern Europe and Central Asia was referred as Former Soviet Union, and Southeast and East Asia was referred as Asia.

Centr/East Eur & Centr Asia, Central/Eastern Europe and Central Asia; GDD, Global Dietary Database; Latin Amer/Caribbean, Latin America/Caribbean; SSBs, sugar-sweetened beverages; UIs, uncertainty intervals.

Supplementary Table 9. Absolute difference in sugar-sweetened beverage intakes (248 g serving/week) in youth (3-19 years) of high vs. low educated parents in the 25 most populous countries in 2018.

	Sex			Age			Area of residence	
	Overall	Girls	Boys	5-9 years	10-14 years	15-19 years	Rural	Urban
Country	*mean (95% UI)*	*mean (95% UI)*	*mean (95% UI)*	*mean (95% UI)*	*mean (95% UI)*	*mean (95% UI)*	*mean (95% UI)*	*mean (95% UI)*
India	0.29 (0.13,0.57)	0.24 (0.11,0.48)	0.34 (0.15,0.66)	0.34 (0.15,0.64)	0.31 (0.14,0.60)	0.29 (0.13,0.56)	0.26 (0.12,0.51)	0.13 (0.06,0.26)
China	0.08 (0.03,0.14)	0.08 (0.03,0.14)	0.08 (0.03,0.14)	0.04 (0.01,0.07)	0.08 (0.03,0.13)	0.10 (0.04,0.17)	0.10 (0.04,0.17)	0.08 (0.03,0.14)
Nigeria	2.11 (0.09,5.27)	1.99 (-0.42,5.98)	2.10 (-0.54,6.16)	0.67 (-0.40,2.32)	1.54 (-0.89,5.48)	2.59 (-1.35,8.90)	3.19 (-1.99,11.78)	1.59 (-0.33,4.78)
Pakistan	6.72 (2.93,13.79)	6.71 (2.84,14.20)	6.72 (2.88,13.88)	7.65 (3.24,15.57)	7.00 (3.02,14.52)	6.46 (2.81,13.44)	5.99 (2.58,12.37)	2.92 (1.18,6.20)
Indonesia	0.31 (0.12,0.56)	0.29 (0.11,0.53)	0.32 (0.12,0.59)	0.14 (0.05,0.26)	0.28 (0.11,0.51)	0.38 (0.14,0.68)	0.37 (0.14,0.66)	0.30 (0.11,0.55)
United States	-2.21 (-2.76,-1.71)	-2.01 (-2.50,-1.56)	-2.41 (-3.03,-1.86)	-0.82 (-1.03,-0.64)	-1.78 (-2.22,-1.37)	-2.63 (-3.29,-2.04)	-2.98 (-3.73,-2.31)	-2.30 (-2.83,-1.79)
Brazil	0.42 (-0.43,1.29)	0.39 (-0.41,1.23)	0.43 (-0.45,1.35)	0.20 (-0.21,0.63)	0.36 (-0.37,1.12)	0.48 (-0.50,1.49)	0.52 (-0.54,1.60)	0.37 (-0.38,1.17)
Bangladesh	0.34 (0.15,0.70)	0.33 (0.14,0.69)	0.36 (0.15,0.74)	0.39 (0.17,0.78)	0.36 (0.15,0.74)	0.33 (0.14,0.70)	0.31 (0.13,0.65)	0.15 (0.06,0.33)
Ethiopia	3.23 (1.53,5.74)	3.12 (1.46,5.60)	3.30 (1.54,6.00)	1.05 (0.50,1.82)	2.45 (1.16,4.36)	3.99 (1.87,7.18)	5.00 (2.36,8.94)	2.88 (1.34,5.22)
DR Congo	0.86 (0.26,2.53)	0.82 (0.24,2.42)	0.90 (0.27,2.68)	0.29 (0.09,0.85)	0.68 (0.21,2.03)	1.11 (0.33,3.31)	1.40 (0.41,4.10)	0.68 (0.20,2.06)
Mexico	0.81 (-0.86,2.55)	0.76 (-0.80,2.41)	0.86 (-0.89,2.71)	0.40 (-0.41,1.26)	0.71 (-0.75,2.25)	0.95 (-1.00,2.98)	1.03 (-1.08,3.22)	0.74 (-0.74,2.32)
Philippines	0.98 (0.38,1.61)	1.05 (0.41,1.75)	0.90 (0.36,1.53)	0.43 (0.16,0.78)	0.90 (0.35,1.51)	1.20 (0.47,1.97)	1.18 (0.47,1.92)	0.96 (0.37,1.59)
Egypt	-0.48 (-1.04,0.04)	-0.52 (-1.14,0.05)	-0.43 (-0.95,0.04)	-0.22 (-0.47,0.02)	-0.45 (-0.98,0.04)	-0.61 (-1.33,0.06)	-0.60 (-1.31,0.06)	-0.51 (-1.09,0.05)
Russia	0.02 (-1.14,0.92)	0.01 (-1.09,0.88)	0.02 (-1.20,0.97)	0.01 (-0.41,0.34)	0.01 (-1.00,0.82)	0.02 (-1.50,1.21)	0.02 (-1.57,1.25)	0.02 (-1.22,0.99)
Tanzania	3.22 (0.20,7.73)	3.09 (-0.72,8.94)	3.22 (-0.54,9.25)	1.01 (-0.53,3.44)	2.43 (-1.30,8.22)	4.06 (-2.14,13.50)	4.90 (-2.65,15.88)	2.63 (-0.44,7.64)
Vietnam	0.53 (0.20,0.91)	0.53 (0.20,0.92)	0.53 (0.20,0.92)	0.24 (0.09,0.43)	0.49 (0.19,0.86)	0.66 (0.25,1.12)	0.64 (0.25,1.09)	0.52 (0.20,0.90)
Turkey	-0.51 (-1.17,0.05)	-0.50 (-1.16,0.05)	-0.52 (-1.19,0.05)	-0.22 (-0.51,0.02)	-0.46 (-1.05,0.04)	-0.63 (-1.42,0.06)	-0.62 (-1.41,0.06)	-0.57 (-1.29,0.05)
Kenya	0.40 (0.17,0.83)	0.36 (0.15,0.76)	0.44 (0.18,0.92)	0.13 (0.05,0.26)	0.30 (0.13,0.63)	0.49 (0.20,1.03)	0.62 (0.26,1.27)	0.35 (0.14,0.73)
Iran	-0.49 (-1.06,0.05)	-0.48 (-1.04,0.04)	-0.50 (-1.08,0.05)	-0.22 (-0.48,0.02)	-0.46 (-0.99,0.04)	-0.62 (-1.34,0.06)	-0.61 (-1.33,0.06)	-0.54 (-1.18,0.05)
Uganda	4.70 (0.19,11.04)	4.54 (-1.22,13.26)	4.72 (-1.23,13.41)	1.51 (-0.83,5.48)	3.64 (-2.26,12.74)	5.68 (-3.63,20.56)	6.78 (-4.94,24.27)	4.10 (-0.87,11.41)
Japan	0.82 (0.33,1.31)	0.75 (0.31,1.22)	0.87 (0.36,1.42)	0.36 (0.14,0.62)	0.73 (0.30,1.21)	0.99 (0.40,1.59)	0.97 (0.40,1.54)	0.78 (0.32,1.28)
South Africa	3.72 (1.91,6.06)	3.57 (1.81,5.87)	3.87 (1.95,6.37)	1.19 (0.61,1.93)	2.80 (1.42,4.56)	4.57 (2.35,7.48)	5.73 (2.95,9.28)	2.69 (1.34,4.50)
Sudan	3.06 (1.32,5.87)	2.96 (1.27,5.80)	3.11 (1.36,6.01)	0.98 (0.43,1.94)	2.32 (1.00,4.48)	3.81 (1.64,7.28)	4.79 (2.07,9.10)	2.55 (1.07,4.95)
Afghanistan	3.45 (0.87,8.76)	3.25 (0.24,9.25)	3.53 (0.50,9.69)	3.64 (-0.66,11.92)	3.52 (-0.37,11.23)	3.15 (-0.36,10.27)	2.89 (-0.34,9.99)	1.79 (0.19,5.57)
Myanmar	0.97 (-2.30,4.61)	0.94 (-3.65,5.86)	0.94 (-3.82,6.20)	0.41 (-2.25,3.45)	0.81 (-4.16,6.57)	1.11 (-6.10,8.68)	1.07 (-6.06,8.91)	0.91 (-3.19,5.88)

*Data are mean absolute difference in intakes (95% uncertainty interval) in 248 g servings per week. The standardized serving size used for this analysis is 248 g (8 oz) serving. Countries are ordered top to bottom from most to least populous based on 2018 youth (3-19 years) population data.

Supplementary Table 10. Absolute difference in sugar-sweetened beverage intakes (248 g serving/week) in urban vs. rural areas in the 25 most populous countries in youth (3-19 years) in 2018.

	Sex			Age			Parental education		
	Overall	Girls	Boys	5-9 years	10-14 years	15-19 years	≤6 years	>6-12 years	>12
Country	*mean (95% UI)*	*mean (95% UI)*	*mean (95% UI)*	*mean (95% UI)*	*mean (95% UI)*	*mean (95% UI)*	*mean (95% UI)*	*mean (95% UI)*	*mean (95% UI)*
India	0.47 (0.25,0.83)	0.39 (0.21,0.70)	0.54 (0.28,0.98)	0.54 (0.29,0.96)	0.49 (0.26,0.88)	0.46 (0.24,0.82)	0.42 (0.22,0.76)	0.34 (0.17,0.64)	0.62 (0.32,1.11)
China	0.02 (-0.02,0.06)	0.02 (-0.02,0.06)	0.02 (-0.02,0.06)	0.01 (-0.01,0.03)	0.02 (-0.02,0.05)	0.02 (-0.02,0.07)	0.02 (-0.02,0.07)	0.02 (-0.02,0.05)	0.02 (-0.02,0.06)
Nigeria	1.30 (-0.18,3.54)	1.23 (-0.73,4.14)	1.33 (-0.74,4.26)	0.39 (-0.45,1.61)	0.97 (-0.96,3.95)	1.55 (-1.65,6.19)	2.02 (-1.91,7.96)	1.07 (-0.61,3.68)	1.82 (-1.15,5.97)
Pakistan	9.93 (5.88,16.71)	9.93 (5.66,17.18)	9.88 (5.73,17.06)	11.26 (6.44,19.10)	10.31 (6.10,17.30)	9.55 (5.61,16.08)	8.85 (5.17,15.21)	7.32 (4.04,12.96)	13.44 (7.55,23.66)
Indonesia	0.07 (-0.06,0.22)	0.07 (-0.06,0.21)	0.07 (-0.06,0.23)	0.03 (-0.03,0.10)	0.06 (-0.06,0.20)	0.09 (-0.08,0.27)	0.09 (-0.07,0.26)	0.07 (-0.06,0.20)	0.08 (-0.07,0.24)
United States	-0.29 (-0.67,0.09)	-0.26 (-0.60,0.08)	-0.32 (-0.73,0.10)	-0.11 (-0.25,0.03)	-0.23 (-0.53,0.07)	-0.35 (-0.79,0.11)	-0.39 (-0.90,0.12)	-0.37 (-0.84,0.12)	-0.37 (-0.84,0.11)
Brazil	0.63 (0.14,1.14)	0.60 (0.13,1.09)	0.66 (0.15,1.20)	0.30 (0.07,0.55)	0.55 (0.12,0.99)	0.73 (0.16,1.32)	0.78 (0.17,1.41)	0.60 (0.13,1.11)	0.65 (0.14,1.18)
Bangladesh	0.55 (0.34,0.88)	0.53 (0.32,0.87)	0.57 (0.34,0.93)	0.64 (0.40,0.97)	0.58 (0.35,0.93)	0.54 (0.32,0.89)	0.50 (0.29,0.83)	0.39 (0.22,0.66)	0.71 (0.42,1.18)
Ethiopia	2.36 (1.18,3.92)	2.29 (1.14,3.85)	2.40 (1.21,4.05)	0.76 (0.39,1.22)	1.79 (0.90,2.96)	2.92 (1.45,4.87)	3.67 (1.82,6.07)	1.94 (0.95,3.28)	3.24 (1.68,5.44)
DR Congo	0.55 (0.17,1.66)	0.52 (0.16,1.58)	0.57 (0.18,1.79)	0.18 (0.06,0.56)	0.43 (0.14,1.32)	0.71 (0.22,2.16)	0.89 (0.28,2.73)	0.46 (0.14,1.41)	0.77 (0.24,2.33)
Mexico	1.26 (0.27,2.29)	1.17 (0.25,2.15)	1.33 (0.28,2.43)	0.62 (0.13,1.12)	1.10 (0.24,2.01)	1.47 (0.32,2.68)	1.58 (0.34,2.87)	1.19 (0.25,2.23)	1.29 (0.27,2.35)
Philippines	0.24 (-0.21,0.69)	0.26 (-0.23,0.73)	0.22 (-0.20,0.65)	0.10 (-0.10,0.31)	0.22 (-0.19,0.64)	0.29 (-0.27,0.85)	0.29 (-0.26,0.83)	0.21 (-0.18,0.60)	0.25 (-0.22,0.72)
Egypt	-0.57 (-1.12,-0.07)	-0.63 (-1.23,-0.08)	-0.52 (-1.02,-0.06)	-0.26 (-0.51,-0.03)	-0.54 (-1.05,-0.07)	-0.73 (-1.43,-0.09)	-0.72 (-1.42,-0.09)	-0.57 (-1.12,-0.07)	-0.62 (-1.21,-0.08)
Russia	-0.37 (-1.03,0.24)	-0.35 (-0.97,0.23)	-0.39 (-1.12,0.25)	-0.13 (-0.38,0.08)	-0.32 (-0.90,0.21)	-0.49 (-1.35,0.31)	-0.50 (-1.39,0.32)	-0.36 (-1.02,0.24)	-0.39 (-1.11,0.25)
Tanzania	2.32 (-0.37,6.02)	2.26 (-1.37,7.15)	2.36 (-1.54,7.13)	0.74 (-0.77,2.90)	1.69 (-1.92,6.89)	2.82 (-3.11,11.04)	3.46 (-3.51,13.84)	1.75 (-1.19,5.94)	2.98 (-1.90,9.52)
Vietnam	0.12 (-0.11,0.37)	0.12 (-0.11,0.37)	0.12 (-0.11,0.38)	0.05 (-0.05,0.17)	0.11 (-0.11,0.35)	0.15 (-0.14,0.46)	0.15 (-0.14,0.46)	0.11 (-0.10,0.34)	0.13 (-0.12,0.40)
Turkey	-0.64 (-1.33,-0.09)	-0.63 (-1.32,-0.08)	-0.65 (-1.35,-0.08)	-0.28 (-0.58,-0.04)	-0.58 (-1.21,-0.08)	-0.79 (-1.63,-0.10)	-0.78 (-1.62,-0.10)	-0.64 (-1.32,-0.08)	-0.68 (-1.42,-0.09)
Kenya	0.30 (0.14,0.58)	0.27 (0.13,0.52)	0.32 (0.15,0.65)	0.10 (0.05,0.18)	0.23 (0.11,0.43)	0.37 (0.17,0.71)	0.46 (0.21,0.89)	0.23 (0.11,0.46)	0.39 (0.18,0.76)
Iran	-0.61 (-1.21,-0.08)	-0.59 (-1.17,-0.08)	-0.62 (-1.23,-0.08)	-0.27 (-0.54,-0.03)	-0.57 (-1.12,-0.07)	-0.77 (-1.52,-0.10)	-0.76 (-1.50,-0.10)	-0.62 (-1.23,-0.08)	-0.66 (-1.32,-0.09)
Uganda	3.36 (-0.41,8.47)	3.27 (-2.13,9.99)	3.37 (-2.02,10.44)	1.11 (-1.18,4.14)	2.55 (-2.65,9.97)	4.11 (-3.98,15.51)	5.22 (-5.29,18.61)	2.75 (-1.66,8.66)	4.67 (-2.75,13.75)
Japan	0.20 (-0.19,0.57)	0.18 (-0.17,0.54)	0.21 (-0.20,0.62)	0.09 (-0.08,0.26)	0.18 (-0.17,0.52)	0.24 (-0.23,0.69)	0.24 (-0.23,0.68)	0.17 (-0.16,0.49)	0.20 (-0.19,0.59)
South Africa	2.60 (1.37,4.12)	2.49 (1.29,4.02)	2.70 (1.40,4.33)	0.83 (0.44,1.33)	1.95 (1.03,3.11)	3.19 (1.67,5.08)	4.01 (2.08,6.35)	1.80 (0.88,3.01)	3.01 (1.58,4.80)
Sudan	2.05 (0.98,3.73)	1.98 (0.95,3.67)	2.09 (0.99,3.89)	0.67 (0.32,1.24)	1.56 (0.75,2.85)	2.54 (1.21,4.61)	3.20 (1.53,5.83)	1.69 (0.79,3.12)	2.84 (1.38,5.20)
Afghanistan	5.30 (2.18,12.45)	4.97 (1.76,13.33)	5.36 (1.91,14.18)	5.64 (1.44,18.01)	5.15 (1.45,15.89)	4.79 (1.27,14.59)	4.44 (1.11,14.39)	4.54 (1.56,11.86)	8.26 (2.91,20.56)
Myanmar	0.19 (-2.88,3.21)	0.17 (-4.10,4.48)	0.20 (-4.08,4.49)	0.09 (-2.34,2.59)	0.14 (-4.78,5.08)	0.27 (-6.25,7.25)	0.17 (-6.46,6.93)	0.16 (-3.83,4.20)	0.24 (-4.43,4.97)

*Data are mean absolute difference in intakes (95% uncertainty interval) in 248 g servings per week. The standardized serving size used for this analysis is 248 g (8 oz) serving. Countries are ordered top to bottom from most to least populous based on 2018 youth (3-19 years) population data.

Supplementary Table 11. Global and regional mean (95% UI) sugar-sweetened beverage intakes (248 g serving/week) in youth (3-19 years) by age, sex, parental education, and area of residence across 185 countries in 1990.

	World	Central/Eastern Europe and Central Asia†	High-Income Countries	Latin America/ Caribbean	Middle East/ North Africa	South Asia†	Southeast and East Asia	Sub-Saharan Africa
Overall	2.8 (2.7-3.0)	3.3 (2.9-3.9)	4.9 (4.7-5.1)	9.2 (8.4-10.1)	6.1 (5.3-7.0)	0.9 (0.7-1.3)	1.4 (1.3-1.6)	2.1 (1.7-2.6)
Sex								
Girls	2.7 (2.6-2.9)	3.1 (2.7-3.7)	4.4 (4.2-4.7)	8.8 (8.0-9.8)	6.1 (5.3-7.1)	0.9 (0.6-1.3)	1.4 (1.2-1.5)	2.1 (1.7-2.5)
Boys	2.9 (2.7-3.1)	3.5 (3.0-4.1)	5.3 (5.0-5.5)	9.5 (8.6-10.6)	6.0 (5.2-7.0)	1.0 (0.7-1.3)	1.4 (1.2-1.6)	2.2 (1.7-2.7)
Age								
3-4	1.4 (1.2-1.5)	1.2 (0.9-1.6)	1.9 (1.8-2.0)	4.6 (4.1-5.2)	2.8 (2.4-3.3)	1.1 (0.8-1.6)	0.6 (0.5-0.7)	0.7 (0.5-0.9)
5-9	2.6 (2.4-2.7)	2.8 (2.3-3.5)	4.0 (3.8-4.2)	8.2 (7.4-9.1)	5.7 (4.9-6.6)	1.0 (0.7-1.4)	1.3 (1.1-1.5)	1.7 (1.3-2.1)
10-14	3.5 (3.3-3.7)	4.2 (3.6-5.0)	5.8 (5.5-6.1)	10.9 (9.9-12.1)	7.7 (6.7-8.9)	0.9 (0.6-1.2)	1.8 (1.6-2.0)	2.7 (2.2-3.4)
15-19	3.5 (3.3-3.7)	4.3 (3.7-5.1)	6.5 (6.2-6.8)	11.9 (10.9-13.1)	7.6 (6.6-8.7)	0.8 (0.6-1.1)	1.6 (1.5-1.8)	3.5 (2.9-4.2)
Parental education years								
0-6	2.2 (2.1-2.4)	3.2 (2.5-4.2)	4.5 (4.2-4.8)	8.5 (7.5-9.6)	6.3 (5.4-7.6)	0.7 (0.5-1.1)	1.0 (0.8-1.1)	1.5 (1.2-1.9)
>6-12	2.9 (2.7-3.1)	3.6 (3.0-4.3)	4.8 (4.6-5.1)	9.8 (8.7-10.9)	6.1 (5.4-7.1)	0.8 (0.6-1.2)	1.5 (1.3-1.7)	3.1 (2.5-3.9)
>12	3.9 (3.6-4.2)	3.1 (2.6-3.8)	5.0 (4.7-5.3)	9.7 (8.4-11.3)	5.1 (4.4-5.9)	3.2 (2.1-5.0)	2.0 (1.8-2.2)	4.3 (3.2-5.6)
Area of residency								
Rural	1.8 (1.7-2.0)	3.9 (3.2-4.8)	4.9 (4.7-5.1)	8.4 (7.5-9.4)	6.6 (5.5-8.0)	0.4 (0.3-0.6)	1.1 (1.0-1.2)	1.6 (1.3-2.0)
Urban	4.3 (4.0-4.6)	2.9 (2.5-3.4)	4.9 (4.6-5.1)	9.5 (8.6-10.5)	5.6 (4.9-6.4)	2.4 (1.6-3.6)	2.1 (1.8-2.3)	3.4 (2.7-4.3)

Data are the mean intakes (95% uncertainty interval) in 248 g servings per week. All intakes are reported adjusted to 5,439 kJ/day (1,300 kcal/day) for ages 3-5 years; 7,113 kJ/day (1,700 kcal/day) for ages 6-10 years; and 8,368 kJ/day (2,000 kcal/day)for ages 11–19 years. Data are based on a Bayesian model that incorporated up to 451 individual-level dietary surveys, and additional survey-level and country-level covariates, to estimate dietary consumption levels. SSBs were defined as any beverage with added sugars and ≥209 kJ (50 kcal) per 237 g serving, including commercial or homemade beverages, soft drinks, energy drinks, fruit drinks, punch, lemonade, and aguas frescas. This definition excludes 100% fruit and vegetable juices, non-caloric artificially sweetened drinks, and sweetened milk. The standardized serving size used for this analysis is 248 grams (8 oz) serving. Parental education level "Low" 0 to 6 years of education; "Medium" >6 years to 12 years of education; and "High" >12 years of education. † In prior GDD reports, the region Central/ Eastern Europe and Central Asia was referred as Former Soviet Union, and Southeast and East Asia was referred as Asia.
SSBs, sugar-sweetened beverages; UIs, uncertainty intervals.

Supplementary Table 12. Global and regional mean (95% UI) sugar-sweetened beverage intakes (248 g serving/week) in youth (3-19 years) by age, sex, parental education, and area of residence across 185 countries in 2005.

	World	Central/Eastern Europe and Central Asia†	High-Income Countries	Latin America/ Caribbean	Middle East/ North Africa	South Asia†	Southeast and East Asia	Sub-Saharan Africa
Overall	3.3 (3.1-3.6)	3.8 (3.4-4.4)	6.4 (6.1-6.7)	8.3 (7.5-9.1)	7.1 (6.1-8.5)	1.4 (1.0-1.9)	1.7 (1.6-2.0)	3.0 (2.4-3.8)
Sex								
Girls	3.2 (3.0-3.5)	3.6 (3.2-4.2)	5.8 (5.5-6.1)	7.9 (7.1-8.9)	7.1 (6.0-8.7)	1.4 (1.0-2.0)	1.7 (1.5-2.0)	2.9 (2.3-3.8)
Boys	3.4 (3.1-3.7)	4.0 (3.5-4.6)	6.9 (6.6-7.3)	8.6 (7.7-9.5)	7.1 (6.0-8.5)	1.4 (1.0-2.0)	1.8 (1.5-2.0)	3.1 (2.4-4.0)
Age								
3-4	1.7 (1.5-1.9)	1.2 (0.9-1.6)	2.4 (2.2-2.5)	4.1 (3.6-4.6)	3.2 (2.7-3.9)	1.6 (1.2-2.3)	0.8 (0.6-1.1)	1.0 (0.7-1.3)
5-9	2.9 (2.7-3.2)	3.0 (2.5-3.6)	5.1 (4.8-5.4)	7.3 (6.5-8.1)	6.6 (5.6-8.0)	1.5 (1.1-2.1)	1.7 (1.5-2.0)	2.3 (1.8-3.0)
10-14	3.9 (3.7-4.3)	4.5 (4.0-5.2)	7.6 (7.2-8.0)	9.7 (8.8-10.8)	8.8 (7.4-10.6)	1.3 (1.0-1.9)	2.2 (1.9-2.5)	3.8 (2.9-5.0)
15-19	4.0 (3.7-4.3)	4.8 (4.3-5.5)	8.5 (8.1-9.0)	10.4 (9.5-11.4)	8.3 (7.1-9.8)	1.2 (0.9-1.7)	1.9 (1.7-2.1)	4.8 (3.8-6.2)
Parental education years								
0-6	2.6 (2.3-2.9)	4.3 (3.3-5.5)	5.5 (5.2-5.9)	7.6 (6.8-8.7)	8.3 (6.7-10.9)	0.9 (0.6-1.4)	1.7 (1.4-2.0)	2.1 (1.6-2.9)
>6-12	3.3 (3.1-3.6)	4.0 (3.4-4.8)	6.2 (5.8-6.5)	8.3 (7.4-9.4)	7.0 (6.0-8.3)	1.5 (1.0-2.2)	1.6 (1.4-1.8)	4.0 (3.0-5.2)
>12	4.0 (3.7-4.3)	3.7 (3.3-4.3)	6.5 (6.2-6.9)	8.6 (7.4-10.0)	5.7 (4.8-6.7)	2.0 (1.3-3.0)	2.1 (1.8-2.4)	4.5 (3.3-6.1)
Area of residency								
Rural	2.2 (2.0-2.5)	3.9 (3.3-4.6)	6.3 (6.0-6.6)	7.7 (6.8-8.7)	8.1 (6.5-10.5)	0.6 (0.5-0.9)	1.6 (1.4-1.9)	2.4 (1.9-3.2)
Urban	4.5 (4.2-4.9)	3.8 (3.3-4.3)	6.4 (6.1-6.8)	8.4 (7.6-9.3)	6.5 (5.6-7.5)	3.2 (2.2-4.8)	1.9 (1.7-2.2)	4.1 (3.2-5.4)

Data are the mean intakes (95% uncertainty interval) in 248 g servings per week. All intakes are reported adjusted to 5,439 kJ/day (1,300 kcal/day) for ages 3-5 years; 7,113 kJ/day (1,700 kcal/day) for ages 6-10 years; and 8,368 kJ/day (2,000 kcal/day)for ages 11–19 years. Data are based on a Bayesian model that incorporated up to 451 individual-level dietary surveys, and additional survey-level and country-level covariates, to estimate dietary consumption levels. SSBs were defined as any beverage with added sugars and ≥209 kJ (50 kcal) per 237 g serving, including commercial or homemade beverages, soft drinks, energy drinks, fruit drinks, punch, lemonade, and aguas frescas. This definition excludes 100% fruit and vegetable juices, non-caloric artificially sweetened drinks, and sweetened milk. The standardized serving size used for this analysis is 248 grams (8 oz) serving. Parental education level "Low" 0 to 6 years of education; "Medium" >6 years to 12 years of education; and "High" >12 years of education. † In prior GDD reports, the region Central/ Eastern Europe and Central Asia was referred as Former Soviet Union, and Southeast and East Asia was referred as Asia.
SSBs, sugar-sweetened beverages; UIs, uncertainty intervals.

Supplementary Table 13. National mean (95% UI) sugar-sweetened beverage intakes (248 g servings/week) in youth (3-19 years) by sex, age, parental education, and area of residence in the 25 most populous countries in 1990.

	Sex		Age				Parental education			Area of residence	
	Girls	Boys	3-4 years	5-9 years	10-14 years	15-19 years	≤6 years	>6-12 years	>12 years	Rural	Urban
Country	*mean (95% UI)*	*mean (95% UI)*	*mean (95% UI)*	*mean (95% UI)*	*mean (95% UI)*	*mean (95% UI)*	*mean (95% UI)*	*mean (95% UI)*	*mean (95% UI)*	*mean (95% UI)*	*mean (95% UI)*
India	0.2 (0.1-0.3)	0.3 (0.2-0.4)	0.3 (0.2-0.4)	0.2 (0.2-0.4)	0.2 (0.1-0.3)	0.2 (0.1-0.3)	0.2 (0.1-0.3)	0.3 (0.2-0.5)	0.4 (0.3-0.7)	0.1 (0.1-0.2)	0.6 (0.3-0.9)
China	0.6 (0.4-0.7)	0.6 (0.4-0.8)	0.3 (0.2-0.4)	0.5 (0.4-0.7)	0.7 (0.6-0.9)	0.7 (0.6-0.9)	0.5 (0.4-0.7)	0.6 (0.5-0.8)	0.6 (0.5-0.8)	0.6 (0.5-0.7)	0.6 (0.5-0.8)
Nigeria	0.3 (0.2-0.5)	0.3 (0.2-0.5)	0.1 (0.1-0.2)	0.2 (0.1-0.4)	0.4 (0.2-0.7)	0.5 (0.3-0.9)	0.2 (0.1-0.4)	0.4 (0.2-0.7)	0.5 (0.3-0.8)	0.2 (0.1-0.4)	0.4 (0.2-0.7)
Pakistan	6.4 (4.2-10.1)	6.4 (4.2-9.9)	7.3 (4.7-11.3)	6.6 (4.5-10.1)	6.1 (4.1-9.3)	5.7 (3.9-8.6)	4.8 (3.0-7.6)	8.8 (5.5-13.6)	11.7 (7.1-19.7)	3.1 (2.0-4.6)	14.1 (9.0-22.4)
Indonesia	0.8 (0.6-1.1)	0.9 (0.6-1.2)	0.4 (0.3-0.5)	0.8 (0.6-1.0)	1.0 (0.8-1.3)	1.0 (0.7-1.3)	0.8 (0.6-1.0)	0.9 (0.7-1.2)	0.9 (0.7-1.2)	0.8 (0.6-1.1)	0.8 (0.6-1.1)
United States	6.1 (5.7-6.5)	7.3 (6.9-7.8)	2.5 (2.4-2.7)	5.5 (5.2-5.8)	8.2 (7.7-8.6)	9.2 (8.7-9.8)	8.6 (8.0-9.3)	8.5 (7.9-9.1)	6.2 (5.9-6.6)	7.0 (6.6-7.3)	6.6 (6.2-7.1)
Brazil	7.7 (6.6-9.0)	8.4 (7.2-9.9)	4.0 (3.4-4.7)	7.2 (6.3-8.3)	9.6 (8.4-11.1)	10.3 (9.1-11.8)	7.8 (6.7-9.0)	8.3 (7.2-9.7)	8.4 (7.1-10.0)	7.3 (6.2-8.6)	8.3 (7.3-9.5)
Bangladesh	0.3 (0.2-0.4)	0.3 (0.2-0.4)	0.3 (0.2-0.4)	0.3 (0.2-0.4)	0.3 (0.2-0.4)	0.2 (0.2-0.3)	0.2 (0.1-0.3)	0.4 (0.3-0.5)	0.5 (0.3-0.8)	0.2 (0.1-0.2)	0.7 (0.5-1.1)
Ethiopia	2.1 (1.5-2.9)	2.2 (1.6-3.1)	0.7 (0.6-1.0)	1.7 (1.3-2.4)	2.8 (2.1-3.9)	3.6 (2.6-4.8)	1.8 (1.3-2.4)	3.0 (2.1-4.2)	3.3 (2.3-4.9)	2.0 (1.5-2.8)	3.2 (2.3-4.4)
DR Congo	0.8 (0.3-2.3)	0.9 (0.3-2.6)	0.3 (0.1-0.8)	0.7 (0.3-2.0)	1.2 (0.4-3.2)	1.5 (0.5-4.0)	0.7 (0.3-2.1)	1.3 (0.4-3.5)	1.4 (0.5-3.8)	0.8 (0.3-2.1)	1.2 (0.4-3.3)
Mexico	9.6 (8.5-11.0)	10.9 (9.6-12.3)	5.1 (4.5-5.7)	9.1 (8.1-10.2)	12.2 (10.9-13.6)	13.1 (11.9-14.5)	9.8 (8.7-11.1)	10.5 (9.3-11.9)	10.6 (9.0-12.5)	9.3 (8.3-10.5)	10.6 (9.5-11.9)
Philippines	6.5 (5.5-7.7)	5.6 (4.5-7.0)	2.8 (2.1-3.6)	5.7 (4.7-6.8)	7.6 (6.5-8.9)	7.5 (6.5-8.7)	5.3 (4.5-6.2)	6.3 (5.3-7.5)	6.3 (5.3-7.5)	5.9 (5.0-7.0)	6.2 (5.3-7.3)
Egypt	4.8 (3.9-5.8)	4.0 (3.2-4.9)	2.0 (1.6-2.4)	4.1 (3.4-5.0)	5.6 (4.6-6.8)	5.5 (4.5-6.7)	4.4 (3.6-5.3)	4.7 (3.8-5.8)	3.9 (3.1-4.8)	4.6 (3.8-5.6)	4.0 (3.3-4.9)
Russia	1.7 (1.4-2.1)	2.0 (1.6-2.4)	0.6 (0.5-0.8)	1.6 (1.3-1.9)	2.4 (2.0-2.8)	2.5 (2.1-2.9)	1.8 (1.4-2.4)	2.0 (1.6-2.5)	1.8 (1.5-2.2)	2.0 (1.6-2.4)	1.8 (1.5-2.1)
Tanzania	0.3 (0.2-0.5)	0.3 (0.2-0.5)	0.1 (0.1-0.2)	0.2 (0.1-0.4)	0.4 (0.2-0.7)	0.5 (0.3-0.9)	0.2 (0.1-0.4)	0.4 (0.2-0.7)	0.4 (0.2-0.8)	0.3 (0.2-0.5)	0.4 (0.2-0.7)
Vietnam	2.2 (1.6-2.9)	2.2 (1.6-3.0)	1.0 (0.7-1.4)	2.1 (1.5-2.7)	2.7 (2.1-3.6)	2.7 (2.1-3.5)	2.0 (1.5-2.6)	2.4 (1.8-3.1)	2.4 (1.8-3.1)	2.2 (1.7-2.8)	2.3 (1.7-3.0)
Turkey	4.7 (3.6-6.3)	4.8 (3.7-6.4)	2.1 (1.6-2.8)	4.4 (3.4-5.7)	5.9 (4.6-7.7)	5.8 (4.6-7.6)	4.7 (3.6-6.2)	5.1 (3.9-6.6)	4.1 (3.2-5.4)	5.2 (4.0-6.8)	4.5 (3.5-5.8)
Kenya	0.9 (0.5-1.5)	1.1 (0.6-1.8)	0.3 (0.2-0.5)	0.8 (0.5-1.3)	1.3 (0.8-2.1)	1.6 (1.0-2.6)	0.8 (0.5-1.2)	1.3 (0.8-2.2)	1.4 (0.8-2.5)	0.9 (0.5-1.5)	1.4 (0.8-2.4)
Iran	3.9 (3.3-4.6)	4.1 (3.5-4.8)	1.8 (1.5-2.1)	3.8 (3.2-4.4)	5.1 (4.4-5.9)	5.0 (4.4-5.7)	4.0 (3.4-4.7)	4.3 (3.7-5.1)	3.5 (3.0-4.2)	4.3 (3.6-5.1)	3.7 (3.2-4.3)
Uganda	0.1 (0.1-0.2)	0.1 (0.1-0.2)	0.0 (0.0-0.1)	0.1 (0.0-0.2)	0.1 (0.1-0.3)	0.2 (0.1-0.3)	0.1 (0.0-0.2)	0.2 (0.1-0.3)	0.2 (0.1-0.3)	0.1 (0.1-0.2)	0.2 (0.1-0.3)
Japan	5.3 (4.4-6.4)	6.1 (5.1-7.4)	2.5 (1.9-3.3)	5.1 (4.2-6.1)	6.8 (5.8-8.0)	6.7 (5.7-7.8)	4.9 (4.1-5.8)	5.8 (4.9-6.9)	5.8 (4.9-6.8)	5.6 (4.7-6.6)	5.8 (4.9-6.8)
South Africa	7.0 (5.2-9.4)	7.6 (5.6-10.2)	2.4 (1.8-3.2)	5.7 (4.3-7.4)	9.2 (7.0-12.2)	11.6 (8.9-15.1)	5.0 (3.7-6.9)	8.5 (6.3-11.4)	9.5 (7.0-12.8)	5.6 (4.2-7.5)	8.9 (6.7-11.8)
Sudan	3.9 (2.5-5.9)	4.1 (2.6-6.2)	1.3 (0.9-2.0)	3.1 (2.1-4.7)	5.1 (3.4-7.6)	6.4 (4.3-9.5)	3.3 (2.2-4.9)	5.5 (3.5-8.5)	6.1 (3.8-9.8)	3.4 (2.2-5.1)	5.4 (3.5-8.2)
Afghanistan	0.2 (0.1-0.5)	0.2 (0.1-0.5)	0.2 (0.1-0.6)	0.2 (0.1-0.5)	0.2 (0.1-0.5)	0.2 (0.1-0.4)	0.2 (0.1-0.4)	0.4 (0.2-0.7)	0.5 (0.2-1.1)	0.1 (0.1-0.3)	0.6 (0.3-1.3)
Myanmar	0.7 (0.4-1.2)	0.8 (0.5-1.3)	0.3 (0.2-0.6)	0.7 (0.4-1.3)	0.9 (0.5-1.7)	0.9 (0.5-1.7)	0.7 (0.4-1.2)	0.8 (0.5-1.4)	0.8 (0.5-1.4)	0.7 (0.5-1.2)	0.8 (0.5-1.3)

*Data are mean absolute difference in intakes (95% uncertainty interval) in 248 g servings per week. The standardized serving size used for this analysis is 248 g (8 oz) serving. Countries are ordered top to bottom from most to least populous based on 2018 youth (3-19 years) population data.

Supplementary Table 14. National mean (95% UI) sugar-sweetened beverage intakes (248 g servings/week) in youth (3-19 years) by sex, age, parental education, and area of residence in the 25 most populous countries in 2005.

	Sex		Age				Parental education			Area of residence	
	Girls	Boys	3-4 years	5-9 years	10-14 years	15-19 years	≤6 years	>6-12 years	>12 years	Rural	Urban
Country	*mean (95% UI)*	*mean (95% UI)*	*mean (95% UI)*	*mean (95% UI)*	*mean (95% UI)*	*mean (95% UI)*	*mean (95% UI)*	*mean (95% UI)*	*mean (95% UI)*	*mean (95% UI)*	*mean (95% UI)*
India	0.2 (0.2-0.4)	0.3 (0.2-0.5)	0.3 (0.2-0.5)	0.3 (0.2-0.5)	0.3 (0.2-0.4)	0.3 (0.2-0.4)	0.2 (0.1-0.3)	0.3 (0.2-0.5)	0.4 (0.3-0.7)	0.1 (0.1-0.2)	0.7 (0.4-1.1)
China	0.5 (0.4-0.7)	0.5 (0.4-0.7)	0.2 (0.2-0.3)	0.5 (0.4-0.6)	0.6 (0.5-0.8)	0.6 (0.5-0.8)	0.5 (0.4-0.6)	0.5 (0.4-0.7)	0.5 (0.4-0.7)	0.5 (0.4-0.7)	0.5 (0.4-0.7)
Nigeria	2.8 (1.8-4.5)	3.0 (1.8-4.8)	1.0 (0.6-1.7)	2.3 (1.3-3.9)	3.7 (2.2-6.4)	4.6 (2.7-7.9)	2.3 (1.4-3.8)	3.8 (2.3-6.4)	4.3 (2.5-7.3)	2.3 (1.5-3.8)	3.7 (2.4-6.2)
Pakistan	7.9 (5.1-12.4)	7.9 (5.2-12.2)	9.0 (5.8-14.0)	8.2 (5.5-12.5)	7.6 (5.1-11.5)	7.0 (4.7-10.7)	4.9 (3.1-7.9)	9.1 (5.7-14.3)	12.2 (7.4-20.5)	3.5 (2.3-5.4)	16.3 (10.5-25.7)
Indonesia	1.2 (0.9-1.7)	1.4 (1.0-1.9)	0.6 (0.4-0.8)	1.2 (0.9-1.6)	1.6 (1.2-2.1)	1.6 (1.2-2.1)	1.2 (0.9-1.5)	1.4 (1.0-1.9)	1.4 (1.0-1.8)	1.3 (1.0-1.7)	1.3 (1.0-1.8)
United States	8.7 (8.1-9.3)	10.4 (9.8-11.1)	3.6 (3.3-3.8)	7.7 (7.2-8.2)	11.4 (10.7-12.1)	12.9 (12.2-13.7)	12.6 (11.7-13.6)	12.4 (11.5-13.3)	9.1 (8.6-9.7)	9.9 (9.4-10.4)	9.5 (8.9-10.1)
Brazil	4.8 (4.1-5.6)	5.3 (4.5-6.2)	2.5 (2.1-2.9)	4.4 (3.8-5.1)	5.9 (5.2-6.7)	6.3 (5.6-7.2)	4.8 (4.1-5.6)	5.1 (4.4-5.9)	5.2 (4.3-6.1)	4.5 (3.9-5.3)	5.2 (4.5-5.9)
Bangladesh	0.4 (0.3-0.6)	0.4 (0.3-0.6)	0.5 (0.4-0.6)	0.4 (0.3-0.6)	0.4 (0.3-0.6)	0.4 (0.3-0.5)	0.2 (0.2-0.4)	0.4 (0.3-0.7)	0.6 (0.4-1.0)	0.2 (0.1-0.3)	1.0 (0.6-1.5)
Ethiopia	3.4 (2.5-4.6)	3.6 (2.6-4.9)	1.2 (0.9-1.5)	2.8 (2.0-3.7)	4.5 (3.3-6.2)	5.7 (4.2-7.6)	2.7 (2.0-3.6)	4.5 (3.2-6.2)	5.0 (3.4-7.3)	3.2 (2.4-4.3)	5.1 (3.8-6.9)
DR Congo	1.1 (0.4-2.9)	1.2 (0.4-3.3)	0.4 (0.1-1.0)	0.9 (0.3-2.4)	1.5 (0.5-4.0)	1.8 (0.7-5.1)	0.9 (0.3-2.5)	1.5 (0.6-4.2)	1.7 (0.6-4.6)	0.9 (0.3-2.5)	1.5 (0.5-4.0)
Mexico	9.5 (8.3-10.9)	10.7 (9.4-12.2)	5.0 (4.4-5.6)	8.9 (7.9-10.0)	11.9 (10.6-13.4)	12.8 (11.5-14.2)	9.5 (8.4-10.8)	10.2 (9.0-11.6)	10.3 (8.8-12.2)	9.1 (8.1-10.4)	10.4 (9.2-11.7)
Philippines	6.3 (5.3-7.4)	5.4 (4.3-6.7)	2.6 (2.0-3.5)	5.4 (4.5-6.5)	7.3 (6.2-8.5)	7.1 (6.1-8.2)	5.0 (4.2-5.9)	6.0 (5.0-7.1)	6.0 (5.0-7.1)	5.7 (4.8-6.7)	6.0 (5.1-7.0)
Egypt	4.6 (3.7-5.6)	3.8 (3.0-4.7)	1.8 (1.5-2.2)	3.8 (3.1-4.6)	5.1 (4.2-6.2)	5.1 (4.1-6.2)	4.3 (3.5-5.2)	4.6 (3.7-5.6)	3.8 (3.0-4.7)	4.4 (3.6-5.4)	3.8 (3.1-4.7)
Russia	4.3 (3.5-5.2)	4.8 (3.9-5.8)	1.4 (1.0-1.9)	3.6 (3.0-4.3)	5.4 (4.5-6.3)	5.5 (4.7-6.5)	4.4 (3.4-5.9)	4.9 (3.9-6.0)	4.5 (3.8-5.3)	4.8 (4.0-5.9)	4.4 (3.7-5.2)
Tanzania	2.8 (1.7-5.0)	3.0 (1.8-5.1)	1.0 (0.5-1.8)	2.3 (1.2-4.2)	3.7 (2.1-6.8)	4.6 (2.6-8.8)	2.1 (1.3-3.6)	3.6 (2.1-6.1)	4.0 (2.3-6.9)	2.6 (1.6-4.3)	4.1 (2.4-6.9)
Vietnam	3.4 (2.6-4.4)	3.4 (2.6-4.5)	1.5 (1.1-2.0)	3.0 (2.4-3.9)	4.1 (3.2-5.1)	4.0 (3.2-5.0)	3.0 (2.4-3.8)	3.6 (2.8-4.5)	3.6 (2.8-4.6)	3.4 (2.7-4.3)	3.5 (2.8-4.5)
Turkey	3.3 (2.5-4.4)	3.4 (2.6-4.5)	1.5 (1.1-1.9)	3.0 (2.4-4.0)	4.1 (3.2-5.3)	4.1 (3.2-5.3)	3.3 (2.5-4.4)	3.6 (2.8-4.6)	2.9 (2.2-3.8)	3.7 (2.8-4.8)	3.2 (2.5-4.1)
Kenya	0.7 (0.4-1.2)	0.9 (0.5-1.5)	0.3 (0.2-0.4)	0.6 (0.4-1.0)	1.0 (0.6-1.7)	1.3 (0.8-2.1)	0.6 (0.3-0.9)	1.0 (0.6-1.6)	1.1 (0.6-1.9)	0.7 (0.4-1.2)	1.1 (0.7-1.9)
Iran	4.2 (3.6-4.9)	4.4 (3.7-5.1)	1.8 (1.5-2.1)	3.7 (3.2-4.3)	5.0 (4.4-5.8)	5.0 (4.4-5.7)	4.4 (3.8-5.1)	4.8 (4.1-5.5)	3.9 (3.3-4.6)	4.7 (4.0-5.5)	4.1 (3.6-4.7)
Uganda	2.4 (1.5-4.1)	2.5 (1.5-4.2)	0.8 (0.5-1.5)	2.0 (1.1-3.5)	3.2 (1.8-5.9)	4.0 (2.2-7.2)	2.0 (1.1-3.3)	3.3 (2.0-5.7)	3.6 (2.0-6.6)	2.3 (1.4-3.7)	3.6 (2.2-5.8)
Japan	4.9 (4.0-5.9)	5.7 (4.7-6.8)	2.3 (1.7-3.1)	4.8 (4.0-5.7)	6.4 (5.4-7.5)	6.3 (5.3-7.3)	4.5 (3.7-5.3)	5.3 (4.5-6.3)	5.3 (4.5-6.3)	5.1 (4.3-6.1)	5.3 (4.5-6.3)
South Africa	5.1 (3.8-6.8)	5.5 (4.1-7.4)	1.6 (1.2-2.1)	3.8 (2.9-5.0)	6.2 (4.7-8.2)	7.8 (6.0-10.2)	3.3 (2.4-4.6)	5.6 (4.2-7.5)	6.2 (4.6-8.4)	3.9 (2.9-5.2)	6.2 (4.7-8.2)
Sudan	4.2 (2.8-6.5)	4.4 (2.9-6.8)	1.4 (0.9-2.3)	3.4 (2.2-5.1)	5.6 (3.7-8.3)	7.0 (4.7-10.4)	3.3 (2.2-5.0)	5.6 (3.6-8.7)	6.3 (3.9-10.0)	3.6 (2.4-5.5)	5.8 (3.8-8.8)
Afghanistan	1.4 (0.7-2.8)	1.5 (0.8-3.0)	1.6 (0.8-3.4)	1.5 (0.7-3.3)	1.3 (0.7-2.9)	1.3 (0.6-2.6)	1.1 (0.5-2.3)	2.0 (0.9-4.2)	2.6 (1.2-5.7)	0.8 (0.4-1.5)	3.6 (1.8-7.8)
Myanmar	3.5 (2.3-5.6)	3.7 (2.5-6.1)	1.6 (0.9-2.9)	3.3 (1.9-5.9)	4.4 (2.7-7.7)	4.3 (2.6-7.4)	3.3 (2.1-5.5)	4.0 (2.5-6.6)	3.9 (2.5-6.5)	3.6 (2.4-5.7)	3.7 (2.4-6.0)

*Data are mean absolute difference in intakes (95% uncertainty interval) in 248 g servings per week. The standardized serving size used for this analysis is 248 g (8 oz) serving. Countries are ordered top to bottom from most to least populous based on 2018 youth (3-19 years) population data.

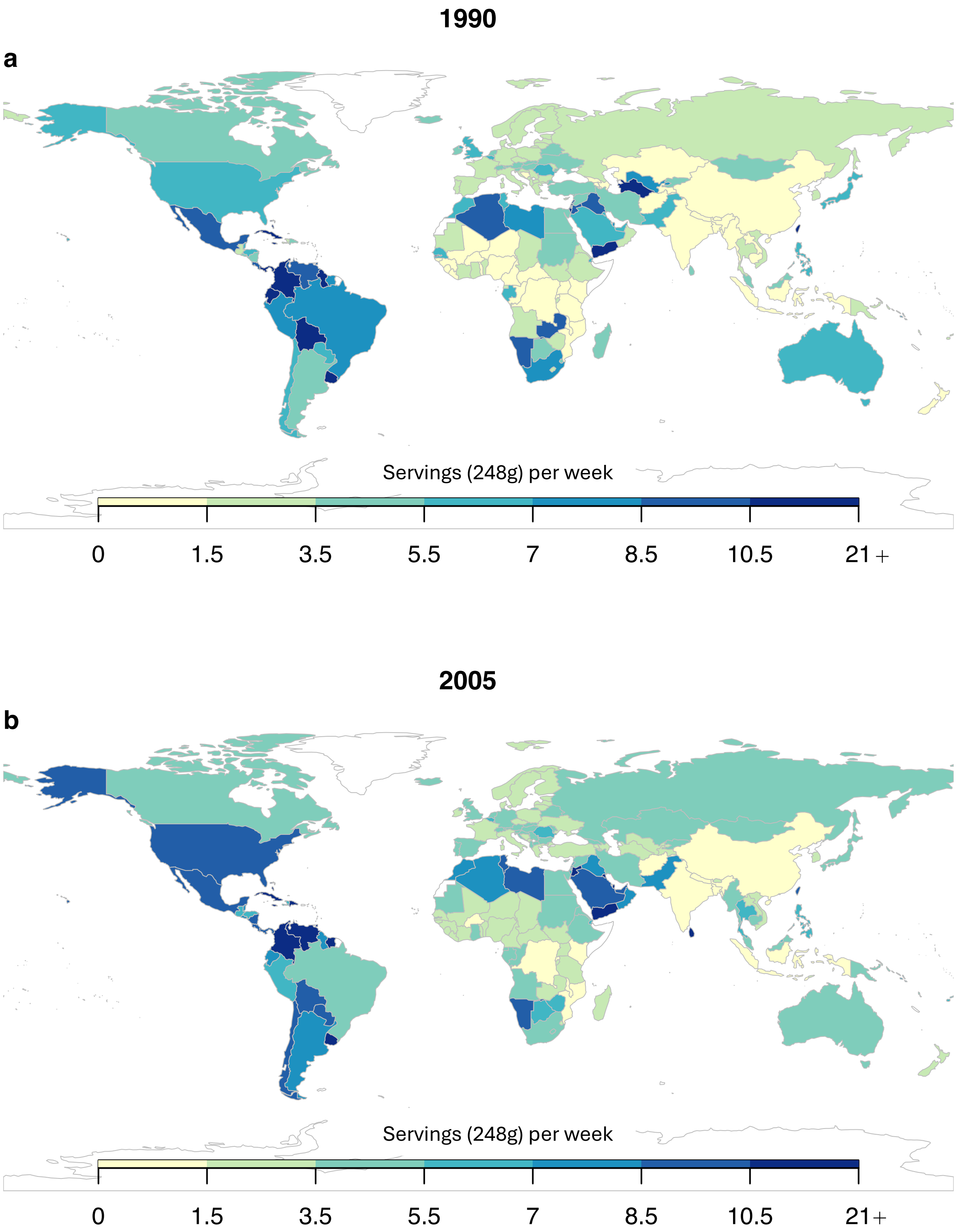

Supplementary Figure 5. National mean sugar-sweetened beverage intakes (248 g servings/week) in youth (3-19 years) across 185 countries in 1990 (a) and 2005 (b). SSBs were defined as any beverage with added sugars and ≥209 kJ (50 kcal) per 237 g serving, including commercial or homemade beverages, soft drinks, energy drinks, fruit drinks, punch, lemonade, and aguas frescas. This definition excludes 100% fruit and vegetable juices, non-caloric artificially sweetened drinks, and sweetened milk. The standardized serving size used for this analysis is 248 grams (8 oz) serving. For this visual representation, values were truncated at 21 servings/week to better reflect the distribution of intakes globally. The analysis of the data was done using the rworldmap package (v1.3-6).

SSBs, sugar-sweetened beverages.

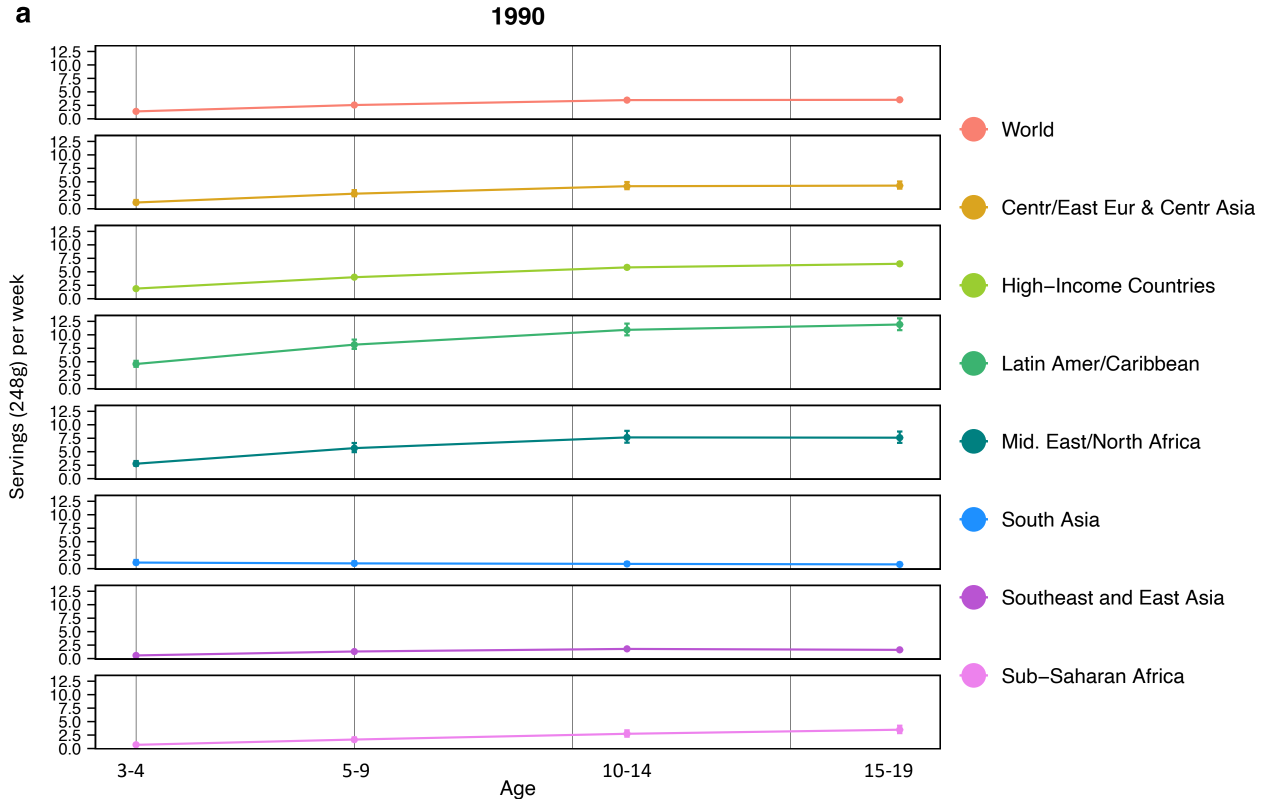

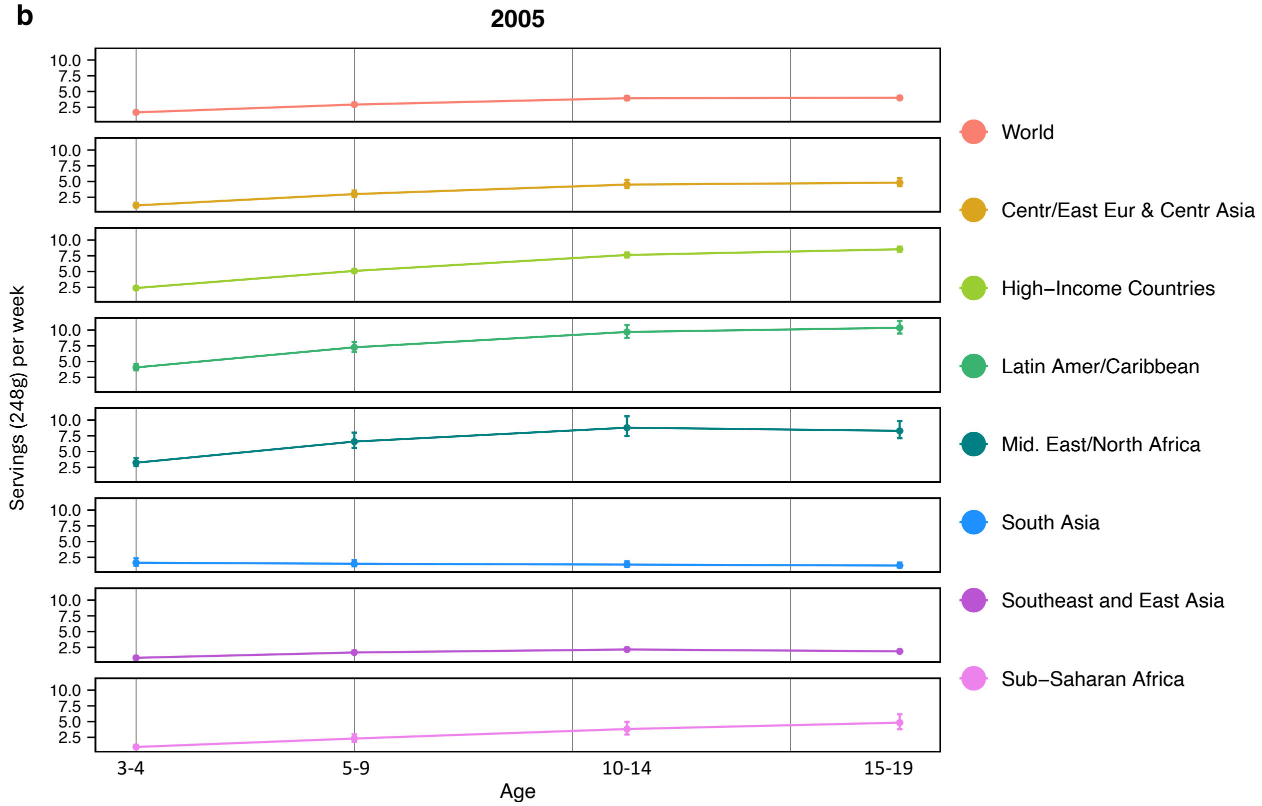

Supplementary Figure 6. Global and regional sugar-sweetened beverage intakes (248 g servings/week) by age in youth (3-19 years) in 1990 and 2005. SSBs were defined as any beverage with added sugars and ≥209 kJ (50 kcal) per 237 g serving, including commercial or homemade beverages, soft drinks, energy drinks, fruit drinks, punch, lemonade, and aguas frescas. This definition excludes 100% fruit and vegetable juices, non-caloric artificially sweetened drinks, and sweetened milk. The standardized serving size used for this analysis is 248 grams (8 oz) serving. The filled circles represent the mean SSBs intake (248 g serving/week) and the error bars the 95% UIs. Age groups are 3-4, 5-9, 10-14, and 15-19 years. In prior GDD reports, the region Central or Eastern Europe and Central Asia was referred as Former Soviet Union, and Southeast and East Asia was referred as Asia.

GDD, Global Dietary Database; SSBs, sugar-sweetened beverages; UIs, uncertainty intervals.

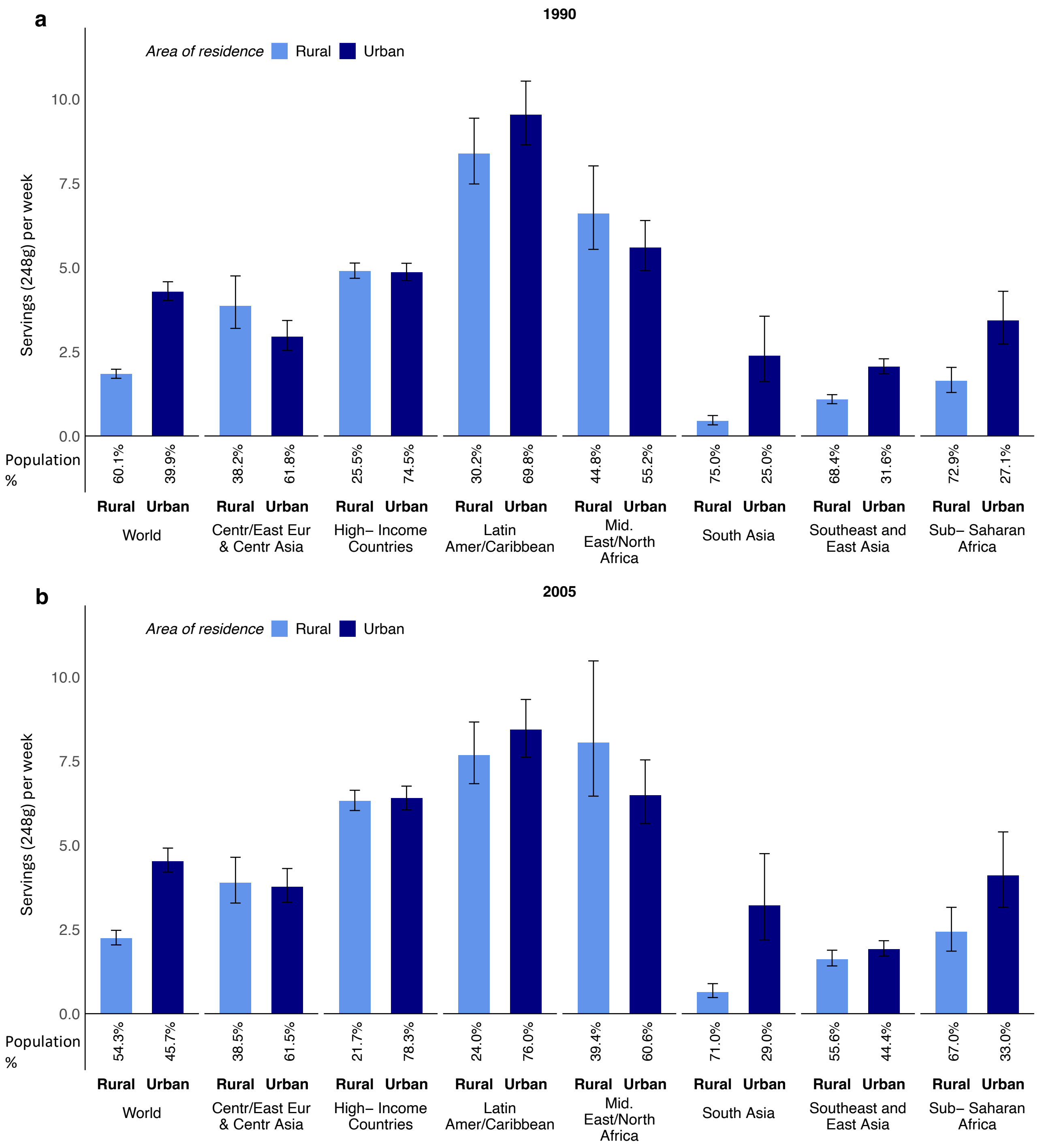

Supplementary Figure 7. Global and regional sugar-sweetened beverage intakes (248 g servings/week) in youth (3-19 years) by area of residence in 1990 and 2005. SSBs were defined as any beverage with added sugars and ≥209 kJ (50 kcal) per 237 g serving, including commercial or homemade beverages, soft drinks, energy drinks, fruit drinks, punch, lemonade, and aguas frescas. This definition excludes 100% fruit and vegetable juices, non-caloric artificially sweetened drinks, and sweetened milk. The standardized serving size used for this analysis is 248 grams (8 oz) serving. The filled bars represent the mean SSBs intake (248 g servings/week) and the error bars the 95% UIs. Values were truncated at 11.5 servings to better reflect the distribution of intakes. Upper 95% UIs above that value are shown with a dashed line. The values below the bars correspond to the percentage (%) of the global population represented in that strata. Colors represent the area of residence as "rural" (light blue) or "urban" (dark blue). In prior GDD reports, the region Central or Eastern Europe and Central Asia was referred as Former Soviet Union, and Southeast and East Asia was referred as Asia.

GDD, Global Dietary Database; SSBs, sugar-sweetened beverages; UIs, uncertainty intervals.

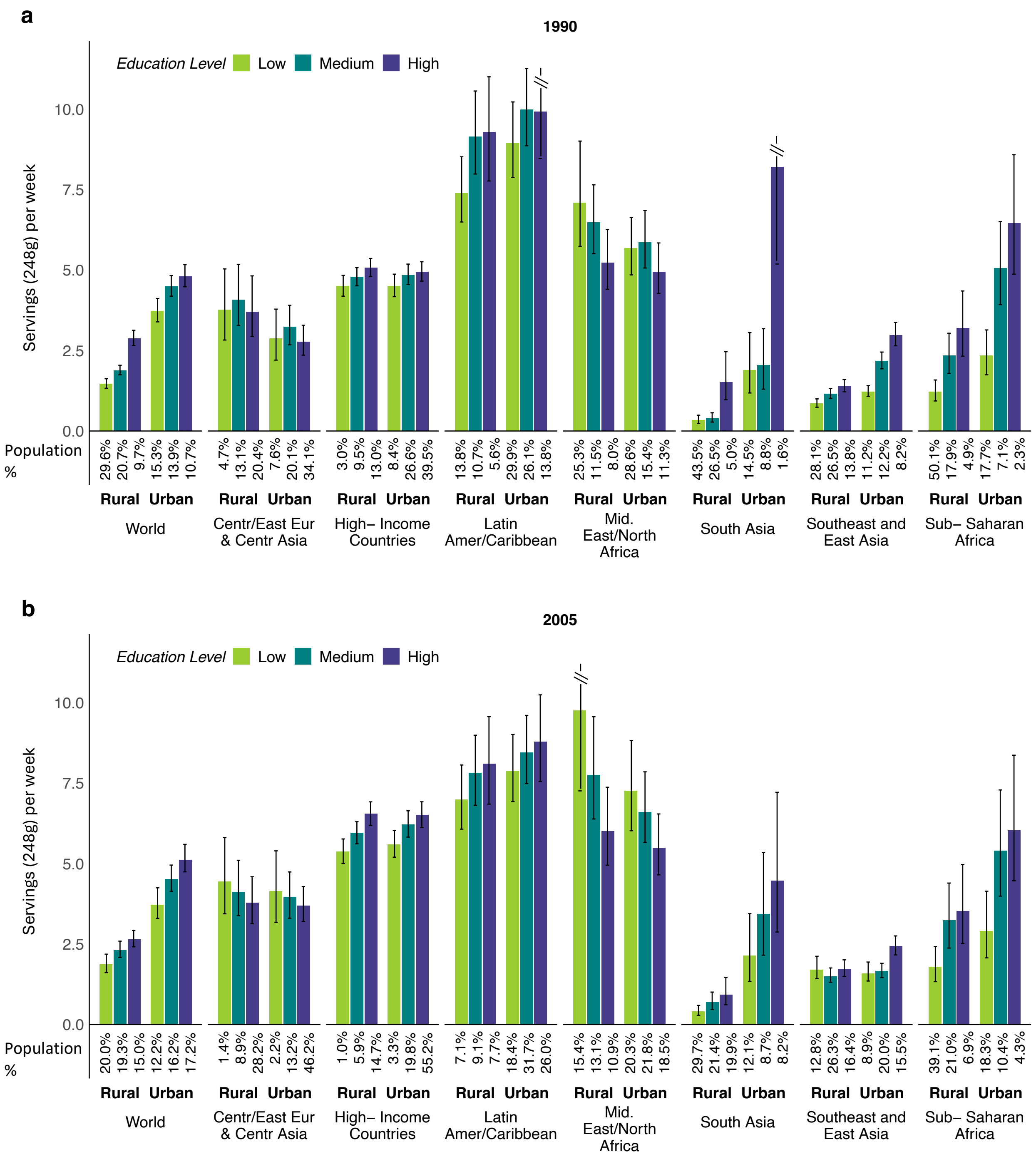

Supplementary Figure 8. Global and regional sugar-sweetened beverage intakes (248 g servings/week) in youth (3-19 years) by education and area of residence in 1990 and 2005. SSBs were defined as any beverage with added sugars and ≥209 kJ (50 kcal) per 237 g serving, including commercial or homemade beverages, soft drinks, energy drinks, fruit drinks, punch, lemonade, and aguas frescas. This definition excludes 100% fruit and vegetable juices, non-caloric artificially sweetened drinks, and sweetened milk. The standardized serving size used for this analysis is 248 grams (8 oz) serving. The filled bars represent the mean SSBs intake (248 g servings/week) and the error bars the 95% UIs. Values were truncated at 11.5 servings/week to better reflect the distribution of intakes. Upper 95% UIs above that value are shown with a dashed line. The values below the bars correspond to the percentage (%) of the global population represented in that strata. Colors represent the education level as "low" 0 to 6 years of education (light green), "medium" >6 years to 12 years of education (dark green), or "high" >12 years of education (purple). In prior GDD reports, the region Central or Eastern Europe and Central Asia was referred as Former Soviet Union, and Southeast and East Asia was referred as Asia.

GDD, Global Dietary Database; SSBs, sugar-sweetened beverages; UIs, uncertainty intervals.

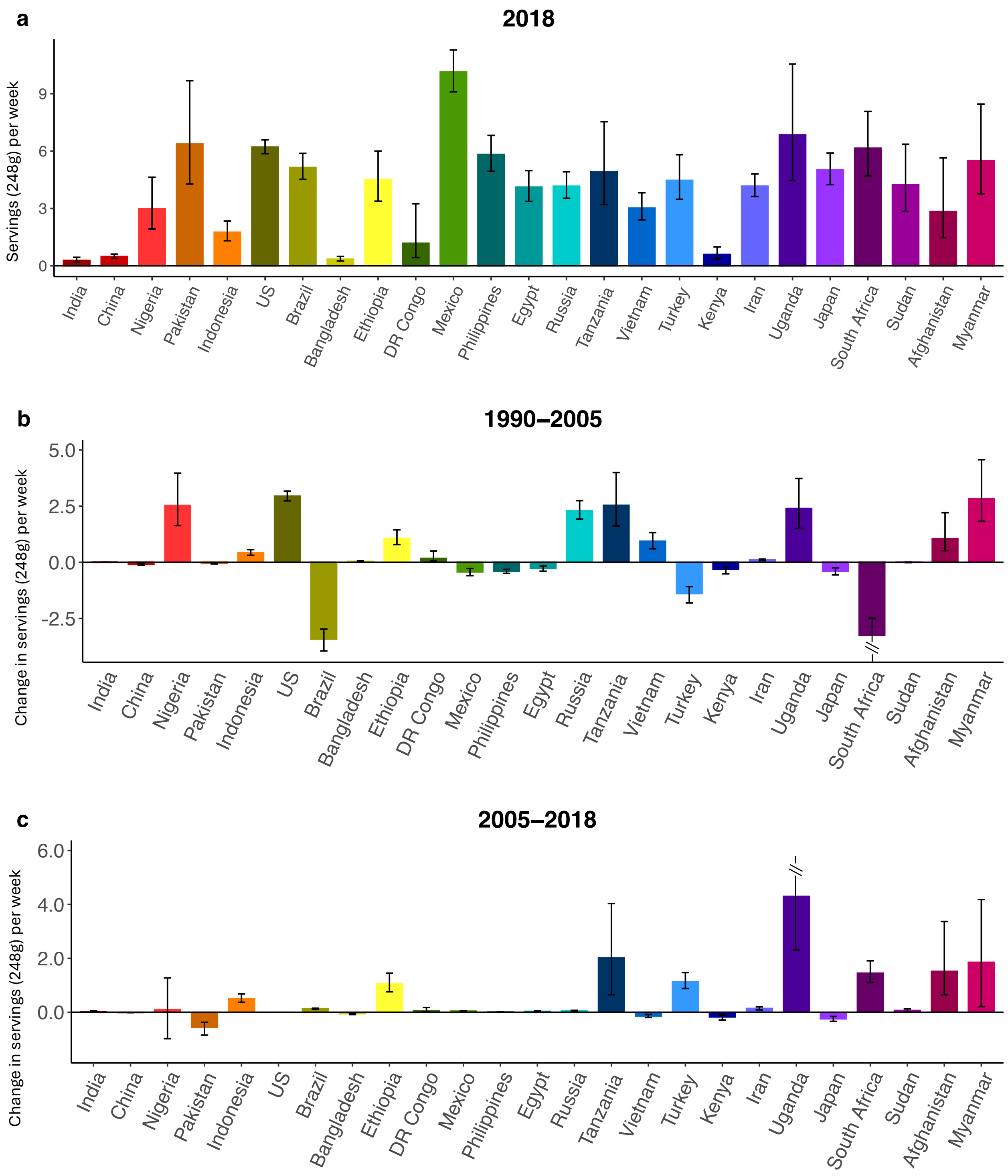

Supplementary Figure 9. Mean sugar-sweetened beverage intakes (248 g servings/week) in youth (3-19+ years) in the 25 most populous countries in 2018 and absolute change from 1990 to 2005 and from 2005 to 2018. SSBs were defined as any beverage with added sugars and ≥209 kJ (50 kcal) per 237 g serving, including commercial or homemade beverages, soft drinks, energy drinks, fruit drinks, punch, lemonade, and aguas frescas. This definition excludes 100% fruit and vegetable juices, non-caloric artificially sweetened drinks, and sweetened milk. The standardized serving size used for this analysis is 248 grams (8 oz) serving. The filled bars represent the mean SSB intakes (248 g serving/week) and the error bars the 95% UIs. Values were truncated from -4.0 to 5.0 servings for the mid panel (b) and at -1.5 to 6.0 for the bottom panel (c). Upper 95% UIs above those value are shown with a dashed line. Countries are ordered left to right from most to least populous based on 2018 youth (3-19 years) population data.

SSBs, sugar-sweetened beverages; UIs, uncertainty intervals.

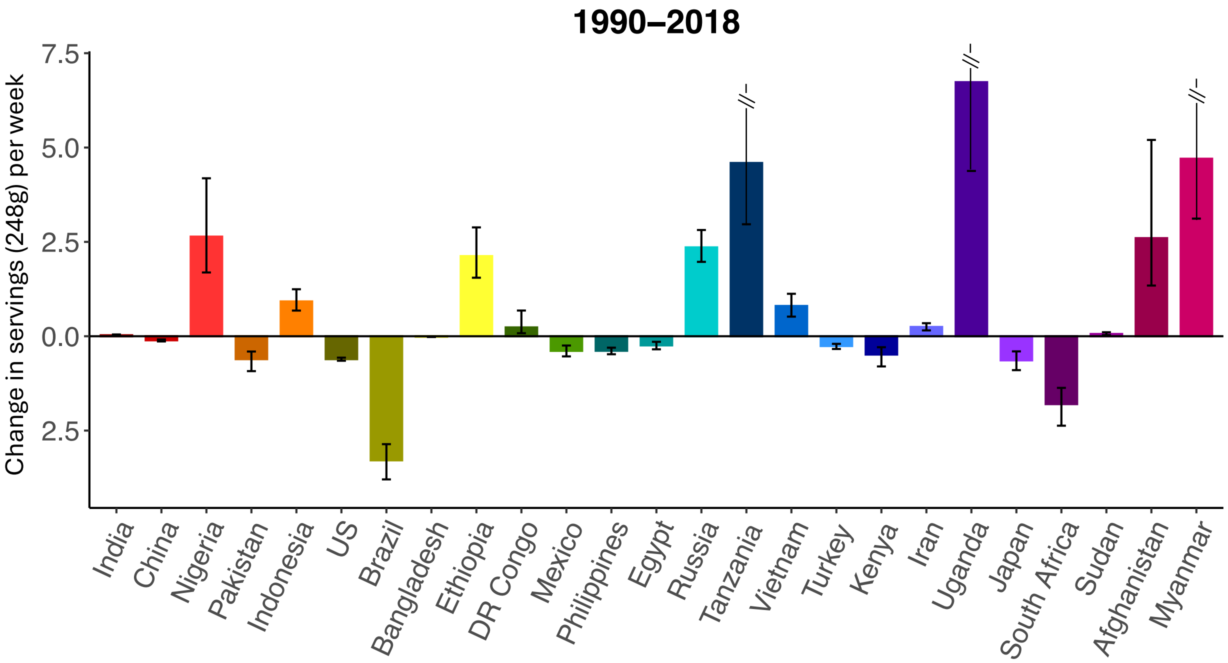

Supplementary Figure 10. Absolute change in mean sugar-sweetened beverage intakes (248 g servings/week) in youth (3-19 years) in the 25 most populous countries from 1990 to 2018. SSBs were defined as any beverage with added sugars and ≥209 kJ (50 kcal) per 237 g serving, including commercial or homemade beverages, soft drinks, energy drinks, fruit drinks, punch, lemonade, and aguas frescas. This definition excludes 100% fruit and vegetable juices, non-caloric artificially sweetened drinks, and sweetened milk. The standardized serving size used for this analysis is 248 grams (8 oz) serving. The filled bars represent the mean SSB intakes (248 g serving/week) and the error bars the 95% UIs. Values were truncated at -4.0 to 7.0 serving. Upper 95% UIs above that value are shown with a dashed line. Countries are ordered left to right from most to least populous based on 2018 youth (3-19 years) population data.

SSBs, sugar-sweetened beverages; UIs, uncertainty intervals.

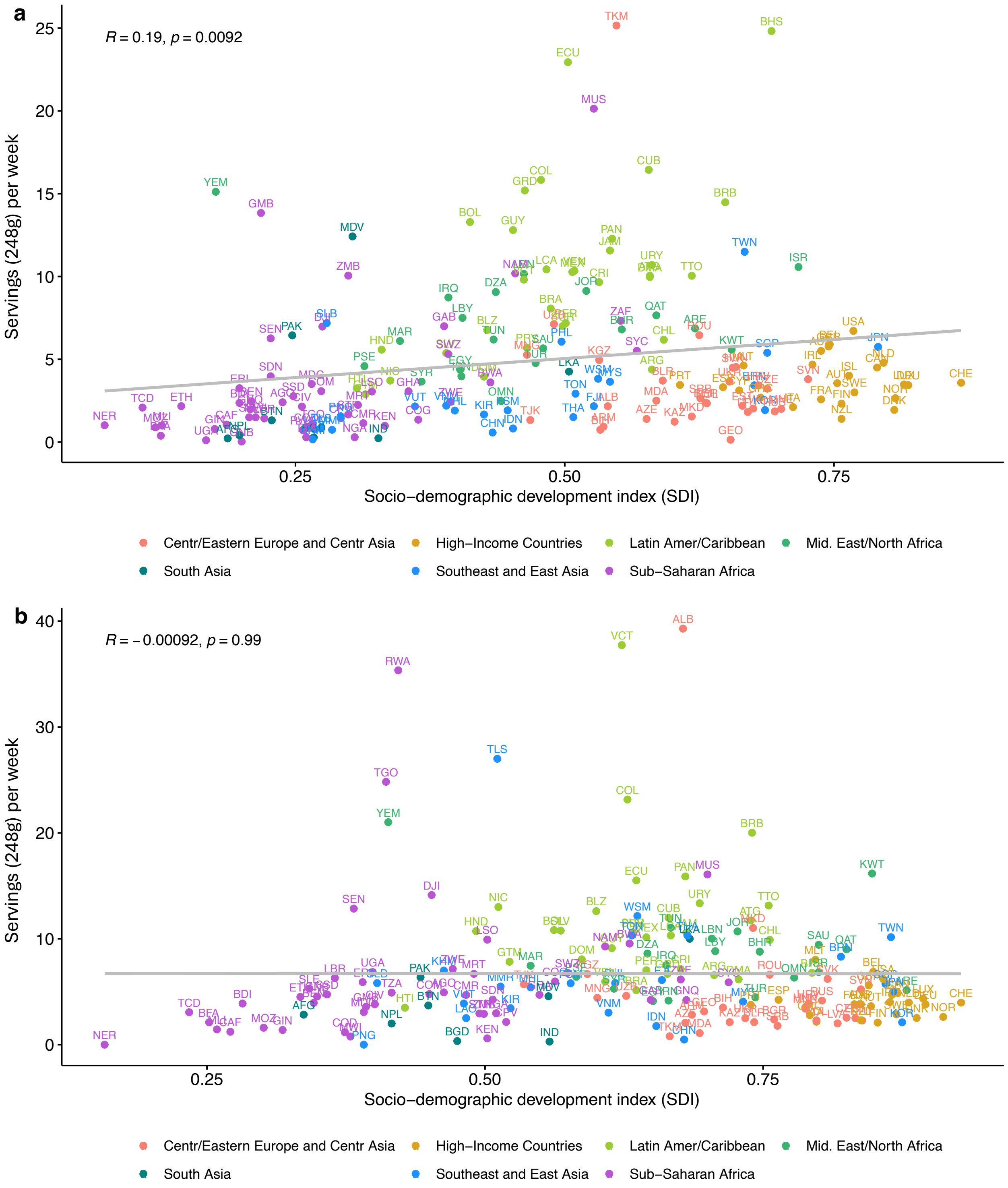

Supplementary Figure 11. National correlation of sugar-sweetened beverage intake (248 g servings/week) in youth (3-19 years) and socio-demographic development index by world region in 1990 and 2018 for 185 countries. (a) National correlation of SSB intakes and SDI by world region in 1990; **(b)** national correlation of SSB intakes and SDI by world region in 2018. Spearman correlation was assessed between SDI and SSB intakes among a total of 185 countries were included in this analysis. SSBs were defined as any beverage with added sugars and ≥209 kJ (50 kcal) per 237 g serving, including commercial or homemade beverages, soft drinks, energy drinks, fruit drinks, punch, lemonade, and aguas frescas. This definition excludes 100% fruit and vegetable juices, non-caloric artificially sweetened drinks, and sweetened milk. The standardized serving size used for this analysis is 248 grams (8 oz) serving. SDI was obtained from the Global Burden of Diseases study.

Centr/East Eur & Centr Asia, Central/Eastern Europe and Central Asia; GDD, Global Dietary Database; Latin Amer/Caribbean, Latin America/Caribbean; SDI, sociodemographic development index; SSBs, sugar-sweetened beverages; UIs, uncertainty intervals.

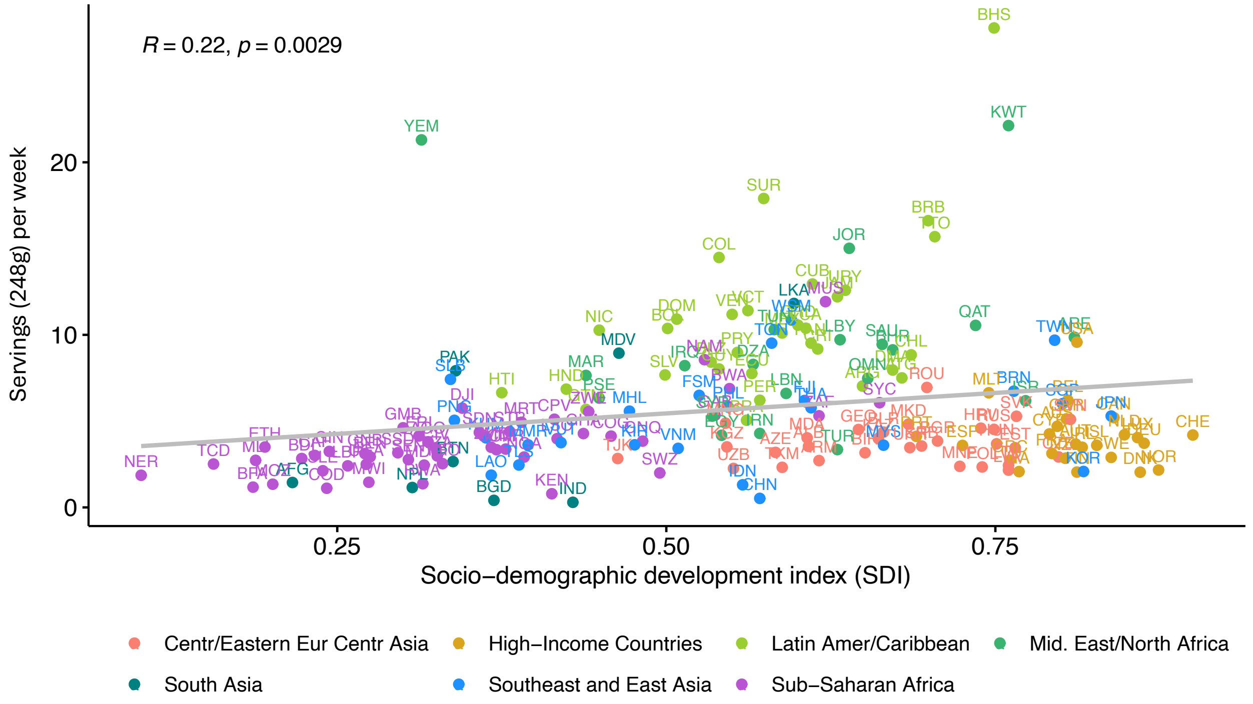

Supplementary Figure 12. National correlation of sugar-sweetened beverage intake (248 g servings/week) in youth (3-19 years) and socio-demographic development index by world region in 2005 for 185 countries. Spearman correlation was assessed between SDI and SSB intakes among a total of 185 countries that were included in this analysis. SSBs were defined as any beverage with added sugars and ≥209 kJ (50 kcal) per 237 g serving, including commercial or homemade beverages, soft drinks, energy drinks, fruit drinks, punch, lemonade, and aguas frescas. This definition excludes 100% fruit and vegetable juices, non-caloric artificially sweetened drinks, and sweetened milk. The standardized serving size used for this analysis is 248 grams (8 oz) serving. SDI was obtained from the Global Burden of Diseases study.

Centr/East Eur & Centr Asia, Central/Eastern Europe and Central Asia; GDD, Global Dietary Database; Latin Amer/Caribbean, Latin America/Caribbean; SDI, sociodemographic development index; SSBs, sugar-sweetened beverages; UIs, uncertainty intervals.

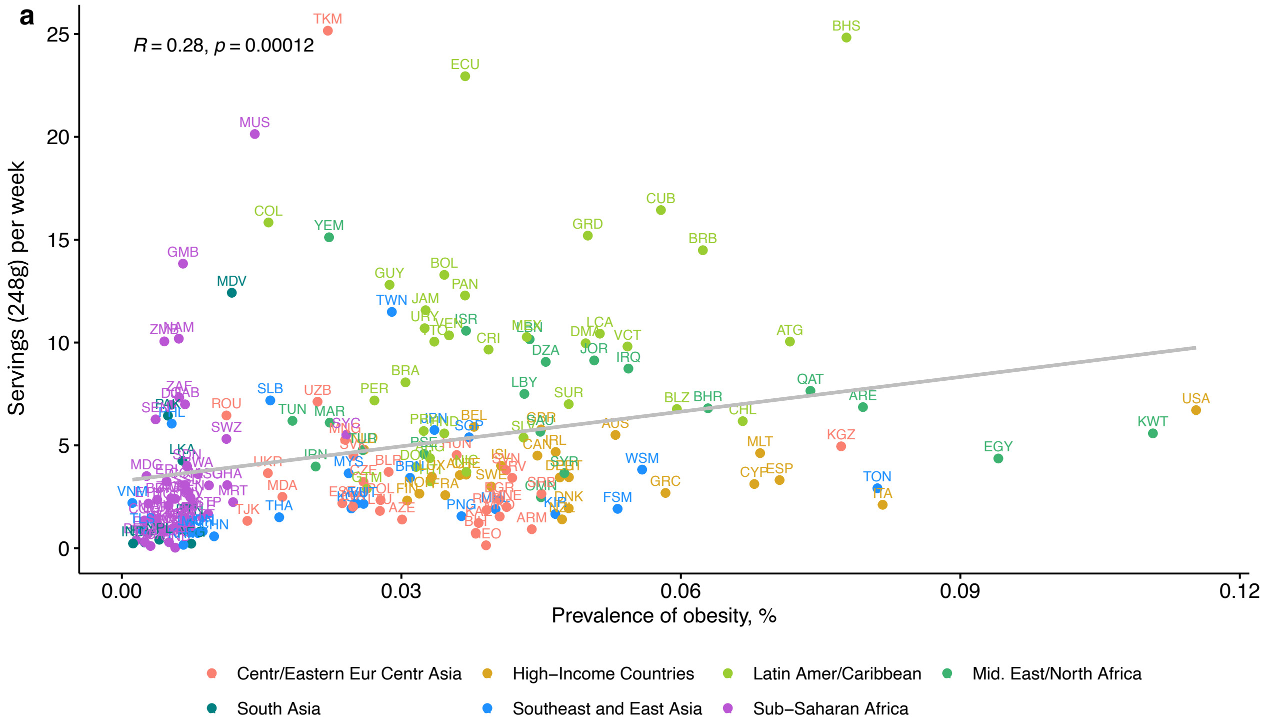

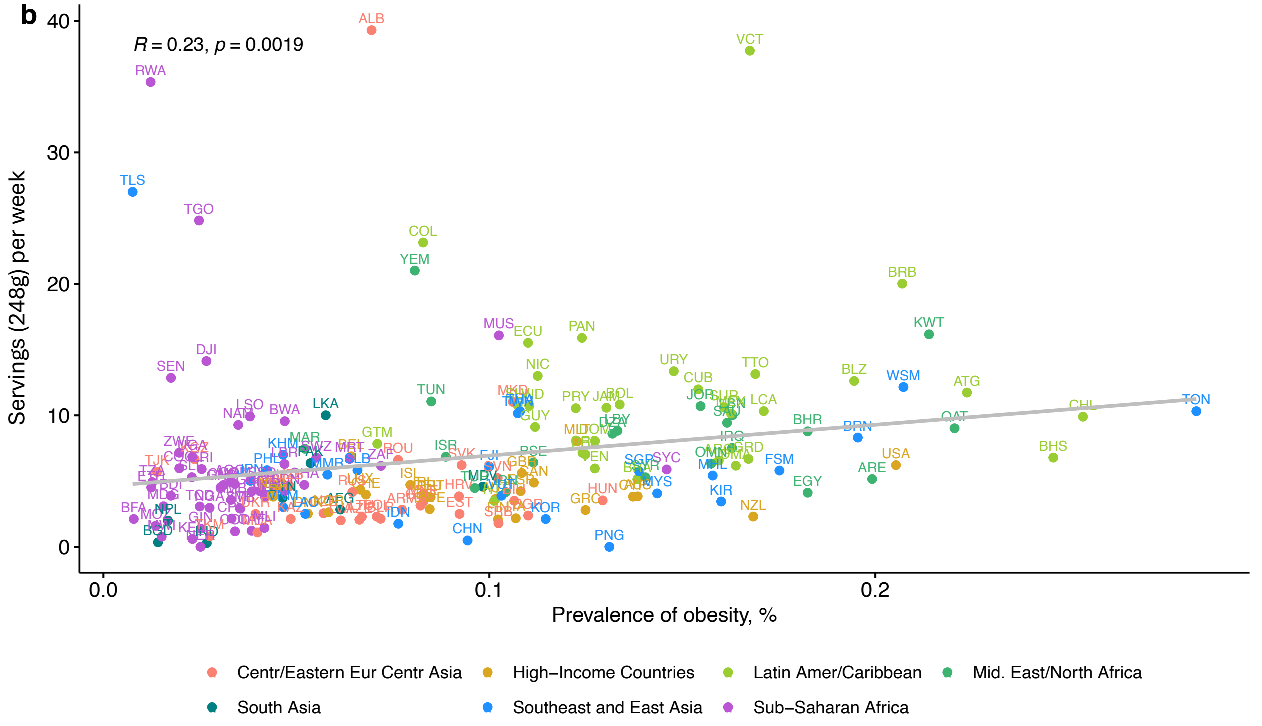

Supplementary Figure 13. National correlation of sugar-sweetened beverage intake (248 g servings/week) in youth (3-19 years) and prevalence of obesity (%) in 1990 and 2018 for 185 countries. (a) National correlation of SSB intakes and prevalence of obesity by world region in 1990; **(b)** National correlation of SSB intakes and prevalence of obesity by world region in 2018. SSBs were defined as any beverage with added sugars and ≥209 kJ (50 kcal) per 237 g serving, including commercial or homemade beverages, soft drinks, energy drinks, fruit drinks, punch, lemonade, and aguas frescas. This definition excludes 100% fruit and vegetable juices, non-caloric artificially sweetened drinks, and sweetened milk. The standardized serving size used for this analysis is 248 grams (8 oz) serving. Prevalence of youth obesity (5-19 years old) was obtained from the NCD RisC collaboration (defined as >2 standard deviation BMI for age and sex). We used population proportions to get the weighted average prevalence of obesity for boys and girls combined.

Centr/East Eur & Centr Asia, Central/Eastern Europe and Central Asia; GDD, Global Dietary Database; Latin Amer/Caribbean, Latin America/Caribbean; SSBs, sugar-sweetened beverages; UIs, uncertainty intervals.

Supplementary Table 15. Absolute change in mean sugar-sweetened beverage intakes (248 g servings/week) from 1990-2005, 2005-2018, and 1990-2018 in youth (3-19 years) by sex, age, parental education, and area of residence and by world region across 185 countries.

		World	Central/ Eastern Europe and Central Asia†	High-Income Countries	Latin America and Caribbean	Middle East and North Africa	South Asia†	Southeast and East Asia	Sub-Saharan Africa
		mean (95% UI)	*mean (95% UI)*	*mean (95% UI)*	*mean (95% UI)*	*mean (95% UI)*	*mean (95% UI)*	*mean (95% UI)*	*mean (95% UI)*
Sex									
Girls	1990-2005	0.31 (0.23,0.43)	-0.04 (-0.56,0.33)	1.34 (1.23,1.46)	-1.16 (-1.60,-0.74)	0.69 (0.37,1.13)	0.10 (0.07,0.16)	0.27 (0.18,0.42)	0.75 (0.47,1.19)
	2005-2018	0.36 (0.24,0.49)	0.43 (0.20,0.78)	-1.44 (-1.56,-1.33)	0.83 (0.43,1.24)	0.07 (-0.22,0.37)	-0.02 (-0.07,0.06)	0.30 (0.18,0.48)	1.35 (0.93,1.92)
	1990-2018	0.67 (0.52,0.87)	0.41 (-0.07,0.80)	-0.10 (-0.12,-0.07)	-0.34 (-0.75,0.07)	0.76 (0.43,1.19)	0.09 (0.02,0.20)	0.56 (0.40,0.87)	2.12 (1.53,2.88)
Boys	1990-2005	0.34 (0.25,0.45)	0.01 (-0.50,0.40)	1.62 (1.48,1.75)	-1.24 (-1.71,-0.82)	0.68 (0.36,1.12)	0.10 (0.06,0.15)	0.28 (0.19,0.43)	0.79 (0.49,1.25)
	2005-2018	0.35 (0.24,0.48)	0.48 (0.23,0.82)	-1.72 (-1.87,-1.59)	0.88 (0.48,1.28)	0.09 (-0.20,0.38)	0.00 (-0.06,0.07)	0.30 (0.19,0.47)	1.40 (0.96,1.97)
	1990-2018	0.68 (0.53,0.87)	0.49 (0.01,0.90)	-0.11 (-0.14,-0.08)	-0.36 (-0.80,0.03)	0.77 (0.45,1.19)	0.10 (0.03,0.21)	0.58 (0.41,0.86)	2.21 (1.63,2.99)
Age									
3-4 years	1990-2005	0.14 (0.09,0.20)	-0.04 (-0.32,0.14)	0.54 (0.49,0.60)	-0.58 (-0.90,-0.31)	0.30 (0.14,0.52)	0.12 (0.08,0.19)	0.12 (0.08,0.19)	0.27 (0.16,0.45)
	2005-2018	0.15 (0.09,0.24)	0.18 (0.06,0.37)	-0.58 (-0.64,-0.53)	0.41 (0.16,0.66)	0.03 (-0.14,0.21)	-0.02 (-0.10,0.10)	0.13 (0.07,0.21)	0.50 (0.29,0.79)
	1990-2018	0.30 (0.22,0.41)	0.14 (-0.11,0.36)	-0.04 (-0.05,-0.03)	-0.17 (-0.49,0.10)	0.33 (0.18,0.56)	0.10 (0.01,0.25)	0.24 (0.16,0.37)	0.77 (0.52,1.13)
5-9 years	1990-2005	0.28 (0.19,0.40)	-0.02 (-0.60,0.38)	1.18 (1.09,1.27)	-1.01 (-1.54,-0.50)	0.66 (0.31,1.14)	0.11 (0.07,0.18)	0.24 (0.16,0.38)	0.60 (0.35,1.01)
	2005-2018	0.31 (0.19,0.46)	0.37 (0.08,0.77)	-1.26 (-1.36,-1.17)	0.71 (0.25,1.15)	0.07 (-0.29,0.43)	-0.01 (-0.08,0.08)	0.26 (0.15,0.42)	1.14 (0.69,1.73)
	1990-2018	0.60 (0.45,0.78)	0.35 (-0.20,0.81)	-0.08 (-0.10,-0.06)	-0.30 (-0.82,0.14)	0.72 (0.38,1.20)	0.10 (0.02,0.23)	0.50 (0.35,0.76)	1.75 (1.22,2.48)
10-14 years	1990-2005	0.40 (0.29,0.57)	0.06 (-0.72,0.61)	1.78 (1.64,1.92)	-1.38 (-2.01,-0.76)	0.88 (0.42,1.48)	0.10 (0.06,0.16)	0.34 (0.22,0.52)	0.97 (0.57,1.63)
	2005-2018	0.42 (0.26,0.61)	0.54 (0.15,1.07)	-1.90 (-2.04,-1.76)	0.99 (0.42,1.53)	0.10 (-0.37,0.59)	-0.01 (-0.06,0.08)	0.37 (0.20,0.60)	1.72 (1.07,2.58)
	1990-2018	0.83 (0.63,1.07)	0.60 (-0.13,1.22)	-0.12 (-0.15,-0.09)	-0.40 (-1.01,0.16)	0.97 (0.50,1.60)	0.09 (0.03,0.21)	0.71 (0.50,1.07)	2.71 (1.92,3.77)
15-19 years	1990-2005	0.41 (0.28,0.58)	-0.04 (-0.87,0.52)	2.02 (1.86,2.18)	-1.56 (-2.20,-0.93)	0.81 (0.38,1.39)	0.09 (0.06,0.14)	0.33 (0.22,0.52)	1.18 (0.69,1.99)
	2005-2018	0.45 (0.28,0.65)	0.69 (0.28,1.25)	-2.17 (-2.33,-2.01)	1.12 (0.55,1.69)	0.12 (-0.36,0.58)	-0.01 (-0.05,0.07)	0.38 (0.21,0.61)	2.08 (1.32,3.06)
	1990-2018	0.87 (0.67,1.12)	0.65 (-0.16,1.31)	-0.14 (-0.18,-0.11)	-0.44 (-1.08,0.14)	0.93 (0.49,1.51)	0.08 (0.02,0.18)	0.71 (0.49,1.07)	3.28 (2.38,4.48)
Parental education									
0-6 years	1990-2005	0.36 (0.25,0.51)	0.23 (-0.23,0.62)	1.09 (0.98,1.21)	-1.00 (-1.53,-0.48)	0.97 (0.58,1.54)	0.06 (0.03,0.11)	0.49 (0.36,0.70)	0.70 (0.45,1.11)
	2005-2018	0.50 (0.36,0.67)	0.40 (0.14,0.74)	-0.71 (-0.78,-0.64)	0.65 (0.19,1.09)	0.07 (-0.20,0.37)	0.02 (-0.04,0.10)	0.44 (0.26,0.69)	1.18 (0.79,1.70)
	1990-2018	0.86 (0.67,1.09)	0.63 (0.18,1.04)	0.38 (0.32,0.46)	-0.35 (-0.84,0.07)	1.05 (0.64,1.60)	0.07 (0.01,0.20)	0.93 (0.68,1.34)	1.90 (1.36,2.62)
>6-12 years	1990-2005	0.20 (0.11,0.32)	-0.15 (-0.68,0.24)	1.07 (0.97,1.18)	-1.47 (-1.93,-1.04)	0.31 (-0.03,0.80)	0.08 (0.05,0.13)	0.19 (0.13,0.29)	0.99 (0.57,1.65)
	2005-2018	0.42 (0.31,0.56)	0.63 (0.35,1.01)	-0.97 (-1.06,-0.88)	1.05 (0.65,1.48)	0.18 (-0.25,0.63)	-0.01 (-0.05,0.02)	0.22 (0.14,0.34)	1.86 (1.26,2.70)
	1990-2018	0.63 (0.47,0.83)	0.49 (-0.02,0.92)	0.11 (0.07,0.14)	-0.41 (-0.85,0.01)	0.49 (0.16,0.96)	0.07 (0.02,0.13)	0.41 (0.29,0.62)	2.88 (2.05,4.03)
>12 years	1990-2005	0.46 (0.35,0.56)	0.00 (-0.55,0.41)	1.81 (1.67,1.96)	-1.14 (-1.75,-0.59)	0.35 (0.07,0.70)	0.58 (0.37,0.83)	0.01 (-0.04,0.09)	0.61 (0.18,1.33)
	2005-2018	-0.19 (-0.28,-0.08)	0.37 (0.11,0.74)	-2.14 (-2.30,-1.98)	0.92 (0.44,1.44)	-0.03 (-0.39,0.33)	-0.20 (-0.37,-0.03)	0.18 (0.11,0.28)	1.67 (1.06,2.49)
	1990-2018	0.27 (0.15,0.40)	0.39 (-0.14,0.81)	-0.33 (-0.35,-0.30)	-0.22 (-0.77,0.32)	0.32 (0.05,0.69)	0.38 (0.14,0.69)	0.19 (0.09,0.36)	2.31 (1.53,3.43)
Area of residence									
Rural	1990-2005	0.34 (0.27,0.45)	-0.50 (-1.17,-0.01)	1.48 (1.36,1.59)	-0.92 (-1.34,-0.55)	0.83 (0.44,1.40)	0.07 (0.05,0.11)	0.42 (0.31,0.60)	0.68 (0.45,1.03)
	2005-2018	0.47 (0.35,0.61)	0.75 (0.39,1.25)	-1.54 (-1.65,-1.43)	1.16 (0.80,1.57)	0.15 (0.00,0.31)	0.01 (-0.02,0.05)	0.35 (0.21,0.55)	1.33 (0.95,1.84)
	1990-2018	0.82 (0.65,1.02)	0.26 (-0.37,0.77)	-0.06 (-0.09,-0.04)	0.24 (-0.19,0.67)	0.98 (0.59,1.55)	0.08 (0.04,0.15)	0.77 (0.58,1.11)	2.02 (1.48,2.73)
Urban	1990-2005	0.31 (0.21,0.44)	0.28 (-0.13,0.60)	1.49 (1.37,1.61)	-1.28 (-1.67,-0.91)	0.60 (0.32,0.98)	0.16 (0.10,0.27)	0.15 (0.08,0.26)	0.92 (0.50,1.62)
	2005-2018	0.23 (0.12,0.37)	0.28 (0.10,0.51)	-1.60 (-1.73,-1.47)	0.77 (0.42,1.12)	0.04 (-0.26,0.36)	-0.04 (-0.14,0.10)	0.25 (0.17,0.39)	1.45 (0.90,2.19)
	1990-2018	0.54 (0.39,0.73)	0.57 (0.17,0.89)	-0.11 (-0.14,-0.09)	-0.51 (-0.89,-0.17)	0.64 (0.35,1.02)	0.12 (-0.01,0.33)	0.40 (0.27,0.64)	2.39 (1.66,3.40)

*Data are mean absolute change in intakes (95% uncertainty interval) in 248 g servings per week. The standardized serving size used for this analysis is 248 g (8 oz) serving. † In prior GDD reports, the region Central/ Eastern Europe and Central Asia was referred as Former Soviet Union, and Southeast and East Asia was referred as Asia.

Supplementary Results

SSB intake by parental education and residence in 2018

Differences in SSB intakes by parental education (i.e., intakes in youth of high educated parents minus intakes in youth of low educated parents) varied substantially by world region. Intakes were higher among youth of more vs. less educated parents in South Asia (+3·41 weekly servings higher; +316·3% relative difference), Sub-Saharan Africa (+2·89; +85·1%), and Latin America/Caribbean (+1·50; +18·1%), but lower among youth of more vs. less educated parents in Middle East/North Africa (-2·30; -28·7%); with smaller differences by parental education in other regions (Supplementary Table 7). Absolute differences in intake by high vs. low parental education increased as age of youth increased in all world regions, except for South Asia, where the differences decreased as age increased (Supplementary Figure 3a). Further accounting for urbanicity, differences by parental education were largest among urban youth in South Asia, were youth of higher educated parents had higher intakes compared to children of lower educated parents (+7·01, +306·7%) (Supplementary Figure 3b). Among the 25 most populous countries, the largest differences in SSB intake in youth of high vs. low educated parents were in Pakistan and Uganda where youth of more educated parents generally had 4·5+ servings/week and 6·5+ higher intake than the less educated, respectively (Supplementary Table 9).

Trends over time by age, sex, parental education, and urbanicity by world region

Trends in intakes were similar among boys and girls, with both showing an overall increase globally and in most regions from 1990 to 2018, with the largest increase in Sub-Saharan Africa (girls: +2·12 [1·53, 2·88], +106%; boys: +2·21 [1·63, 2·99] , +105%;), and slight decreases in Latin America/Caribbean and High-Income (Supplementary Table 15). Similar regional patterns were observed by age, parental education, and area of residence. By age, differences were largest among the oldest ages, with the largest increase from 1990 to 2018 in Sub-Saharan Africa among youth age 10-14 years (+2·71 [1·92, 3·77]; +106%) and 15-19 years (+3·28 [2·38, 4·48]; +99·3%). By parental education, the largest increases from 1990 to 2018 were observed in youth of mid-educated parents (+2·88 [2·05, 4·03]; +97·2%) and highly educated parents (+2·31[21·53, 3·43]; +57.8%) in Sub-Saharan Africa. By area of residence, the largest increases were in rural (+2·02 [1·48, 2·73]; +128%) and urban (+2·39 [1·66, 3·40]; +85·2%) areas in Sub-Saharan Africa in the period from 1990 to 2018.

Trends over time by age, sex, parental education, and urbanicity within the 25 most populous countries

Within the 25 most populous countries, trends were similar between girls and boys (Supplementary Table 16). By age, from 1990 to 2018 the largest increases were in Uganda among youth age 15-19 years (+10.54 [5.86, 19.05]; +5593.4%) and 10-14 years (+8.35 [4.61, 15.38]; +5611.6%); and the largest decreases in Brazil in youth age 15-19 years (-4.10 [-4.71, -3.57]; -39.0%) and 10-14 years (-3.80 [-4.39, -3.29]; -39.0%) (Supplementary Table 17). By parental education, from 1990 to 2018 the largest increases were in Uganda among youth of high educated (+10.24 [5.90, 17.15]; +5515.2%) and medium educated (+9.20 [5.37, 15.54]; +5526.6%) parents, and the largest decreases in Brazil among youth of high (-3.43 [-4.08, -2.86]; -39.0%), medium (-3.40 [-3.97, -2.91]; -39.0%), and low educated (-3.16 [-3.72, -2.71]; -39.0%) parents (Supplementary Table 18). Finally, by rural and urban area, the largest increases in SSB intake between 1990 and 2018 were among urban youth in Uganda (+9.31 [5.70, 15.13]; +5508.5%) and Afghanistan (+6.24 [2.92, 13.75]; +1098.1 %), and the largest decreases in urban (-3.35 [-3.86, -2.91]; -39.0%) and rural (-2.94 [-3.48, -2.49]; -39.0%) youth in Brazil (Supplementary Table 19).

Supplementary Table 16. Absolute change in mean sugar-sweetened beverage intakes (248 g servings/week) in youth (3-19 years) from 1990-2005, 2005-2018, and 1990-2018 by sex in the 25 most populous countries.

	Girls			Boys		
	1990-2005	2005-2018	1990-2018	1990-2005	2005-2018	1990-2018
Country	*mean (95% UI)*	*mean (95% UI)*	*mean (95% UI)*	*mean (95% UI)*	*mean (95% UI)*	*mean (95% UI)*
India	0.00 (-0.01,0.00)	0.02 (0.01,0.04)	0.02 (0.01,0.04)	0.00 (-0.01,0.00)	0.03 (0.02,0.06)	0.03 (0.02,0.06)
China	-0.10 (-0.13,-0.07)	-0.01 (-0.01,-0.01)	-0.11 (-0.14,-0.08)	-0.10 (-0.13,-0.08)	-0.01 (-0.01,-0.01)	-0.11 (-0.15,-0.08)
Nigeria	2.43 (1.45,4.12)	0.09 (-1.42,1.69)	2.53 (1.47,4.40)	2.57 (1.53,4.39)	0.11 (-1.42,1.75)	2.68 (1.62,4.60)
Pakistan	-0.05 (-0.08,-0.03)	-0.56 (-0.87,-0.35)	-0.61 (-0.95,-0.39)	-0.05 (-0.08,-0.03)	-0.56 (-0.86,-0.36)	-0.61 (-0.94,-0.40)
Indonesia	0.40 (0.29,0.55)	0.48 (0.34,0.67)	0.87 (0.63,1.22)	0.44 (0.31,0.60)	0.53 (0.38,0.73)	0.97 (0.69,1.33)
United States	2.67 (2.46,2.88)	-3.22 (-3.48,-2.97)	-0.55 (-0.59,-0.51)	3.21 (2.96,3.47)	-3.87 (-4.18,-3.58)	-0.66 (-0.71,-0.62)
Brazil	-3.26 (-3.85,-2.78)	0.13 (0.11,0.15)	-3.14 (-3.70,-2.67)	-3.56 (-4.24,-3.03)	0.14 (0.12,0.16)	-3.43 (-4.07,-2.91)
Bangladesh	0.04 (0.02,0.06)	-0.05 (-0.08,-0.03)	-0.01 (-0.02,-0.01)	0.04 (0.02,0.07)	-0.05 (-0.09,-0.03)	-0.01 (-0.02,-0.01)
Ethiopia	1.04 (0.75,1.43)	1.03 (0.73,1.45)	2.07 (1.49,2.88)	1.09 (0.78,1.50)	1.08 (0.76,1.52)	2.17 (1.54,3.01)
DR Congo	0.16 (0.06,0.47)	0.06 (0.02,0.16)	0.22 (0.07,0.64)	0.18 (0.06,0.54)	0.06 (0.02,0.19)	0.25 (0.08,0.73)
Mexico	-0.40 (-0.56,-0.25)	0.04 (0.03,0.06)	-0.36 (-0.50,-0.23)	-0.46 (-0.64,-0.29)	0.05 (0.03,0.07)	-0.41 (-0.57,-0.26)
Philippines	-0.43 (-0.53,-0.34)	0.01 (0.01,0.02)	-0.41 (-0.52,-0.33)	-0.37 (-0.48,-0.28)	0.01 (0.01,0.02)	-0.36 (-0.46,-0.27)
Egypt	-0.30 (-0.44,-0.19)	0.04 (0.02,0.05)	-0.27 (-0.38,-0.16)	-0.25 (-0.37,-0.15)	0.03 (0.02,0.05)	-0.22 (-0.32,-0.13)
Russia	2.16 (1.73,2.64)	0.06 (0.05,0.07)	2.21 (1.78,2.71)	2.42 (1.97,2.99)	0.06 (0.05,0.08)	2.48 (2.02,3.07)
Tanzania	2.43 (1.42,4.20)	1.95 (0.12,4.51)	4.44 (2.65,7.37)	2.57 (1.50,4.38)	2.02 (0.18,4.62)	4.62 (2.80,7.75)
Vietnam	0.93 (0.59,1.35)	-0.13 (-0.20,-0.08)	0.80 (0.51,1.15)	0.93 (0.59,1.35)	-0.13 (-0.20,-0.08)	0.80 (0.51,1.15)
Turkey	-1.38 (-1.84,-1.04)	1.12 (0.84,1.49)	-0.26 (-0.34,-0.19)	-1.40 (-1.87,-1.06)	1.14 (0.86,1.52)	-0.26 (-0.35,-0.20)
Kenya	-0.28 (-0.46,-0.17)	-0.16 (-0.26,-0.09)	-0.44 (-0.72,-0.26)	-0.34 (-0.58,-0.20)	-0.19 (-0.33,-0.11)	-0.53 (-0.91,-0.31)
Iran	0.10 (0.06,0.14)	0.14 (0.09,0.20)	0.24 (0.15,0.34)	0.11 (0.07,0.15)	0.15 (0.09,0.21)	0.25 (0.16,0.36)
Uganda	2.31 (1.37,4.03)	4.15 (1.69,8.11)	6.50 (3.91,11.06)	2.40 (1.44,4.14)	4.31 (1.83,8.37)	6.79 (4.14,11.12)
Japan	-0.37 (-0.53,-0.23)	-0.22 (-0.32,-0.14)	-0.59 (-0.84,-0.37)	-0.42 (-0.61,-0.26)	-0.26 (-0.36,-0.16)	-0.68 (-0.97,-0.42)
South Africa	-3.12 (-4.21,-2.32)	1.39 (1.04,1.88)	-1.72 (-2.34,-1.28)	-3.37 (-4.52,-2.47)	1.51 (1.10,2.02)	-1.87 (-2.51,-1.37)
Sudan	-0.01 (-0.02,0.00)	0.07 (0.03,0.12)	0.06 (0.03,0.10)	-0.01 (-0.02,0.00)	0.07 (0.03,0.13)	0.06 (0.03,0.11)
Afghanistan	0.99 (0.46,2.27)	1.42 (0.40,3.68)	2.45 (1.20,5.33)	1.07 (0.50,2.41)	1.53 (0.48,3.85)	2.64 (1.30,5.58)
Myanmar	2.70 (1.50,4.86)	1.77 (-0.50,4.98)	4.50 (2.63,7.97)	2.89 (1.68,5.29)	1.85 (-0.59,5.12)	4.79 (2.91,8.27)

*Data are mean absolute difference in intakes (95% uncertainty interval) in 248 g servings per week. The standardized serving size used for this analysis is 248 g (8 oz) serving. Countries are ordered top to bottom from most to least populous based on 2018 youth (3-19 years) population data.

Supplementary Table 17. Absolute change in mean sugar-sweetened beverage intakes (248 g servings/week) in youth (3-19 years) from 1990-2005, 2005-2018, and 1990-2018 by age in the 25 most populous countries.

	3-4 years			5-9 years			10-14 years			15-19 years		
	1990-2005	2005-2018	1990-2018	1990-2005	2005-2018	1990-2018	1990-2005	2005-2018	1990-2018	1990-2005	2005-2018	1990-2018
Country	*mean (95% UI)*	*mean (95% UI)*	*mean (95% UI)*	*mean (95% UI)*	*mean (95% UI)*	*mean (95% UI)*	*mean (95% UI)*	*mean (95% UI)*	*mean (95% UI)*	*mean (95% UI)*	*mean (95% UI)*	*mean (95% UI)*
India	0.00 (-0.01,0.00)	0.03 (0.02,0.06)	0.03 (0.02,0.05)	0.00 (-0.01,0.00)	0.03 (0.02,0.06)	0.03 (0.02,0.05)	0.00 (-0.01,0.00)	0.03 (0.02,0.05)	0.03 (0.01,0.05)	0.00 (-0.01,0.00)	0.03 (0.01,0.05)	0.02 (0.01,0.04)
China	-0.04 (-0.06,-0.03)	0.00 (-0.01,0.00)	-0.05 (-0.07,-0.03)	-0.09 (-0.12,-0.07)	-0.01 (-0.01,-0.01)	-0.10 (-0.13,-0.08)	-0.12 (-0.16,-0.10)	-0.01 (-0.01,-0.01)	-0.13 (-0.17,-0.10)	-0.12 (-0.15,-0.09)	-0.01 (-0.01,-0.01)	-0.13 (-0.17,-0.10)
Nigeria	0.83 (0.45,1.58)	0.02 (-0.65,0.74)	0.85 (0.46,1.65)	1.94 (1.03,3.55)	0.09 (-1.45,1.70)	2.01 (1.09,3.81)	3.17 (1.69,5.93)	0.09 (-2.47,2.83)	3.28 (1.79,6.20)	3.97 (2.17,7.39)	0.19 (-3.10,3.58)	4.18 (2.24,7.73)
Pakistan	-0.06 (-0.09,-0.04)	-0.63 (-0.97,-0.40)	-0.69 (-1.06,-0.44)	-0.05 (-0.08,-0.03)	-0.58 (-0.89,-0.39)	-0.63 (-0.97,-0.42)	-0.05 (-0.07,-0.03)	-0.54 (-0.82,-0.36)	-0.58 (-0.89,-0.39)	-0.05 (-0.07,-0.03)	-0.50 (-0.76,-0.33)	-0.54 (-0.83,-0.36)
Indonesia	0.19 (0.13,0.27)	0.22 (0.15,0.33)	0.41 (0.28,0.60)	0.38 (0.28,0.52)	0.46 (0.33,0.63)	0.85 (0.61,1.15)	0.51 (0.38,0.69)	0.62 (0.45,0.84)	1.13 (0.84,1.53)	0.50 (0.38,0.67)	0.61 (0.45,0.82)	1.11 (0.82,1.50)
United States	1.10 (1.00,1.19)	-1.32 (-1.44,-1.21)	-0.23 (-0.25,-0.21)	2.37 (2.19,2.55)	-2.85 (-3.07,-2.65)	-0.49 (-0.52,-0.46)	3.51 (3.25,3.78)	-4.23 (-4.55,-3.92)	-0.72 (-0.77,-0.67)	3.97 (3.68,4.26)	-4.79 (-5.14,-4.45)	-0.82 (-0.88,-0.76)
Brazil	-1.65 (-1.93,-1.41)	0.06 (0.05,0.07)	-1.58 (-1.86,-1.35)	-2.96 (-3.44,-2.56)	0.11 (0.10,0.13)	-2.85 (-3.31,-2.46)	-3.95 (-4.57,-3.43)	0.15 (0.13,0.18)	-3.80 (-4.39,-3.29)	-4.26 (-4.90,-3.71)	0.16 (0.14,0.19)	-4.10 (-4.71,-3.57)
Bangladesh	0.05 (0.03,0.07)	-0.06 (-0.09,-0.04)	-0.01 (-0.02,-0.01)	0.04 (0.02,0.07)	-0.06 (-0.09,-0.03)	-0.01 (-0.02,-0.01)	0.04 (0.02,0.06)	-0.05 (-0.08,-0.03)	-0.01 (-0.02,-0.01)	0.04 (0.02,0.06)	-0.05 (-0.08,-0.03)	-0.01 (-0.02,-0.01)
Ethiopia	0.34 (0.26,0.45)	0.34 (0.25,0.46)	0.69 (0.51,0.91)	0.81 (0.59,1.10)	0.80 (0.57,1.11)	1.61 (1.16,2.21)	1.32 (0.95,1.81)	1.31 (0.92,1.83)	2.62 (1.88,3.64)	1.66 (1.22,2.24)	1.65 (1.18,2.25)	3.31 (2.40,4.49)
DR Congo	0.06 (0.02,0.17)	0.02 (0.01,0.06)	0.08 (0.03,0.23)	0.14 (0.05,0.40)	0.05 (0.02,0.14)	0.19 (0.06,0.54)	0.23 (0.08,0.66)	0.08 (0.03,0.23)	0.30 (0.10,0.88)	0.29 (0.10,0.83)	0.10 (0.03,0.28)	0.38 (0.13,1.12)
Mexico	-0.21 (-0.29,-0.13)	0.02 (0.01,0.03)	-0.19 (-0.26,-0.12)	-0.38 (-0.53,-0.24)	0.04 (0.02,0.05)	-0.34 (-0.47,-0.21)	-0.51 (-0.70,-0.32)	0.05 (0.03,0.07)	-0.45 (-0.63,-0.29)	-0.54 (-0.75,-0.35)	0.06 (0.04,0.08)	-0.49 (-0.67,-0.31)
Philippines	-0.18 (-0.24,-0.13)	0.01 (0.00,0.01)	-0.17 (-0.24,-0.12)	-0.37 (-0.46,-0.28)	0.01 (0.01,0.01)	-0.35 (-0.45,-0.27)	-0.49 (-0.61,-0.39)	0.02 (0.01,0.02)	-0.47 (-0.59,-0.38)	-0.48 (-0.59,-0.38)	0.02 (0.01,0.02)	-0.46 (-0.57,-0.37)
Egypt	-0.13 (-0.18,-0.08)	0.02 (0.01,0.02)	-0.11 (-0.16,-0.07)	-0.26 (-0.38,-0.16)	0.03 (0.02,0.05)	-0.23 (-0.33,-0.14)	-0.36 (-0.51,-0.21)	0.04 (0.03,0.06)	-0.31 (-0.45,-0.19)	-0.35 (-0.50,-0.21)	0.04 (0.03,0.06)	-0.31 (-0.44,-0.19)
Russia	0.79 (0.58,1.10)	0.02 (0.02,0.03)	0.82 (0.60,1.12)	2.01 (1.65,2.44)	0.05 (0.04,0.06)	2.06 (1.69,2.50)	3.02 (2.53,3.60)	0.08 (0.07,0.10)	3.10 (2.59,3.70)	3.12 (2.61,3.74)	0.08 (0.07,0.10)	3.21 (2.68,3.83)
Tanzania	0.82 (0.42,1.55)	0.66 (-0.13,1.75)	1.49 (0.80,2.69)	1.94 (1.02,3.60)	1.54 (-0.32,4.23)	3.53 (1.95,6.45)	3.16 (1.68,5.74)	2.53 (-0.50,6.56)	5.74 (3.14,10.11)	3.96 (2.13,7.43)	3.15 (-0.67,8.22)	7.16 (3.99,12.94)
Vietnam	0.42 (0.26,0.64)	-0.06 (-0.10,-0.04)	0.36 (0.22,0.54)	0.87 (0.56,1.24)	-0.13 (-0.18,-0.08)	0.75 (0.48,1.06)	1.17 (0.75,1.64)	-0.17 (-0.24,-0.10)	1.00 (0.64,1.39)	1.14 (0.73,1.61)	-0.16 (-0.24,-0.10)	0.98 (0.63,1.37)
Turkey	-0.61 (-0.80,-0.46)	0.49 (0.38,0.65)	-0.11 (-0.15,-0.09)	-1.26 (-1.65,-0.98)	1.03 (0.80,1.34)	-0.24 (-0.31,-0.18)	-1.71 (-2.21,-1.33)	1.39 (1.08,1.80)	-0.32 (-0.41,-0.25)	-1.68 (-2.19,-1.31)	1.37 (1.06,1.78)	-0.31 (-0.41,-0.24)
Kenya	-0.10 (-0.16,-0.06)	-0.06 (-0.09,-0.03)	-0.16 (-0.25,-0.09)	-0.23 (-0.38,-0.14)	-0.13 (-0.21,-0.08)	-0.36 (-0.60,-0.22)	-0.38 (-0.64,-0.23)	-0.21 (-0.35,-0.13)	-0.59 (-0.99,-0.35)	-0.48 (-0.79,-0.29)	-0.27 (-0.44,-0.16)	-0.75 (-1.24,-0.45)
Iran	0.05 (0.03,0.07)	0.06 (0.04,0.09)	0.11 (0.07,0.16)	0.10 (0.06,0.14)	0.13 (0.08,0.19)	0.23 (0.14,0.32)	0.13 (0.08,0.18)	0.18 (0.11,0.25)	0.31 (0.20,0.44)	0.13 (0.08,0.18)	0.18 (0.11,0.25)	0.31 (0.20,0.43)
Uganda	0.78 (0.43,1.45)	1.37 (0.45,3.04)	2.17 (1.21,3.97)	1.83 (1.00,3.37)	3.23 (0.93,7.31)	5.10 (2.83,9.33)	2.96 (1.63,5.75)	5.30 (1.45,11.92)	8.35 (4.61,15.38)	3.74 (1.97,6.86)	6.64 (2.08,14.36)	10.54 (5.86,19.05)
Japan	-0.17 (-0.26,-0.10)	-0.11 (-0.16,-0.06)	-0.28 (-0.42,-0.17)	-0.36 (-0.51,-0.22)	-0.22 (-0.31,-0.14)	-0.58 (-0.82,-0.36)	-0.48 (-0.68,-0.30)	-0.29 (-0.41,-0.19)	-0.77 (-1.09,-0.48)	-0.47 (-0.67,-0.29)	-0.29 (-0.40,-0.18)	-0.75 (-1.06,-0.47)
South Africa	-1.04 (-1.39,-0.78)	0.46 (0.35,0.62)	-0.57 (-0.77,-0.43)	-2.44 (-3.23,-1.85)	1.09 (0.83,1.44)	-1.35 (-1.79,-1.03)	-3.98 (-5.30,-2.99)	1.78 (1.34,2.36)	-2.20 (-2.93,-1.66)	-5.00 (-6.60,-3.82)	2.23 (1.71,2.94)	-2.77 (-3.66,-2.11)
Sudan	0.00 (-0.01,0.00)	0.02 (0.01,0.04)	0.02 (0.01,0.03)	-0.01 (-0.01,0.00)	0.05 (0.02,0.10)	0.05 (0.02,0.08)	-0.01 (-0.02,0.00)	0.08 (0.03,0.16)	0.07 (0.04,0.13)	-0.01 (-0.03,0.00)	0.11 (0.04,0.20)	0.09 (0.04,0.17)
Afghanistan	1.13 (0.47,2.86)	1.65 (0.14,5.11)	2.84 (1.27,6.88)	1.06 (0.43,2.73)	1.47 (0.13,4.34)	2.60 (1.18,6.11)	0.96 (0.40,2.47)	1.39 (0.15,4.22)	2.40 (1.07,5.76)	0.90 (0.37,2.22)	1.30 (0.16,3.85)	2.24 (1.01,5.23)
Myanmar	1.22 (0.58,2.55)	0.77 (-0.50,2.59)	2.01 (1.05,4.01)	2.47 (1.19,5.09)	1.60 (-1.17,5.29)	4.09 (2.21,8.12)	3.33 (1.62,6.76)	2.18 (-1.55,6.72)	5.52 (2.96,10.51)	3.25 (1.60,6.39)	2.13 (-1.29,7.19)	5.45 (2.91,10.81)

*Data are mean absolute difference in intakes (95% uncertainty interval) in 248 g servings per week. The standardized serving size used for this analysis is 248 g (8 oz) serving. Countries are ordered top to bottom from most to least populous based on 2018 youth (3-19 years) population data.

Supplementary Table 18. Absolute change in mean sugar-sweetened beverage intakes (248 g servings/week) in youth (3-19 years) from 1990-2005, 2005-2018, and 1990-2018 by parental education level in the 25 most populous countries.

	≤6 years of parental education			>6-12 years of parental education			>12 years of parental education		
	1990-2005	2005-2018	1990-2018	1990-2005	2005-2018	1990-2018	1990-2005	2005-2018	1990-2018
Country	*mean (95% UI)*	*mean (95% UI)*	*mean (95% UI)*	*mean (95% UI)*	*mean (95% UI)*	*mean (95% UI)*	*mean (95% UI)*	*mean (95% UI)*	*mean (95% UI)*
India	0.00 (0.00,0.00)	0.02 (0.01,0.04)	0.02 (0.01,0.04)	0.00 (-0.01,0.00)	0.04 (0.02,0.07)	0.03 (0.02,0.06)	-0.01 (-0.01,0.00)	0.05 (0.03,0.10)	0.05 (0.02,0.09)
China	-0.09 (-0.11,-0.07)	-0.01 (-0.01,-0.01)	-0.10 (-0.12,-0.07)	-0.11 (-0.14,-0.08)	-0.01 (-0.01,-0.01)	-0.11 (-0.15,-0.09)	-0.11 (-0.14,-0.08)	-0.01 (-0.01,-0.01)	-0.11 (-0.15,-0.09)
Nigeria	2.09 (1.23,3.63)	0.11 (-1.26,1.46)	2.19 (1.27,3.81)	3.55 (2.01,6.15)	0.13 (-2.12,2.39)	3.67 (2.09,6.33)	3.92 (2.21,6.97)	0.13 (-2.37,2.73)	4.08 (2.32,7.34)
Pakistan	-0.04 (-0.06,-0.02)	-0.41 (-0.66,-0.26)	-0.45 (-0.72,-0.28)	-0.07 (-0.11,-0.04)	-0.76 (-1.20,-0.47)	-0.82 (-1.31,-0.51)	-0.09 (-0.15,-0.06)	-1.00 (-1.65,-0.61)	-1.10 (-1.79,-0.67)
Indonesia	0.39 (0.29,0.52)	0.47 (0.34,0.63)	0.85 (0.63,1.15)	0.46 (0.34,0.62)	0.56 (0.41,0.76)	1.02 (0.75,1.38)	0.46 (0.34,0.62)	0.55 (0.40,0.76)	1.02 (0.74,1.38)
United States	3.78 (3.46,4.15)	-4.56 (-5.01,-4.18)	-0.78 (-0.85,-0.72)	3.72 (3.42,4.06)	-4.49 (-4.89,-4.13)	-0.77 (-0.83,-0.71)	2.74 (2.53,2.94)	-3.30 (-3.55,-3.06)	-0.56 (-0.60,-0.53)
Brazil	-3.29 (-3.87,-2.81)	0.13 (0.11,0.15)	-3.16 (-3.72,-2.71)	-3.54 (-4.13,-3.03)	0.14 (0.12,0.16)	-3.40 (-3.97,-2.91)	-3.57 (-4.25,-2.98)	0.14 (0.12,0.16)	-3.43 (-4.08,-2.86)
Bangladesh	0.03 (0.02,0.05)	-0.04 (-0.06,-0.02)	-0.01 (-0.01,-0.01)	0.05 (0.03,0.08)	-0.07 (-0.11,-0.04)	-0.02 (-0.03,-0.01)	0.07 (0.04,0.12)	-0.09 (-0.16,-0.05)	-0.02 (-0.04,-0.01)
Ethiopia	0.88 (0.63,1.20)	0.87 (0.61,1.21)	1.74 (1.24,2.40)	1.47 (1.03,2.07)	1.46 (1.01,2.09)	2.93 (2.04,4.16)	1.64 (1.12,2.42)	1.63 (1.09,2.44)	3.28 (2.22,4.85)
DR Congo	0.15 (0.05,0.43)	0.05 (0.02,0.15)	0.19 (0.07,0.57)	0.24 (0.08,0.73)	0.08 (0.03,0.25)	0.33 (0.11,0.97)	0.28 (0.09,0.79)	0.09 (0.03,0.27)	0.37 (0.12,1.07)
Mexico	-0.41 (-0.57,-0.26)	0.04 (0.03,0.06)	-0.37 (-0.51,-0.23)	-0.44 (-0.62,-0.28)	0.05 (0.03,0.06)	-0.40 (-0.55,-0.25)	-0.45 (-0.63,-0.28)	0.05 (0.03,0.06)	-0.40 (-0.56,-0.25)
Philippines	-0.35 (-0.43,-0.27)	0.01 (0.01,0.01)	-0.34 (-0.42,-0.27)	-0.42 (-0.52,-0.33)	0.01 (0.01,0.02)	-0.40 (-0.50,-0.32)	-0.41 (-0.52,-0.32)	0.01 (0.01,0.02)	-0.40 (-0.50,-0.31)
Egypt	-0.28 (-0.40,-0.17)	0.04 (0.02,0.05)	-0.24 (-0.35,-0.15)	-0.30 (-0.43,-0.18)	0.04 (0.02,0.05)	-0.26 (-0.38,-0.16)	-0.25 (-0.36,-0.15)	0.03 (0.02,0.04)	-0.22 (-0.31,-0.13)
Russia	2.23 (1.68,2.97)	0.06 (0.04,0.08)	2.29 (1.73,3.05)	2.45 (1.96,3.07)	0.06 (0.05,0.08)	2.52 (2.01,3.15)	2.24 (1.86,2.70)	0.06 (0.05,0.07)	2.30 (1.91,2.77)
Tanzania	1.96 (1.15,3.39)	1.54 (0.09,3.72)	3.53 (2.08,6.06)	3.29 (1.89,5.77)	2.62 (0.17,6.19)	6.02 (3.48,10.00)	3.70 (2.05,6.50)	2.86 (0.19,6.77)	6.62 (3.81,11.41)
Vietnam	0.85 (0.54,1.20)	-0.12 (-0.18,-0.07)	0.72 (0.47,1.02)	1.02 (0.65,1.43)	-0.15 (-0.21,-0.09)	0.87 (0.56,1.22)	1.01 (0.64,1.44)	-0.15 (-0.22,-0.09)	0.86 (0.56,1.22)
Turkey	-1.37 (-1.81,-1.05)	1.11 (0.86,1.48)	-0.26 (-0.34,-0.20)	-1.48 (-1.92,-1.15)	1.21 (0.93,1.56)	-0.28 (-0.36,-0.21)	-1.21 (-1.59,-0.92)	0.99 (0.75,1.29)	-0.23 (-0.30,-0.17)
Kenya	-0.24 (-0.40,-0.14)	-0.14 (-0.22,-0.08)	-0.38 (-0.62,-0.22)	-0.41 (-0.70,-0.24)	-0.23 (-0.39,-0.13)	-0.64 (-1.08,-0.37)	-0.46 (-0.79,-0.26)	-0.25 (-0.44,-0.14)	-0.71 (-1.23,-0.40)
Iran	0.10 (0.07,0.15)	0.14 (0.09,0.20)	0.25 (0.16,0.35)	0.11 (0.07,0.16)	0.16 (0.10,0.22)	0.27 (0.17,0.38)	0.09 (0.06,0.13)	0.13 (0.08,0.18)	0.22 (0.14,0.31)
Uganda	1.96 (1.14,3.32)	3.50 (1.49,6.85)	5.51 (3.25,9.33)	3.29 (1.94,5.73)	5.81 (2.39,11.39)	9.20 (5.37,15.54)	3.64 (2.04,6.61)	6.49 (2.73,12.67)	10.24 (5.90,17.15)
Japan	-0.34 (-0.48,-0.21)	-0.20 (-0.29,-0.13)	-0.54 (-0.77,-0.34)	-0.40 (-0.58,-0.25)	-0.25 (-0.35,-0.15)	-0.65 (-0.93,-0.40)	-0.40 (-0.57,-0.25)	-0.24 (-0.34,-0.16)	-0.65 (-0.91,-0.41)
South Africa	-2.24 (-3.10,-1.61)	1.00 (0.72,1.38)	-1.24 (-1.72,-0.89)	-3.77 (-5.09,-2.81)	1.68 (1.26,2.27)	-2.09 (-2.82,-1.55)	-4.22 (-5.74,-3.10)	1.88 (1.38,2.56)	-2.34 (-3.18,-1.71)
Sudan	-0.01 (-0.02,0.00)	0.06 (0.02,0.10)	0.05 (0.02,0.09)	-0.01 (-0.03,0.00)	0.09 (0.04,0.18)	0.08 (0.04,0.15)	-0.01 (-0.03,0.00)	0.10 (0.04,0.21)	0.09 (0.04,0.17)
Afghanistan	0.90 (0.41,2.05)	1.29 (0.42,3.16)	2.24 (1.10,4.78)	1.64 (0.74,3.67)	2.45 (0.80,5.81)	4.15 (1.99,8.73)	2.20 (1.00,4.91)	3.18 (1.07,7.46)	5.49 (2.56,11.46)
Myanmar	2.63 (1.50,4.79)	1.73 (-0.49,4.93)	4.41 (2.61,7.86)	3.17 (1.78,5.63)	2.01 (-0.77,5.57)	5.19 (3.08,9.02)	3.14 (1.72,5.62)	2.10 (-0.58,5.56)	5.27 (3.03,9.16)

*Data are mean absolute difference in intakes (95% uncertainty interval) in 248 g servings per week. The standardized serving size used for this analysis is 248 g (8 oz) serving. Countries are ordered top to bottom from most to least populous based on 2018 youth (3-19 years) population data.

Supplementary Table 19. Absolute change in mean sugar-sweetened beverage intakes (248 g servings/week) in youth (3-19 years) from 1990-2005, 2005-2018, and 1990-2018 by area of residence in the 25 most populous countries.

	Rural			Urban		
	1990-2005	2005-2018	1990-2018	1990-2005	2005-2018	1990-2018
Country	*mean (95% UI)*	*mean (95% UI)*	*mean (95% UI)*	*mean (95% UI)*	*mean (95% UI)*	*mean (95% UI)*
India	0.00 (0.00,0.00)	0.01 (0.01,0.02)	0.01 (0.01,0.02)	-0.01 (-0.01,0.00)	0.06 (0.03,0.12)	0.06 (0.03,0.11)
China	-0.10 (-0.12,-0.08)	-0.01 (-0.01,-0.01)	-0.11 (-0.14,-0.08)	-0.10 (-0.13,-0.08)	-0.01 (-0.01,-0.01)	-0.11 (-0.14,-0.09)
Nigeria	1.94 (1.16,3.32)	0.08 (-1.08,1.38)	2.04 (1.21,3.50)	3.10 (1.88,5.37)	0.13 (-1.81,2.09)	3.21 (1.87,5.58)
Pakistan	-0.02 (-0.03,-0.01)	-0.24 (-0.37,-0.16)	-0.26 (-0.40,-0.17)	-0.10 (-0.16,-0.06)	-1.11 (-1.75,-0.70)	-1.21 (-1.91,-0.77)
Indonesia	0.41 (0.30,0.55)	0.49 (0.36,0.67)	0.90 (0.67,1.22)	0.43 (0.31,0.58)	0.51 (0.37,0.70)	0.94 (0.69,1.28)
United States	3.06 (2.86,3.26)	-3.69 (-3.93,-3.45)	-0.63 (-0.67,-0.59)	2.92 (2.69,3.16)	-3.52 (-3.81,-3.25)	-0.60 (-0.65,-0.56)
Brazil	-3.06 (-3.62,-2.60)	0.12 (0.10,0.14)	-2.94 (-3.48,-2.49)	-3.48 (-4.01,-3.02)	0.13 (0.12,0.15)	-3.35 (-3.86,-2.91)
Bangladesh	0.02 (0.01,0.03)	-0.02 (-0.04,-0.01)	-0.01 (-0.01,0.00)	0.08 (0.05,0.14)	-0.11 (-0.19,-0.06)	-0.03 (-0.04,-0.01)
Ethiopia	0.96 (0.69,1.31)	0.95 (0.67,1.32)	1.91 (1.37,2.63)	1.52 (1.09,2.10)	1.50 (1.06,2.12)	3.03 (2.16,4.22)
DR Congo	0.14 (0.05,0.40)	0.05 (0.02,0.14)	0.19 (0.06,0.54)	0.22 (0.07,0.65)	0.08 (0.03,0.22)	0.30 (0.10,0.87)
Mexico	-0.39 (-0.54,-0.24)	0.04 (0.03,0.06)	-0.35 (-0.48,-0.22)	-0.44 (-0.61,-0.28)	0.05 (0.03,0.06)	-0.40 (-0.55,-0.25)
Philippines	-0.39 (-0.49,-0.31)	0.01 (0.01,0.02)	-0.38 (-0.47,-0.30)	-0.41 (-0.51,-0.32)	0.01 (0.01,0.02)	-0.39 (-0.49,-0.31)
Egypt	-0.29 (-0.42,-0.18)	0.04 (0.02,0.05)	-0.26 (-0.37,-0.16)	-0.26 (-0.37,-0.15)	0.03 (0.02,0.05)	-0.22 (-0.32,-0.13)
Russia	2.45 (1.99,3.00)	0.06 (0.05,0.08)	2.51 (2.04,3.08)	2.24 (1.86,2.70)	0.06 (0.05,0.07)	2.30 (1.91,2.77)
Tanzania	2.11 (1.25,3.61)	1.69 (0.16,3.97)	3.82 (2.25,6.46)	3.37 (1.95,5.80)	2.62 (0.24,6.21)	6.10 (3.53,10.06)
Vietnam	0.92 (0.59,1.30)	-0.13 (-0.19,-0.08)	0.79 (0.51,1.11)	0.96 (0.62,1.37)	-0.14 (-0.20,-0.08)	0.83 (0.53,1.16)
Turkey	-1.54 (-2.02,-1.19)	1.25 (0.97,1.64)	-0.29 (-0.38,-0.22)	-1.34 (-1.74,-1.04)	1.09 (0.84,1.41)	-0.25 (-0.33,-0.19)
Kenya	-0.27 (-0.45,-0.16)	-0.15 (-0.25,-0.09)	-0.42 (-0.70,-0.25)	-0.43 (-0.72,-0.25)	-0.24 (-0.40,-0.14)	-0.67 (-1.12,-0.39)
Iran	0.11 (0.07,0.16)	0.16 (0.10,0.22)	0.27 (0.17,0.39)	0.10 (0.06,0.14)	0.14 (0.09,0.19)	0.24 (0.15,0.33)
Uganda	2.09 (1.25,3.56)	3.77 (1.61,7.20)	5.89 (3.57,9.90)	3.33 (2.00,5.53)	5.91 (2.75,10.97)	9.31 (5.70,15.13)
Japan	-0.38 (-0.55,-0.24)	-0.23 (-0.33,-0.15)	-0.61 (-0.88,-0.38)	-0.40 (-0.56,-0.25)	-0.24 (-0.34,-0.15)	-0.64 (-0.90,-0.40)
South Africa	-2.36 (-3.16,-1.76)	1.05 (0.79,1.41)	-1.30 (-1.75,-0.97)	-3.73 (-4.97,-2.78)	1.67 (1.25,2.22)	-2.07 (-2.76,-1.54)
Sudan	-0.01 (-0.02,0.00)	0.06 (0.02,0.10)	0.05 (0.02,0.09)	-0.01 (-0.03,0.00)	0.09 (0.03,0.17)	0.08 (0.04,0.14)
Afghanistan	0.54 (0.26,1.18)	0.77 (0.25,1.92)	1.34 (0.67,2.81)	2.50 (1.11,5.83)	3.61 (1.07,9.43)	6.24 (2.92,13.75)
Myanmar	2.77 (1.62,4.87)	1.81 (-0.31,4.88)	4.62 (2.86,7.99)	2.91 (1.67,5.10)	1.84 (-0.44,4.79)	4.80 (2.91,8.01)

*Data are mean absolute difference in intakes (95% uncertainty interval) in 248 g servings per week. The standardized serving size used for this analysis is 248 g (8 oz) serving. Countries are ordered top to bottom from most to least populous based on 2018 youth (3-19 years) population data.

Supplementary References

1. Vehtari A, Gelman A, Gabry J. Practical Bayesian model evaluation using leave-one-out cross-validation and WAIC. . *Stat Comput 27.* 2017:1413-1432.
2. Miller V, Singh GM, Onopa J, et al. The Global Dietary Database 2015: data availability and gaps on 54 major foods, beverages and nutrients among 5.6 million children and adults from 1,220 surveys worldwide. *In Review.* 2020.
3. Finucane MM, Stevens GA, Cowan MJ, et al. National, regional, and global trends in body-mass index since 1980: systematic analysis of health examination surveys and epidemiological studies with 960 country-years and 9·1 million participants. *Lancet.* 2011;377(9765):557-567.
4. Flaxman AD, Vos T, Murray CJ. *An Integrative Metaregression Framework for Descriptive Epidemiology.* University of Washington Press; 2015.
5. Carpenter B, Gelman A, Hoffman MD, et al. Stan: A Probabilistic Programming Language. *Journal of Statistical Software.* 2017;76(1):1 - 32.
6. Stan Development Team. *Stan modeling language users guide and reference manual, version 2.27.* 2021.
7. Stan Development Team. *RStan: the R interface to Stan.* 2019.
8. Homan MD, Gelman A. The No-U-turn sampler: adaptively setting path lengths in Hamiltonian Monte Carlo. *J Mach Learn Res.* 2014;15(1):1593–1623.
9. Radford N. MCMC using Hamiltonian dynamics. In: Brooks S, Gelman A, Jones G, Meng X-L, eds. *Handbook of Markov Chain Monte Carlo.* 1st Edition ed. New York: Chapman and Hall/CRC; 2011.
10. Gelman A, Pardoe L. Bayesian measures of explained variance and pooling in multilevel (hierarchical) models. *Technometrics.* 2006;48(2):241-251.
11. McElreath R. Statistical Rethinking: A Bayesian Course with Examples in R and Stan. *Text Stat Sci.* 2016:1-464.
12. Wagner T, Diefenbach DR, Christensen SA, Norton AS. Using Multilevel Models to Quantify Heterogeneity in Resource Selection. *J Wildlife Manage.* 2011;75(8):1788-1796.
